# Deprotonation-controlled copper-free Pd-catalyzed Sonogashira coupling *versus* the Kumada–Tamao–Corriu reaction: a DFT investigation toward anticancer carborane alkynes

**DOI:** 10.1039/d5ra09733a

**Published:** 2026-04-21

**Authors:** Elham Soltani, Mehdi Bayat

**Affiliations:** a Department of Inorganic Chemistry, Faculty of Chemistry and Petroleum Sciences, Bu-Ali Sina University Hamedan 65178-38683 Iran; b School of Chemistry, College of Science, University of Tehran Tehran Iran bayatm@ut.ac.ir mehdi806@gmail.com

## Abstract

Carborane derivatives have emerged as valuable hydrophobic pharmacophores in medicinal chemistry owing to their unique electronic characteristics and rigid three-dimensional architectures. Here, density functional theory (DFT) calculations are used to dissect the copper-free Pd-catalyzed Sonogashira coupling that leads to 3-quinolylethynyl carborane alkynes and to benchmark this transformation against the Pd-catalyzed Kumada–Tamao–Corriu reaction for B–C bond formation. The Sonogashira manifold is analyzed in terms of four limiting scenarios: a carbopalladation route, cationic and anionic deprotonation pathways and an ionic pathway involving base-assisted chloride substitution at palladium. The carbopalladation route, although overall exergonic, is rendered kinetically inaccessible by a prohibitively high barrier for vinylic C–H deprotonation, whereas the cationic and ionic deprotonation mechanisms display substantially lower, but still moderate, activation free energies. In contrast, the anionic deprotonation pathway—initiated by base-promoted deprotonation of the terminal alkyne and electronic stabilization of the resulting acetylide by the 3-quinolyl group—features the lowest overall Gibbs free energy barrier and therefore emerges as the dominant mechanism under copper-free conditions. Comparison with the Kumada cycle shows that, while the latter is thermodynamically feasible, the key B–C bond-forming reductive elimination step is associated with a significantly higher barrier and delivers a less stable product than the corresponding Sonogashira outcome. Taken together with available experimental data, these results indicate that copper-free Sonogashira coupling with Pd(PPh_3_)_2_Cl_2_ is both kinetically and thermodynamically preferred for accessing 3-quinolylethynyl carborane alkynes, highlight these motifs as promising anticancer pharmacophores and provide mechanistic guidelines for the rational design of B–C bond-forming reactions in carborane chemistry.

## Abstract 2

Carborane derivatives have emerged as valuable hydrophobic pharmacophores in medicinal chemistry owing to their unique electronic characteristics and rigid three-dimensional architectures.

## Graphical abstract

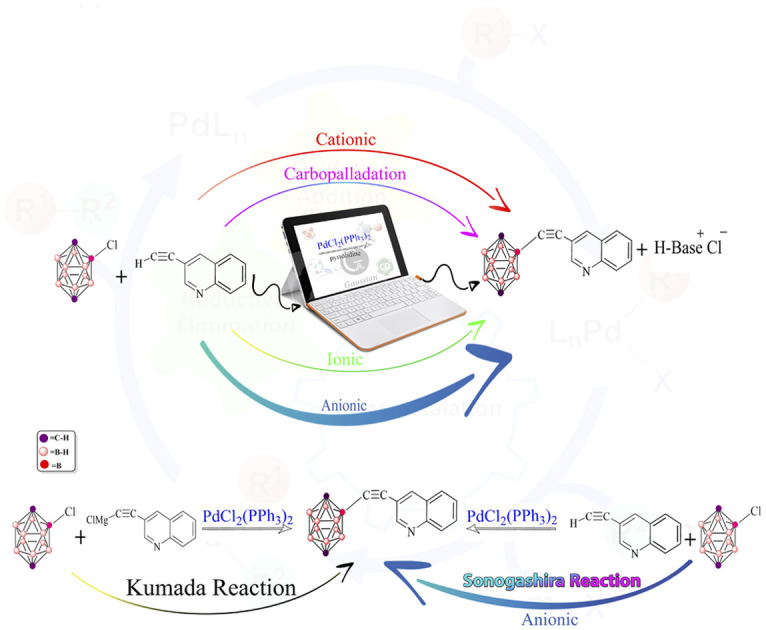

## 1. Introduction

Building larger, more complex molecules from simple fragments is one of the central goals of synthetic chemistry, and metal-catalyzed cross-coupling reactions have become the method of choice for achieving this efficiently.^1–7^ Such transformations rely on adaptable catalysts and reagents that can operate under mild conditions and tolerate a broad range of functional groups. In this context, palladium-catalyzed cross-coupling protocols have proved particularly powerful, enabling the preparation of targets that span from active pharmaceutical ingredients to advanced functional materials.^8–16^ The emergence of modern theoretical and computational tools has greatly deepened our understanding of how these Pd-catalyzed reactions proceed at the molecular level. In particular, density functional theory (DFT) now allows realistic modelling of entire catalytic cycles, providing quantitative information on energy barriers, relative stabilities of intermediates and the influence of ligands and solvents on catalytic turnover. These insights are not only useful for rationalizing experimental observations, but also for anticipating the behaviour of new catalytic systems before they are explored in the laboratory.^17–23^ Palladium catalyzes efficient carbon–carbon and carbon–heteroatom bond formation in cross-coupling reactions, typically offering mild reaction conditions, high yields and excellent selectivity.^24–29^

**Fig. 1:**
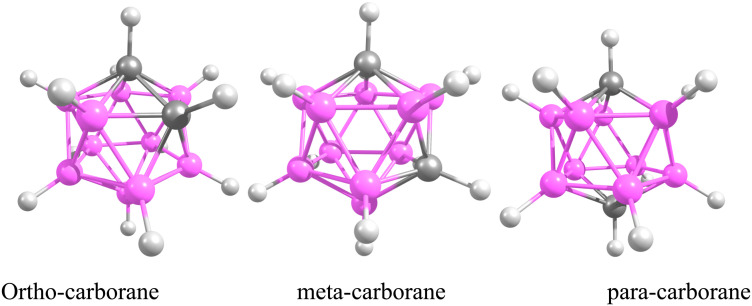
The three isomeric *closo*-carboranes with formula C_2_B_10_H_12_, depicted in their icosahedral structures.

**Scheme 1:**
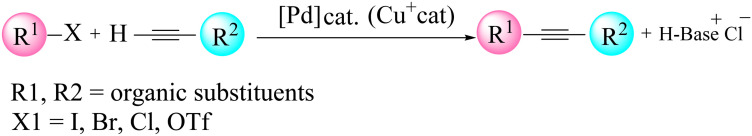
Overall representation of the Pd-catalyzed Sonogashira reaction.

**Fig. 2:**
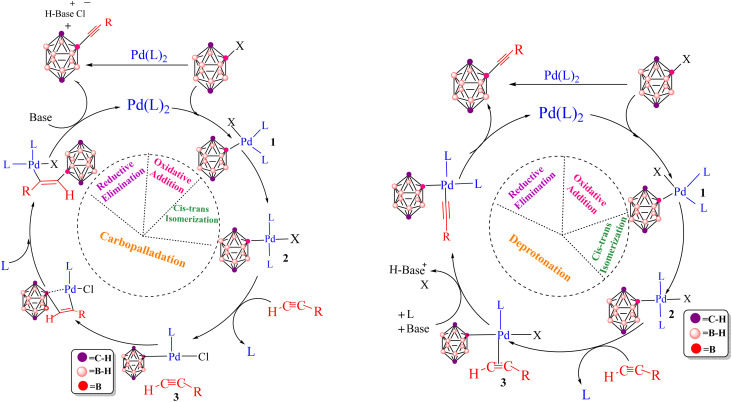
Proposed reaction pathways for the copper-free Sonogashira reaction: carbopalladation (left) and deprotonation-based mechanisms (right).

**Fig. 3:**
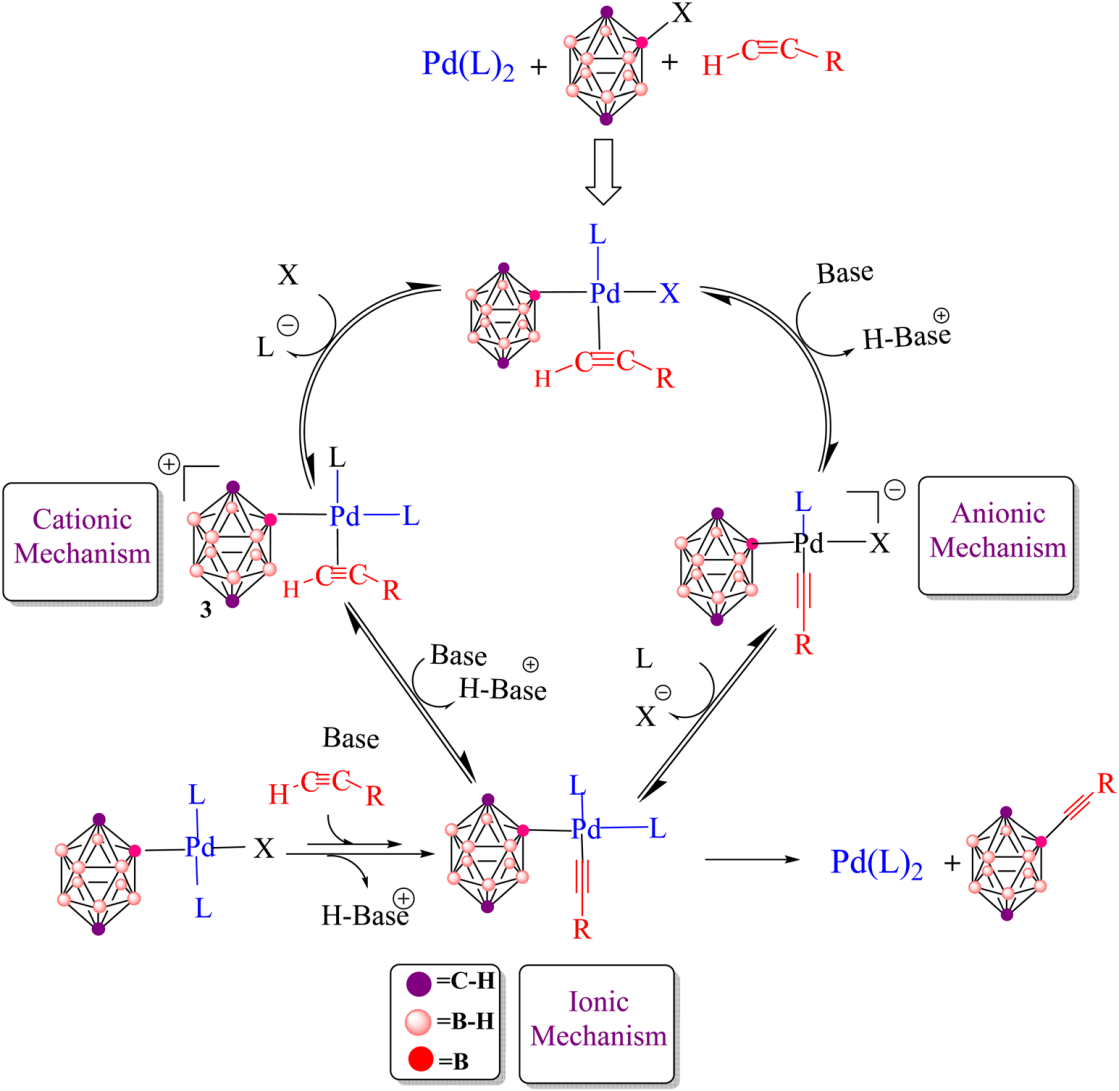
Copper-free Sonogashira manifold relying on direct alkyne coordination and proceeding *via* cationic, anionic and ionic pathways.

**Scheme 2:**
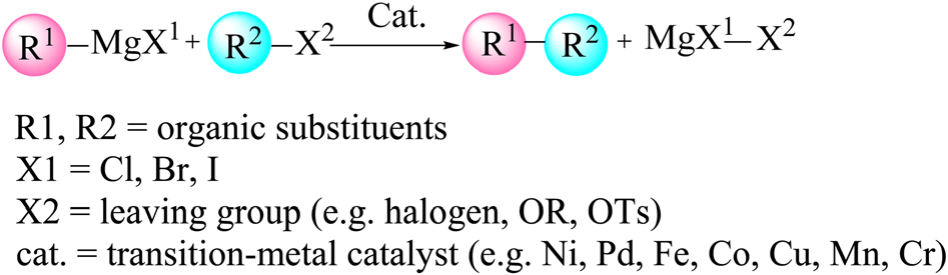
General framework for the Pd-catalyzed Kumada–Tamao–Corriu (Kumada) reaction.

In many cases, only low catalyst loadings are required, which reduces both cost and waste generation and makes these methods attractive from a green chemistry perspective. Recent work has focused on developing well-defined molecular precatalysts and on extending Pd-catalyzed cross-coupling chemistry beyond classical organic substrates to more unconventional frameworks, most notably boron-rich clusters such as carboranes.^30–49^ A prominent polyhedral boron cluster of medicinal significance is the icosahedral *closo*-C_2_B_10_H_12_, which exists in three isomeric forms: *ortho*- (1,2-C_2_B_10_H_12_), *meta*- (1,7-C_2_B_10_H_12_) and *para*-carborane (1,12-C_2_B_10_H_12_) (Fig. 1).^50–53^ The combination of an electron-deficient framework with three-dimensional aromaticity makes these clusters particularly appealing building blocks for functionalization by transition-metal-catalyzed cross-coupling, allowing the attachment of organic fragments while tuning the overall electronic profile. Pd-catalyzed aryl halide cross-couplings and related metal-mediated transformations have, in this way, opened up straightforward synthetic access to a wide range of functionalized carboranes that are now being explored in both materials science and medicinal chemistry.^54–59^ Carboranes are especially attractive for biomedical applications because they combine several advantageous features: high boron content (ideal for boron neutron capture therapy, BNCT), exceptional thermal and chemical stability, marked hydrophobicity and finely tunable electronic properties.^60–81^ Their ability to form robust metallacarboranes and water-soluble derivatives further supports their use as pharmacophores in anticancer drug candidates, targeted delivery systems and molecular imaging agents. This study examines the Pd-catalyzed Sonogashira and Kumada cross-coupling reactions for forming B–C bonds, thereby enabling the incorporation of organic fragments into carborane frameworks. The Sonogashira reaction, in particular, is recognized as one of the most versatile tools for constructing C(sp)–C(sp^2^) bonds in organic synthesis (Scheme 1).^82–87^ In copper-free Sonogashira reactions (HCS protocol), several mechanistic pathways have been proposed (Fig. 2), including carbopalladation, deprotonation and ionic routes.^88,89^ More recently, an alternative bimetallic Pd/Pd pathway has been suggested, in which two palladium centres cooperate through an intermetallic, transmetallation-like step that effectively replaces the role of copper in the traditional mechanism and helps to account for efficient catalytic turnover under copper-free conditions. In all of these scenarios, the catalytic cycle begins with oxidative addition of the organohalide (carborane–X) to a Pd(0)L_2_ complex, followed by coordination of the alkyne to give a π-complex (Fig. 2). From this point, the mechanisms diverge. In the carbopalladation pathway, the alkyne inserts directly into the Pd–C bond, whereas in the deprotonation pathway a base first abstracts the proton from the terminal alkyne to generate a nucleophilic alkynyl species that subsequently binds to Pd. It is important to emphasize that, in copper-free systems, this step is not a genuine transmetalation event, because no second metal is present; rather, it is more accurately described as an inner-sphere ligand exchange followed by intramolecular acetylide transfer at palladium. Distinguishing between these different mechanistic pictures is essential for understanding reactivity, regioselectivity and kinetic control. Significant contributions to clarifying these mechanisms have been made by Jutand *et al.*^90,91^ and Mårtensson *et al.*^92^ The latter provided experimental evidence that argues against a purely carbopalladation-based mechanism and instead supports two competing deprotonation pathways—cationic and anionic—operating in the palladium-catalyzed, copper-free Sonogashira reaction (Fig. 3). Despite these advances, truly side-by-side computational comparisons of the Sonogashira and Kumada–Tamao–Corriu (KTC) mechanisms within a single, internally consistent framework are still relatively rare. The present work therefore aims to provide a systematic theoretical assessment of the relative favourability of copper-free Sonogashira *versus* Kumada cross-couplings, using both thermodynamic and kinetic criteria, for the synthesis of 3-quinolylethynyl carborane, a compound with potential anticancer activity. To this end, DFT calculations including explicit solvation and entropy corrections are used to construct Gibbs free energy profiles for several candidate pathways, namely carbopalladation, cationic and anionic deprotonation and an ionic route. The resulting picture not only clarifies the energetics of the competing mechanisms, but also offers deeper mechanistic insight into Pd-mediated B–C coupling in boron cluster chemistry, which can in turn guide future experimental exploration. The Kumada reaction, which couples organohalides with Grignard reagents (RMgX), represents a complementary and robust strategy for forging C–C bonds and accessing key intermediates of biologically active molecules (Scheme 2).^93–97^ The Schlenk equilibrium (2 RMgCl ⇌ R_2_Mg + MgCl_2_) represents a potential source of complexity in Grignard-mediated couplings. Our computational protocol employs R–MgCl reagents in 1,4-dioxane – a strong donor solvent that disrupts Mg–X–Mg bridges through competitive coordination. This approach is supported by experimental precedent: Sinha *et al.* report 91% isolated yield and >95% Br-selectivity using 4-cyanophenyl magnesium chloride (a related Grignard reagent) under similar conditions.^98^ By comparing its reaction energetics and transition states with those of the copper-free Sonogashira coupling, valuable information is obtained on how nucleophile strength, potential metal–metal cooperation and solvent effects together dictate the overall catalytic efficiency of Pd-mediated systems.

## 2. Computational details

All quantum mechanical calculations in this research were conducted at the DFT level using the dispersion-corrected B3LYP (B3LYP-D3) functional,^99^ as implemented in the Gaussian 09 (Revision D) software package,^100^ with 1,4-dioxane as the solvent. Implicit solvation models such as PCM, COSMO and SMD were used to describe the solvent effects of 1,4-dioxane, which is a polar aprotic medium with suitable polarity, chemical stability and solubility for organic substrates. For geometry optimizations, the 6–31G*(d,p) basis set^101^ was employed for H, B, C, N, P and Cl atoms, whereas the LANL2DZ effective core potential^102,103^ was used for Pd and Mg atoms. This mixed basis set is referred to as BSL. The chosen computational level has been extensively applied in theoretical studies of related Pd-catalyzed cross-coupling reactions and affords reliable structural and energetic data.^104–108^ Prior to selecting the final computational protocol, several alternative functionals were tested in order to evaluate their suitability for the present catalytic system. In particular, CAM-B3LYP and M06, which are often recommended for systems with significant charge-transfer character and dispersion interactions, were explored during the initial stages of this work. However, for the large catalytic models considered here (exceeding 110 atoms and containing flexible Pd–phosphine coordination environments), these functionals exhibited significant convergence difficulties. In particular:

• CAM-B3LYP failed to converge for several key transition states associated with the Pd–phosphine catalytic manifold.

• M06 calculations encountered repeated SCF and geometry convergence failures for multiple stationary points along the catalytic pathway.

To assess the potential influence of long-range exchange effects, single-point energy calculations were performed using the CAM-B3LYP functional on the B3LYP-D3 optimized geometries of representative intermediates and transition states along the Kumada pathway. Although the absolute energies differ from those obtained at the B3LYP-D3 level, the relative ordering of intermediates and transition states remains unchanged. In particular, the same transition state is identified as the highest-energy point along the pathway. These results indicate that the proposed mechanistic conclusions are qualitatively robust with respect to the choice of exchange–correlation functional. A detailed comparison of the two computational approaches is provided in the SI (Table S2).

All reactants, intermediates and products were fully optimized at the B3LYP-D3/BSL level in 1,4-dioxane without imposing symmetry constraints, and no spurious interactions involving the hydrogen atoms of the triphenylphosphine (PPh_3_) ligands were detected during optimization. Harmonic vibrational frequency calculations at the same level were used to confirm that minima exhibit no imaginary frequencies, whereas each transition state has a single imaginary frequency. To further validate the transition states, intrinsic reaction coordinate (IRC) calculations were carried out at the B3LYP-D3/BSL level, ensuring that each saddle point connects the appropriate reactant and product minima along the reaction pathway (Fig. S7).^109^

Single-point electronic energies were subsequently refined at the B3LYP-D3/def2-TZVPP level on the B3LYP-D3/BSL-optimized geometries. Gibbs free energies in solution were obtained by combining these B3LYP-D3/def2-TZVPP electronic energies with thermal and entropic corrections evaluated at the B3LYP-D3/BSL level. To gain insight into electronic structure and bonding changes along the reaction coordinates, natural bond orbital (NBO) analyses were performed at the B3LYP-D3/def2-TZVPP//B3LYP-D3/BSL level, and Wiberg bond indices (WBIs) were extracted as quantitative descriptors of bond formation and cleavage in the key transition states.^110^

## 3. Results and discussion

### 3.1. Copper-free Pd-catalyzed B–C bond formation in carborane-based Sonogashira coupling

**Scheme 3:**
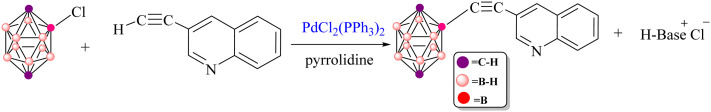
Copper-free Pd-catalyzed Sonogashira coupling between 2-Cl-*p*-carborane and 3-ethynylquinoline.

**Fig. 4:**
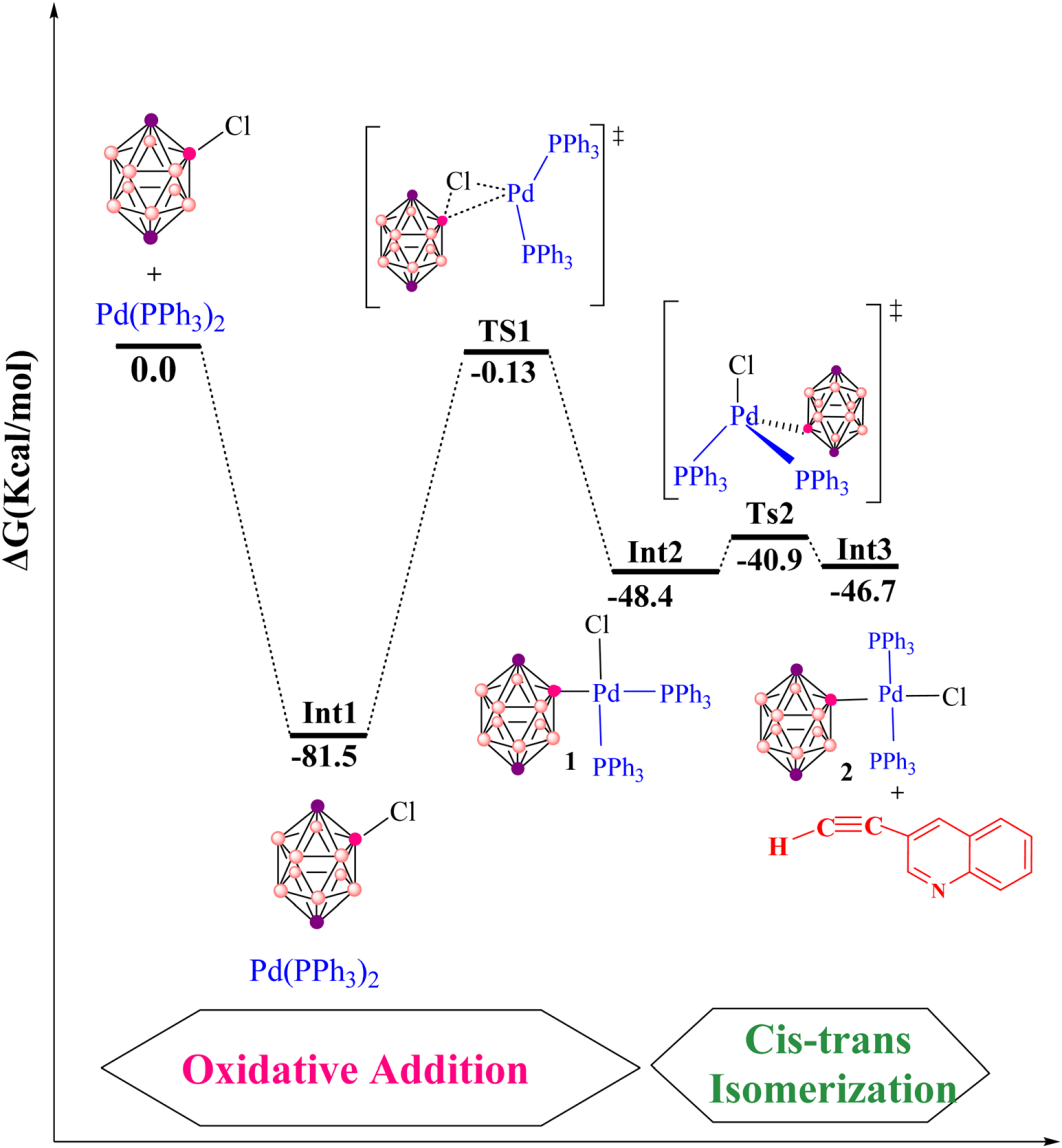
Gibbs free-energy profile for the Pd-catalyzed copper-free Sonogashira reaction, including oxidative addition and *cis*–*trans* isomerization, computed at the B3LYP-D3/def2-TZVPP//B3LYP-D3/BSL level in 1,4-dioxane.

**Fig. 5:**
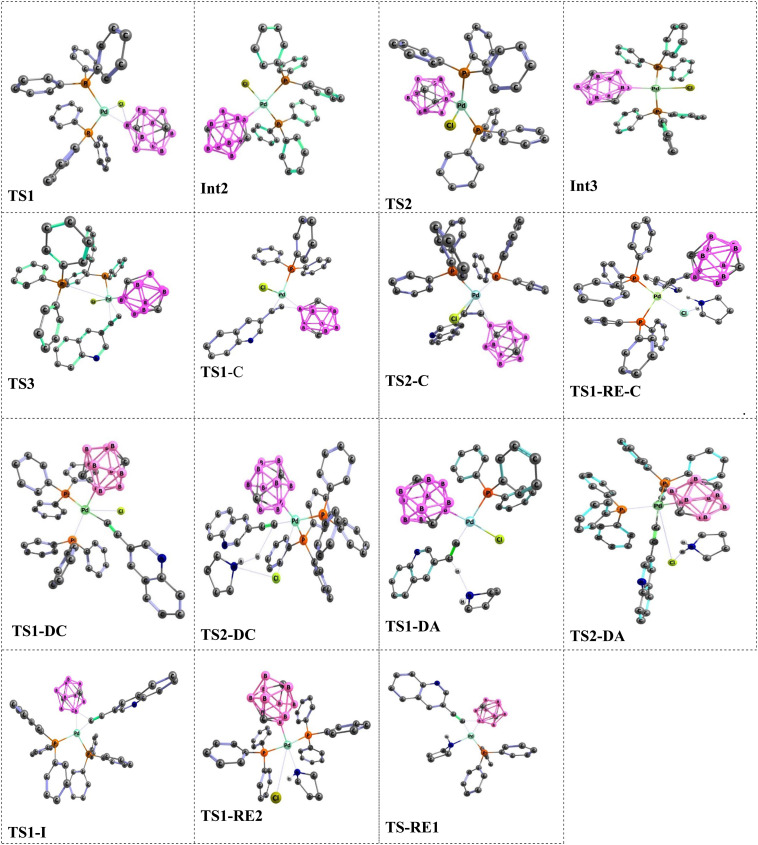
Optimized geometries of key intermediates (Int2, Int3) and transition states along the copper-free Sonogashira pathway to 3-quinolylethynyl carborane, obtained at the B3LYP-D3/BSL level in 1,4-dioxane.

**Fig. 6:**
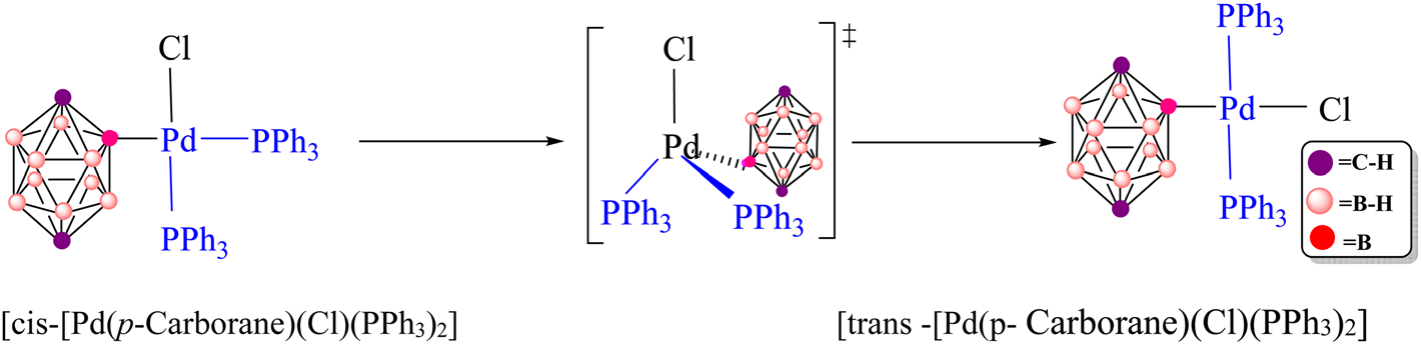
*Cis*–*trans* isomerization of [Pd(*p*-carborane)(Cl)(PPh_3_)_2_] at palladium.

The Sonogashira reaction mechanism consists of four elementary steps: oxidative addition (OA), *cis*–*trans* isomerization, transmetalation (TM) and reductive elimination (RE) (Fig. 2). In this study, theoretical investigations were conducted on carborane substrates utilizing the Pd(PPh_3_)_2_Cl_2_ complex as the catalyst for the Sonogashira cross-coupling reaction. The geometry of the Pd(PPh_3_)_2_Cl_2_ complex is approximately square planar around the palladium center, a characteristic feature commonly observed in many palladium(ii) complexes.^111–115^ The Sonogashira reaction mechanism was further elucidated using 3-ethynylquinoline and 2-Cl-*p*-carborane as the coupling partners, with pyrrolidine serving as the base and 1,4-dioxane as the solvent (Scheme 3).^116^ The analysis of the oxidative addition step is crucial for understanding the initial interactions between the reactants and the palladium catalyst. As depicted in Fig. 4, the first step of the reaction involves the oxidative addition of 2-Cl-*p*-carborane to the palladium complex [Pd(PPh_3_)_2_]. The starting reagents, [Pd(PPh_3_)_2_] and 2-Cl-*p*-carborane, are assigned a relative energy of 0.0 kcal mol^−1^, providing a reference point for evaluating the energy changes along the reaction pathway (The optimized structures of Int1, Int2, TS1 and TS2 are shown in Fig. 5). Upon interaction with the palladium center, an intermediate Int1 is formed, stabilized at −81.5 kcal mol^−1^. This substantial drop in energy indicates that formation of Int1 is highly favorable, reflecting strong interactions between the palladium center and the carborane moiety. The subsequent step involves oxidative addition of the B(carborane)–Cl bond of 2-Cl-*p*-carborane to the palladium center. During this process, the B(carborane)–Cl bond is cleaved and new Pd–B(carborane) and Pd–Cl bonds are formed, resulting in an increase in the oxidation state of palladium from 0 to +2. The transition state for this step (TS1) has a relative energy of −0.13 kcal mol^−1^, indicating that it is slightly more stable than the separated reactants but still represents a high-energy configuration along the reaction coordinate. Following TS1, another intermediate Int2 is generated with a relative energy of −48.4 kcal mol^−1^, further stabilizing the system as it progresses toward product formation. Importantly, in this transition state TS1, the Pd–B(carborane), Pd–Cl and B(carborane)–Cl bond lengths are 2.0, 3.1 and 3.0 Å, respectively. These structural parameters are consistent with partial cleavage of the B–Cl bond and concurrent formation of the Pd–B and Pd–Cl bonds. It is worth noting that the oxidative addition step is a common feature in all Sonogashira reaction pathways considered in this work. The next step, a *cis*–*trans* isomerization (Fig. 4), results in a product with a *trans* configuration. The isomerization between square-planar *cis* and *trans* isomers of metal complexes, particularly in the context of oxidative addition and transmetalation processes, is a well-recognized feature of cross-coupling catalysis. The *cis* isomer is often formed as an intermediate during oxidative addition, yet it is rarely isolated. This suggests that, while it may be a transient species, the *trans* isomer plays a more pivotal role in subsequent catalytic steps, such as transmetalation. The conversion from the square-planar *cis* isomer to the *trans* isomer occurs *via* a four-coordinate mechanism. This pathway enables a direct transformation without altering the coordination number of the metal, which is crucial for maintaining the stability of the complex throughout the reaction^117^ (Fig. 6). This step proceeds through transition state TS2 (−42.1 kcal mol^−1^), which requires an activation barrier of 6.23 kcal mol^−1^ (Fig. 4). As shown in Fig. S1, this contrasts with intermediate Int2 (*cis*-[Pd(*p*-carborane)(Cl)(PPh_3_)_2_]), which adopts a *cis* configuration. Int2 exhibits Pd–B(carborane), Pd–Cl, Pd–P(1) and Pd–P(2) bond lengths of 2.1, 2.4, 2.7 and 2.3 Å, respectively, along with P(1)–Pd–P(2) and Cl–Pd–B bond angles of 103.19° and 91.42°, respectively.

In comparison, TS2 shows Pd–B(carborane), Pd–Cl, Pd–P(1) and Pd–P(2) bond lengths of 2.0, 2.6, 2.4 and 2.4 Å, respectively, and P(1)–Pd–P(2) and Cl–Pd–B bond angles of 146.80° and 132.23°, respectively. The optimized TS2 thus shows moderate changes in bond lengths and angles compared to Int2, reflecting the structural rearrangements associated with *cis*–*trans* isomerization. This process ultimately leads to the formation of the *trans* isomer Int3 (*trans*-[Pd(*p*-carborane)(Cl)(PPh_3_)_2_]), which has an energy of −46.7 kcal mol^−1^. Int3 exhibits Pd–B(carborane), Pd–Cl, Pd–P(1) and Pd–P(2) bond lengths of 2.1, 2.5, 2.4 and 2.4 Å, respectively, along with P(1)–Pd–P(2) and Cl–Pd–B bond angles of 154.79° and 153.09°, respectively. The *trans* isomer is therefore more stable than both the *cis* isomer and TS2 (−46.7 *vs.* −48.4 and −42.1 kcal mol^−1^), suggesting that it is thermodynamically favored as a resting state for subsequent reactions. Notably, such isomerization is a consistent feature in all Sonogashira reaction pathways. As previously discussed, oxidative addition followed by *cis*-to-*trans* isomerization constitutes a common initial sequence in all proposed pathways for the Sonogashira reaction in the absence of copper. Therefore, we proceed to analyze the subsequent steps of the reaction and compare the proposed pathways for the Sonogashira reaction, starting from complex *trans*-[Pd(*p*-carborane)(Cl)(PPh_3_)_2_].

### 3.2. Carbopalladation-based copper-free Sonogashira pathway to 3-quinolylethynyl carborane

**Fig. 7:**
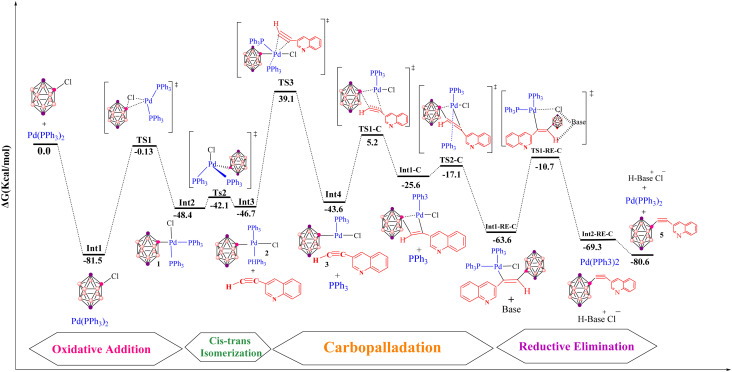
Gibbs free-energy diagram for the carbopalladation-based copper-free Sonogashira pathway, catalyzed by Pd with pyrrolidine as base, computed at the B3LYP-D3/def2-TZVPP//B3LYP-D3/BSL level in 1,4-dioxane.

Fig. 7 summarizes the carbopalladation-type pathway explored for the copper-free Sonogashira reaction of 3-ethynylquinoline, catalyzed by the Pd(PPh_3_)_2_Cl_2_ complex. This mechanism starts with substitution of one phosphine ligand by 3-ethynylquinoline to form intermediate Int4. This associative substitution is endergonic, with a relative free energy of −43.6 kcal mol^−1^ (referenced to the separated reactants), a trend similar to that observed for the corresponding deprotonation-based pathways. The transition state for this step, TS3, lies at 39.1 kcal mol^−1^ and is characterized by Pd–P(1), Pd–C(1) and Pd–C(2) distances of 5.6, 2.2 and 2.5 Å, respectively. Following formation of the Pd–alkyne complex, the carbopalladation step proceeds *via* transition state TS1-C, which also has a relative energy of −43.6 kcal mol^−1^. In TS1-C, the Pd–B(carborane) bond length is 2.2 Å, whereas the Pd–C(2) and B–C(1) distances are both 2.1 Å, consistent with simultaneous formation of Pd–C and B–C bonds along the alkyne. The reaction then evolves to a highly stabilized intermediate, RE-Int1-C, located at −63.6 kcal mol^−1^ relative to the reactants, with an overall barrier of only 8.5 kcal mol^−1^ for this transformation. Subsequent coordination of a phosphine ligand occurs through TS2-C (−25.6 kcal mol^−1^), featuring Pd–B and Pd–P(1) distances of 3.7 and 2.4 Å, respectively. In the final step of this pathway, the alkenyl fragment in RE-Int1-C undergoes deprotonation by an external base *via* transition state RE-TS1-C, yielding the coupled product 3-quinolylethynyl carborane and regenerating the active catalyst. However, this deprotonation step carries a very high activation barrier of 52.87 kcal mol^−1^, reflecting both the substantial stabilization of RE-Int1-C and the intrinsic difficulty of deprotonating a vinylic C–H bond. Overall, although the carbopalladation pathway is globally exergonic (Δ*G* = −80.6 kcal mol^−1^), the large barrier associated with the final deprotonation renders this mechanism kinetically inaccessible under typical copper-free Sonogashira conditions. This theoretical conclusion is consistent with the experimental observations of Mårtensson *et al.*,^92^ who reported that a complex analogous to RE-Int1-C failed to deliver the cross-coupled product under standard reaction conditions.

### 3.3. Deprotonation-driven copper-free Sonogashira manifold under Pd catalysis

The copper-free Sonogashira reaction was also examined through a mechanism in which C–H deprotonation plays the key activating role. As outlined in the introduction, this manifold involves ligand exchange at the palladium centre and can proceed through two distinct pathways, cationic and anionic, depending on the sequence of ligand substitution and deprotonation events (Fig. 3).^92,118,119^ The order of these steps determines which deprotonation pathway is energetically preferred. In the cationic pathway, ligand substitution at complex 3 first generates a cationic palladium species, *cis*-[Pd(carborane)(alkyne)(L)_2_]^+^. The coordinated alkyne is then deprotonated by an external base, and the resulting acetylide complex undergoes reductive elimination to furnish the cross-coupled product. In contrast, the anionic pathway begins with deprotonation of the free alkyne, giving an anionic complex *cis*-[Pd(carborane)(acetylide)(X)(L)]^−^ (Fig. 3). After this initial deprotonation, ligand substitution at palladium takes place, followed by reductive elimination to deliver the desired product.

#### 3.3.1. Cationic deprotonation sequence in the Pd-catalyzed copper-free Sonogashira manifold

**Fig. 8:**
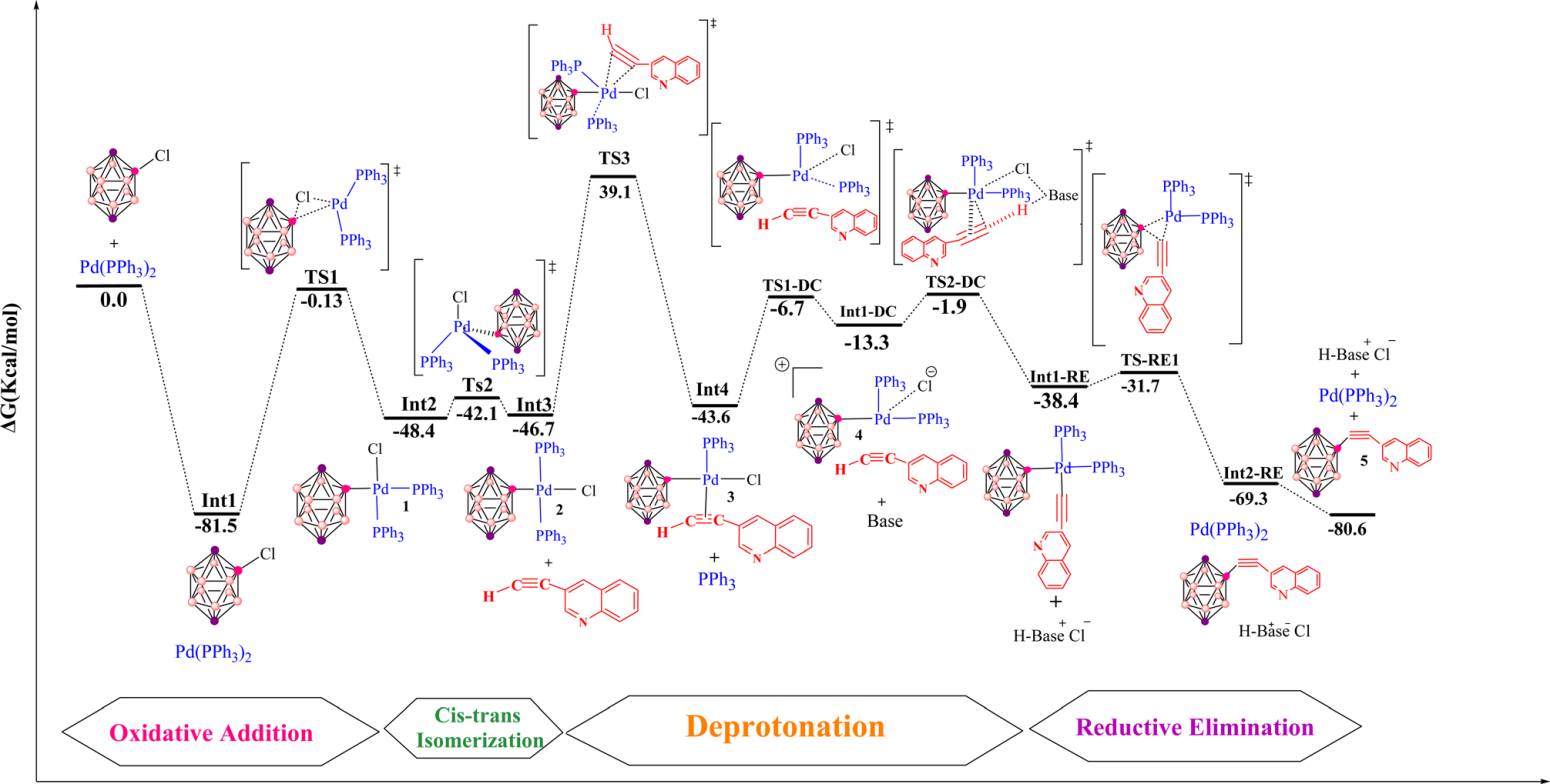
Gibbs free-energy diagram for the cationic copper-free Sonogashira pathway, catalyzed by Pd with pyrrolidine as base, computed at the B3LYP-D3/def2-TZVPP//B3LYP-D3/BSL level in 1,4-dioxane.

The cationic pathway of the copper-free Sonogashira reaction between 3-ethynylquinoline and 2-Cl-*p*-carborane proceeds through a characteristic sequence of ligand substitution and deprotonation steps. The computed Gibbs free energy profile (Fig. 8) reveals the key intermediates and transition states that define this mechanism. The reaction begins with formation of a cationic palladium complex, followed by substitution of the chloride ligand by a phosphine ligand to give the ion-paired intermediate Int1-DC at −13.3 kcal mol^−1^. The associated transition state TS1-DC has a barrier of 36.9 kcal mol^−1^ and features Pd–P(1) and Pd–Cl distances of 2.6 and 3.7 Å, respectively. Subsequent deprotonation of the coordinated alkyne occurs *via* TS2-DC, located at −1.9 kcal mol^−1^. This transition state is characterized by Pd–Cl, C(1)–H, N–H and Cl–N distances of 4.1, 2.8, 2.2 and 3.5 Å, respectively, and leads to intermediate RE-Int1, in which the two organic fragments adopt a *cis* arrangement around palladium. The final step involves reductive elimination from RE-Int1, with a Gibbs energy of −38.4 kcal mol^−1^. This process proceeds through TS-RE1 (−31.7 kcal mol^−1^), where the Pd–B, Pd–C(1) and B–C(1) distances are 2.2, 1.9 and 2.0 Å, respectively, and furnishes the cross-coupled product together with regeneration of the palladium catalyst. The overall Gibbs free energy change for the cationic pathway is −80.6 kcal mol^−1^. Fig. 5 depicts the optimized geometries of TS1-DC, TS2-DC and TS-RE1, highlighting the structural features that govern the reaction dynamics. Comparison of this energy profile with that of the anionic pathway (Section 3.3.2) underscores the differences in deprotonation sequences and ligand-substitution events, thereby refining the mechanistic picture of copper-free Sonogashira reactions.

#### 3.3.2. Anionic deprotonation sequence in the Pd-catalyzed copper-free Sonogashira manifold

**Fig. 9:**
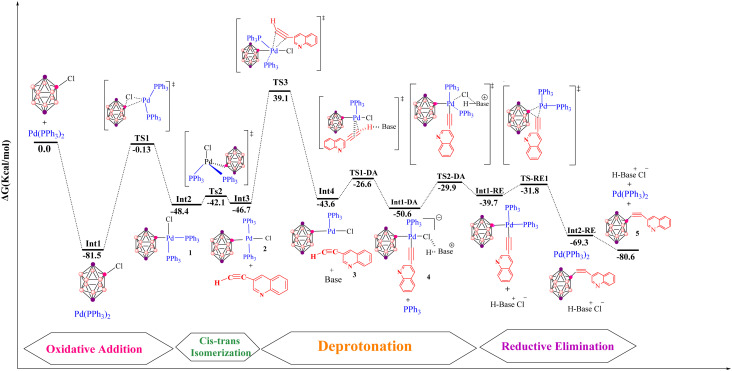
Gibbs free-energy diagram for the anionic copper-free Sonogashira pathway, catalyzed by Pd with pyrrolidine as base, computed at the B3LYP-D3/def2-TZVPP//B3LYP-D3/BSL level in 1,4-dioxane.

The anionic pathway of the Sonogashira reaction features a deprotonation sequence that differs fundamentally from the cationic mechanism. In this case, deprotonation of the alkyne in complex 3 by an external base is the initial step, setting off a series of transformations that ultimately yield the cross-coupled product. As depicted in Fig. 9, the alkyne is deprotonated *via* transition state TS1-DA, located at −26.6 kcal mol^−1^. This step affords the ion-pair intermediate Int1-DA at −50.6 kcal mol^−1^, comprising an anionic palladium acetylide complex and the protonated base. In TS1-DA, the Pd–C(1), C(2)–H and H–N distances are 1.9, 1.1 and 1.8 Å, respectively. Following formation of Int1-DA, a chloride-for-phosphine substitution takes place, proceeding through TS2-DA at −29.9 kcal mol^−1^ and leading to intermediate RE-Int1 with a Gibbs energy of −39.7 kcal mol^−1^. TS2-DA is characterized by Pd–P(1), Pd–Cl and Cl–H distances of 2.6, 6.3 and 1.9 Å, respectively. In the final step, reductive elimination from RE-Int1 generates the cross-coupled product and regenerates the palladium catalyst; this step is thermodynamically highly favorable, with an overall Gibbs free energy of −80.6 kcal mol^−1^. The optimized geometries of TS1-DA and TS2-DA are shown in Fig. 5. Taken together, the anionic pathway underscores how the sequence of alkyne deprotonation and ligand substitution modulates the energetics and efficiency of the copper-free Sonogashira reaction. The maximum activation free energy along the anionic Sonogashira pathway corresponds to the TS2-DA transition state, which lies 20.7 kcal mol^−1^ above the reference intermediate Int1-DA. The free energies of these species are −29.9 kcal mol^−1^ for TS2-DA and −50.6 kcal mol^−1^ for Int1-DA, giving Δ*G*_max = 20.7 kcal mol^−1^. This value therefore represents the highest barrier along the productive anionic catalytic pathway relative to the resting-state intermediate Int1-DA.

#### 3.3.3. Comparison of the cationic and anionic deprotonation pathways

Computational results show that chloride–phosphine exchange at palladium is associated with sizeable energy barriers in both the cationic and anionic manifolds. In the cationic sequence, ligand substitution precedes alkyne deprotonation: in complex 3, this step proceeds *via* TS1-DC, and the corresponding Gibbs free energy barrier is within the range expected for a kinetically viable process under typical Sonogashira conditions. In contrast, in the anionic sequence, deprotonation of the alkyne occurs first, and the chloride-for-phosphine substitution takes place later, from Int1-DA *via* TS2-DA. The calculated overall Gibbs free energy barriers indicate that both pathways are, in principle, kinetically accessible. However, the free-energy profiles point to a clear preference for product formation along the anionic pathway, owing to the additional stabilization of the anionic intermediate by the 3-quinolyl–C≡C fragment. This conjugated group effectively delocalizes negative charge, lowering the energy of the anionic species and thereby rendering the anionic deprotonation sequence more favorable than the cationic alternative.

### 3.4. Ionic mechanism as an alternative pathway

**Fig. 10:**
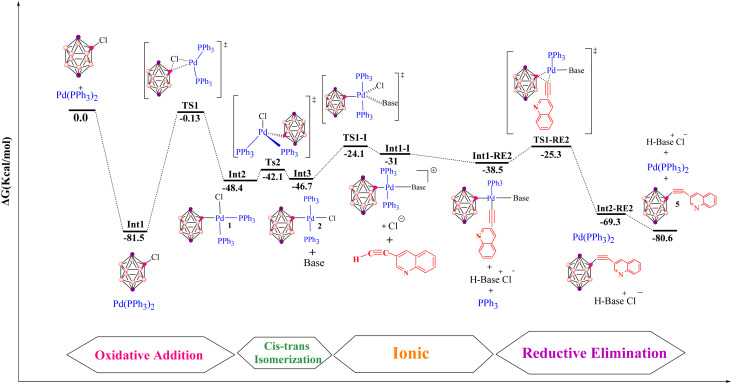
Gibbs free-energy diagram for the ionic copper-free Sonogashira pathway, catalyzed by Pd with pyrrolidine as base, computed at the B3LYP-D3/def2-TZVPP//B3LYP-D3/BSL level in 1,4-dioxane.

**Fig. 11:**
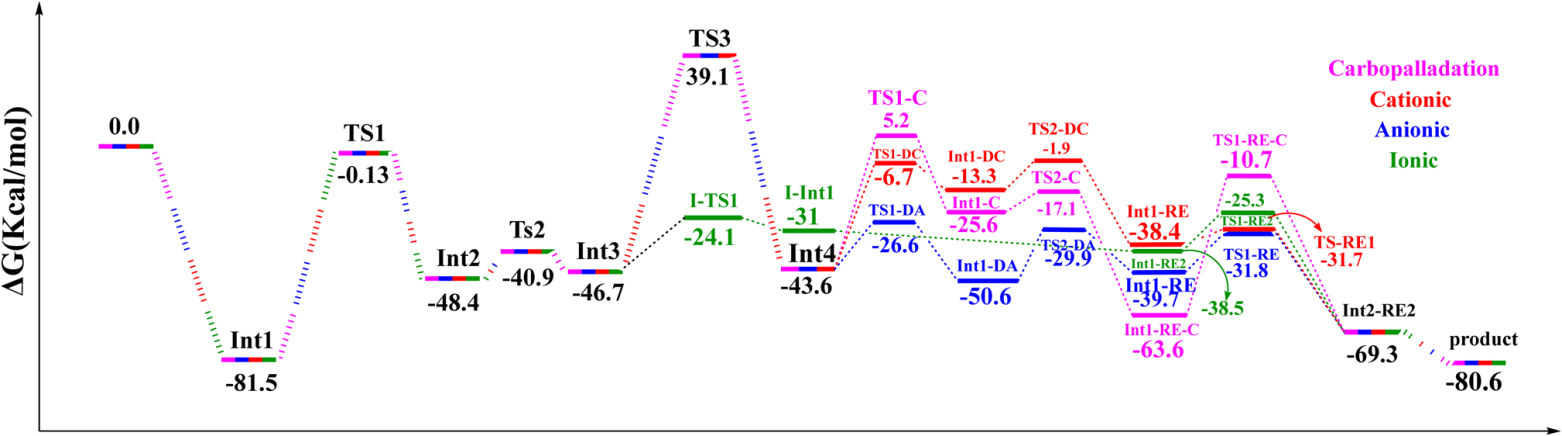
Gibbs free‑energy profiles for all proposed mechanisms of the copper‑free Sonogashira reaction, computed at the B3LYP‑D3/def2‑TZVPP//B3LYP‑D3/BSL level in 1,4‑dioxane.

The transmetalation step in Pd-catalyzed cross-couplings is a key elementary process that involves ligand exchange at the metal centre. In many cases, transmetalation proceeds through an ionic pathway in which an external ligand or base replaces a halide (such as chloride) at palladium, thereby facilitating cross-coupling reactivity.^120–122^ The nature of the external ligand can markedly influence both the kinetics and thermodynamics of this step, often enhancing overall catalytic efficiency. In the present system, transmetalation is described by an ionic mechanism in which ligand substitution occurs directly at the palladium centre. Unlike the cationic and anionic deprotonation pathways, where chloride dissociation occurs only after alkyne coordination, the ionic route features base-assisted chloride substitution at an early stage (Fig. 3). The resulting changes in the coordination environment around palladium play an important role in controlling reaction efficiency and selectivity. As shown in Fig. 10, the reaction of the cationic palladium complex Int1-I proceeds *via* TS1-I to give an intermediate with a Gibbs free energy of −31.0 kcal mol^−1^. TS1-I is characterized by Pd–N and Pd–Cl distances of 2.3 and 4.3 Å, respectively, indicating substantial reorganization at the metal centre. The corresponding activation barrier for this ionic transmetalation step is 22.1 kcal mol^−1^, notably lower than the barrier associated with alkyne coordination to Int3 *via* TS3, which suggests that transmetalation is relatively facile compared with subsequent steps in the mechanism. Computational analysis further shows that 3-ethynylquinoline can displace one of the phosphine ligands in Int1-I without encountering a significant energy barrier, affording an isoenergetic species denoted Int2-RE2. This essentially barrierless ligand exchange simplifies the catalytic manifold by removing additional energetic penalties for phosphine dissociation/association. From Int2-RE2, the system follows a common reductive-elimination pathway *via* TS1-RE2, which has an activation barrier of 13.2 kcal mol^−1^. TS1-RE2 exhibits B–C(1), Pd–B and Pd–C(1) distances of 2.0, 2.2 and 1.96 Å, respectively, and a C(1)–Pd–B bond angle of 67.1°. (The optimized structures of TS1-I and TS1-RE2 are depicted in Fig. 5). In summary, and as illustrated in Fig. 11, the carbopalladation pathway is disfavoured under standard conditions due to its prohibitively high activation barrier, whereas the cationic, anionic and ionic deprotonation manifolds all display substantially lower Gibbs free-energy barriers and are therefore mechanistically viable. The computed profiles indicate that these three pathways can, in principle, compete, and that subtle changes in solvent, ligands, substrates or base may shift the mechanistic preference toward one route or another. In the present case, the anionic pathway emerges as both thermodynamically and kinetically most favourable.

### 3.5. Pd-catalyzed Kumada B–C bond-forming reaction

**Scheme 4:**
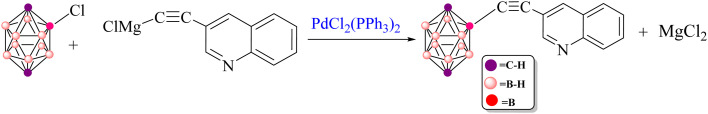
Pd-catalyzed Kumada coupling between 2-Cl-*p*-carborane and 3-quinolylethynyl magnesium chloride.

**Fig. 12:**
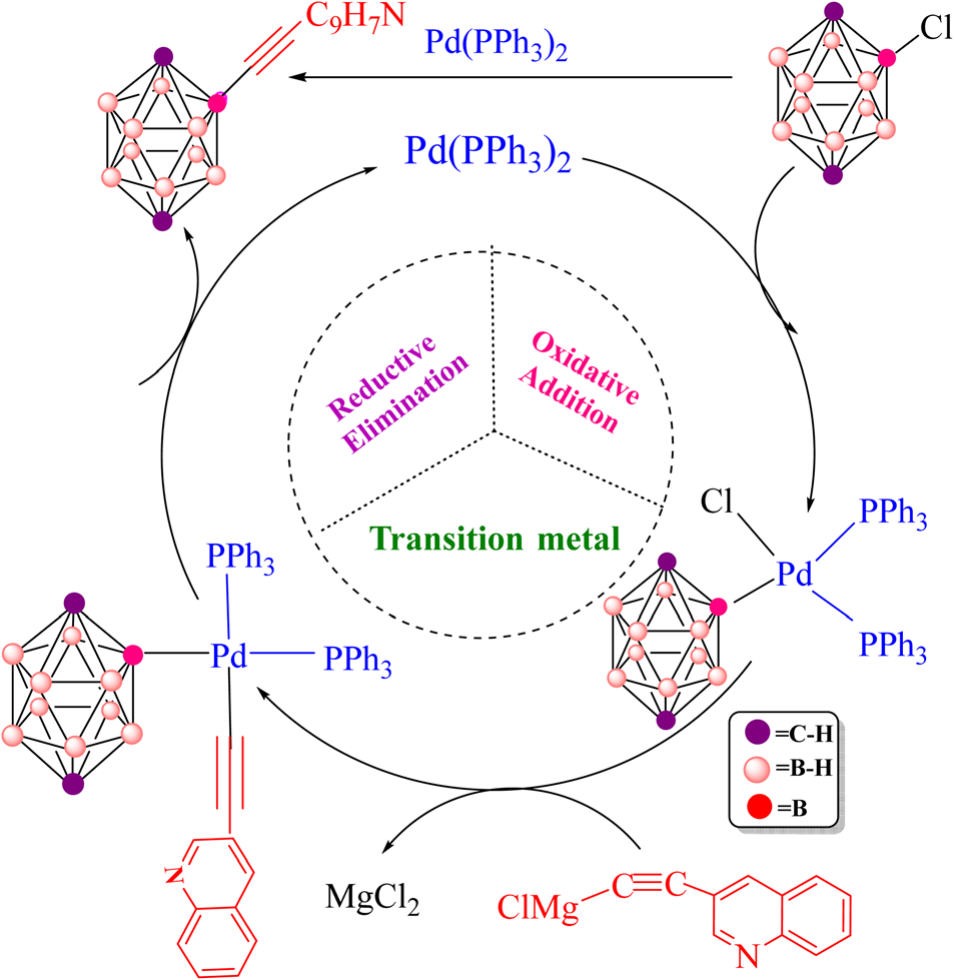
Pd‑catalyzed Kumada–Tamao–Corriu (Kumada) cross‑coupling of a representative 2‑Cl‑*p*‑carborane. OA = oxidative addition, TM = transmetalation, RE = reductive elimination.

**Fig. 13:**
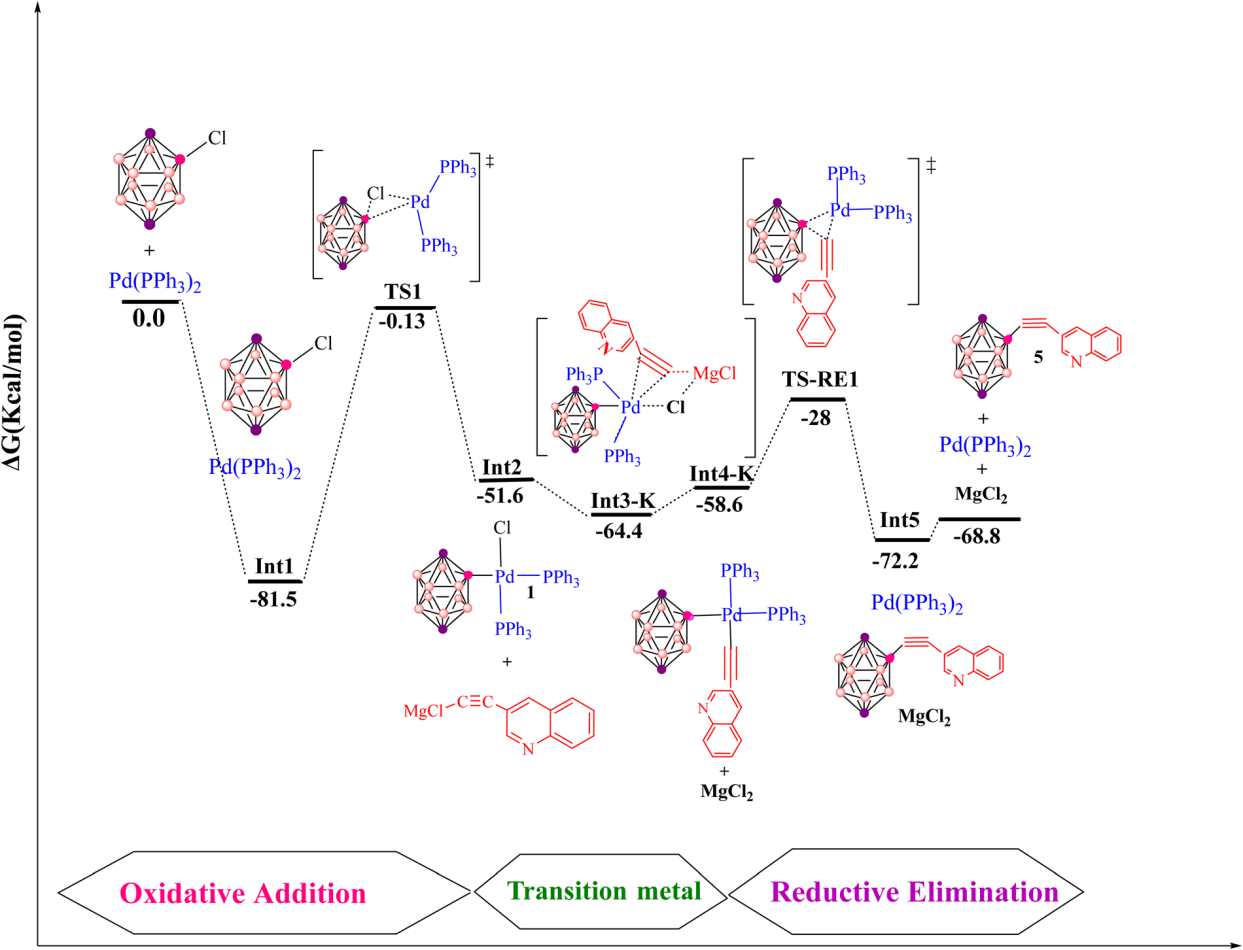
Gibbs free‑energy diagram for the Pd‑catalyzed Kumada mechanism, computed at the B3LYP‑D3/def2‑TZVPP//B3LYP‑D3/BSL level in 1,4‑dioxane.

The Kumada cross-coupling reaction proceeds through three fundamental steps: oxidative addition, transmetalation and reductive elimination (Fig. 12). In the present study, the Pd(PPh_3_)_2_Cl_2_ complex is employed as the catalyst for B–C bond formation in carborane substrates, and the mechanism is analysed using 3-quinolylethynyl magnesium chloride and 2-Cl-*p*-carborane as coupling partners in 1,4-dioxane (Scheme 4). This section assesses the computational feasibility of the Kumada *versus* Sonogashira cross-coupling pathways for the synthesis of product 5. As shown in Fig. 13, the oxidative addition step is common to both mechanisms, after which the pathways diverge. The computed Kumada manifold highlights an overall efficient route to 3-quinolylethynyl carborane. The sequence begins with formation of intermediate Int2 from the separated reactants *via* a three-centre transition state TS1 at −0.13 kcal mol^−1^, affording Int2 at −51.6 kcal mol^−1^. We performed additional calculations to evaluate the effect of magnesium speciation on the key intermediate Int3-k. In particular, Int3 was modeled using both monomeric MgCl_2_ and a representative dimeric MgCl_2_ species, which serves as a simple model for aggregated magnesium structures commonly present in Grignard equilibria. The results show (Table S1) that the monomeric Int3 structure is 9.34 kcal mol^−1^ more stable than the corresponding dimeric model relative to Int2. Importantly, although the absolute stabilization differs slightly depending on the aggregation state, the qualitative energetic picture of the Kumada pathway remains unchanged. This transformation involves migration of MgCl_2_ from Int3-K to the borate moiety, promoting cleavage of the Pd–Cl bond and formation of a Pd–C bond; at this stage, intermediate Int3-K is located at −64.4 kcal mol^−1^, with the aryl fragment coordinated to both palladium and magnesium. In the final phase of the catalytic cycle, B–C bond formation occurs *via* reductive elimination from Int5, which lies at −72.2 kcal mol^−1^. This step proceeds through transition state RE1-TS at −28.0 kcal mol^−1^ and requires an activation barrier of approximately 30.6 kcal mol^−1^ relative to Int4. The value of 30.6 kcal mol^−1^ corresponds to the intrinsic reductive-elimination (RE) barrier in the Kumada pathway, defined as the free-energy difference between the post-transmetalation intermediate (Int4-K) and the corresponding transition state (TS-RE1). The catalytic cycle is completed by release of the cross-coupled product and regeneration of Pd(PPh_3_)_2_ at −68.8 kcal mol^−1^. Comparison of this profile with the copper-free Sonogashira pathways (Sections 3.2–3.4) provides a quantitative basis for evaluating the relative kinetic and thermodynamic viability of Kumada *versus* Sonogashira B–C bond-forming strategies in carborane chemistry. Overall, the key B–C bond-forming reductive elimination thus proceeds with a substantially higher barrier in the Kumada manifold (30.6 kcal mol^−1^ from Int4) than in the anionic copper-free Sonogashira pathway, and delivers a less stable product (Δ*G* = −68.8 *vs.* −80.7 kcal mol^−1^), underscoring the kinetic and thermodynamic preference for the Sonogashira route.

### 3.6. Which route is more favorable for 3-quinolylethynyl carborane: Sonogashira or Kumada?

**Scheme 5:**
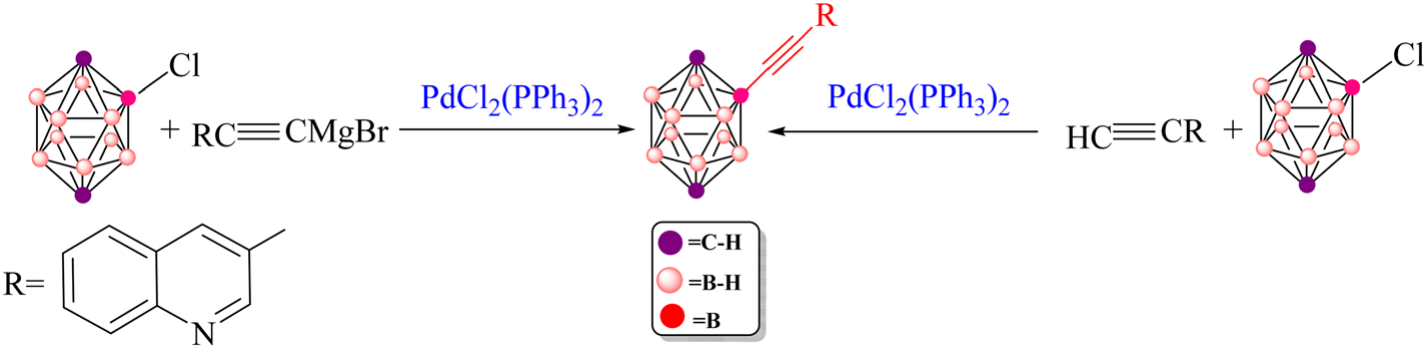
Preparation of 3-quinolylethynyl carborane *via* copper-free Sonogashira and Kumada cross coupling reactions.

In 2004, Irina P. Beletskaya and co-workers reported the synthesis of 3-quinolylethynyl carborane *via* both Sonogashira and Kumada cross-coupling reactions (Scheme 5).^116^ They employed 3-quinolyl–C≡C–X (X = H or MgCl) as the coupling partner. Under standard Sonogashira conditions using Pd(PPh_3_)_2_Cl_2_ as the catalyst, pyrrolidine as the base and 1,4-dioxane as the solvent, the 3-quinolylalkyne product was obtained in 80% yield, underscoring the synthetic efficiency of this route. Theoretical calculations carried out in this work further support the superiority of the Sonogashira pathway over the Kumada alternative for constructing the B–C bond between 3-quinolyl–C≡C–X and 2-Cl-*p*-carborane. In particular, the activation barrier for the key reductive-elimination transition state TS-RE1 in the Kumada mechanism is significantly higher (22.7 kcal mol^−1^) than that of the corresponding anionic Sonogashira pathway, indicating a kinetic preference for the latter. In particular, the relative free-energy difference between the key reductive-elimination transition state (TS-RE1) of the Kumada mechanism and the corresponding transition state in the anionic Sonogashira pathway is calculated to be 22.7 kcal mol^−1^. This value reflects the energy separation between the two competing pathways when referenced to a common starting point, as illustrated in the comparative energy profiles. Accordingly, it serves as a measure for cross-pathway comparison and should not be interpreted as an intrinsic activation barrier for any individual elementary step. The lower transition-state energy associated with the anionic pathway is consistent with its preferred reactivity under the studied conditions. In addition, comparison of product stabilities shows that the Sonogashira product (−80.7 kcal mol^−1^) is more stable than the Kumada product (−68.8 kcal mol^−1^), demonstrating that thermodynamic factors also favour the Sonogashira route. Taken together, these kinetic and thermodynamic data indicate that, when Pd(PPh_3_)_2_ is used as the catalyst, the anionic copper-free Sonogashira pathway is the most advantageous strategy for synthesizing 3-quinolylethynyl carborane. The combination of experimental observations and DFT results thus highlights the efficiency and reliability of this synthetic approach. Fig. S1–S6 and S8 provide a comprehensive overview of the optimized structures, bond lengths (Å) and Wiberg bond indices (WBIs) for the transition states involved in the various Sonogashira and Kumada manifolds considered. Specifically, Fig. S1 summarizes the common structural motifs for the standard Sonogashira reaction, Fig. S2 addresses the copper-free carbopalladation variant, Fig. S3 details the cationic copper-free Sonogashira pathway, Fig. S4 and S5 depict key intermediates for the anionic and ionic copper-free mechanisms, and Fig. S6 presents the optimized structures for the Kumada cross-coupling reaction. All calculations were performed at the B3LYP-D3/BSL level with 1,4-dioxane as the solvent.

## 4. Conclusion

The growing body of work on carborane chemistry underscores the importance of these boron-rich clusters in both fundamental studies and applied research, particularly in medicinal chemistry. Their rigid three-dimensional architectures and distinctive reactivity profiles make them attractive building blocks for the design of new therapeutic agents and functional materials. Metal-catalyzed cross-coupling reactions are central to the functionalization of carboranes, and this study has focused on Pd-catalyzed B–C bond formation through copper-free Sonogashira and Kumada manifolds. DFT calculations at the B3LYP-D3 level were used to analyse the copper-free Sonogashira coupling between 3-ethynylquinoline and 2-Cl-*p*-carborane *via* three mechanistic scenarios: carbopalladation, deprotonation (cationic and anionic) and an ionic pathway. The carbopalladation route exhibits a prohibitively high activation barrier and can therefore be ruled out under standard conditions. By contrast, the cationic and anionic deprotonation pathways, as well as the ionic variant in which the base initially replaces the halide at palladium, all display substantially lower Gibbs energy barriers and are mechanistically viable. Among these, the anionic pathway emerges as both thermodynamically and kinetically most favourable for the present system. The comparison between copper-free Sonogashira and Pd-catalyzed Kumada cross-coupling is particularly informative in the context of 3-quinolylethynyl carborane synthesis. The calculations show that the key reductive-elimination barrier in the Kumada manifold is higher and that the resulting product is less stable than in the corresponding anionic Sonogashira pathway, indicating that both kinetic and thermodynamic factors favour the latter. In line with the experimental study by Beletskaya and co-workers, the combined computational and experimental evidence supports copper-free Sonogashira coupling with Pd(PPh_3_)_2_ as the most advantageous strategy for accessing 3-quinolylethynyl carborane under the conditions examined. Overall, this work illustrates how detailed mechanistic analysis can guide the selection and optimization of cross-coupling conditions for carborane-based targets. The results highlight that relatively subtle changes in base, ligands, substrates or solvent can shift the balance between competing carbopalladation, deprotonation and ionic pathways, and that, for the system studied here, the anionic copper-free Sonogashira route provides the most favourable B–C bond-forming platform for 3-quinolylethynyl carborane.

## Conflicts of interest

The authors have declared no conflict of interest.

## Supplementary Material

RA-016-D5RA09733A-s001

## Data Availability

The data supporting this article have been included as part of the supplementary information (SI). Supplementary information is available. See DOI: https://doi.org/10.1039/d5ra09733a.
