# Supplementary material for: Deprotonation-controlled copper-free Pd-catalyzed Sonogashira coupling versus the Kumada–Tamao–Corriu reaction: a DFT investigation toward anticancer carborane alkynes
Source: RSC Adv. 2026 Apr 21;16(23):20822–39. doi: 10.1039/d5ra09733a (PMC13098324; doi:10.1039/d5ra09733a)
Supplement: RA-016-D5RA09733A-s001 [file RA-016-D5RA09733A-s001.pdf]

Supplementary Information (SI) for RSC Advances.
This journal is © The Royal Society of Chemistry 2026

**SUPPORTING INFORMATION**

**for**

# Deprotonation-Controlled Copper-Free Pd-Catalyzed Sonogashira Coupling versus Kumada–Tamao–Corriu Reaction: A DFT Investigation toward Anticancer Carborane Alkynes

Elham Soltani [a], Mehdi Bayat* [b]

[a] Department of Inorganic Chemistry, Faculty of Chemistry, Bu-Ali Sina University, Hamedan 65178-38683, Iran

[b] School of Chemistry, College of Science, University of Tehran, Tehran, Iran

Email: bayatm@ut.ac.ir (mehdi806@gmail.com)

**Title**

**Figure S1.** Optimized structures of reactants, transition states, and intermediates for the standard Sonogashira reaction (showing the common structures) involved in the synthesis of 3-quinolylethynyl carborane. All structures were calculated at the B3LYP-D3/BSL level of theory with 1,4-dioxane as the solvent.

**Figure S2.** Optimized structures of reactants, transition states, and intermediates for the copper-free carbopalladation mechanism of the Sonogashira reaction in the synthesis of 3-quinolylethynyl carborane, calculated at the B3LYP-D3/BSL level of theory with 1,4-dioxane as the solvent.

**Figure S3.** Optimized structures of reactants, transition states, and intermediates for the cationic mechanism of the Sonogashira reaction for the synthesis of 3-quinolylethynyl carborane, calculated at the B3LYP-D3/BSL level of theory with 1,4-dioxane as the solvent.

---

*Corresponding author. e-mail: mbayat@basu.ac.ir(mehdi806@gmail.com)

† Electronic Supplementary Information (ESI) available: See DOI:

**Figure S4.** Optimized structures of reactants, transition states, and intermediates for the anionic mechanism of the Sonogashira reaction in the synthesis of 3-quinolylethynyl carborane, calculated at the B3LYP-D3/BSL level of theory with 1,4-dioxane as the solvent.

**Figure S5.** Optimized structures of reactants, transition states, and intermediates for the ionic mechanism of the Sonogashira reaction for the synthesis of 3-quinolylethynyl carborane, calculated at the B3LYP-D3/BSL level of theory with 1,4-dioxane as the solvent.

**Figure S6.** Optimized structures of reactants, transition states, and intermediates for the Kumada reaction in the synthesis of 3-quinolylethynyl carborane, calculated at the B3LYP-D3/BSL level of theory with 1,4-dioxane as the solvent.

**Figure S7**. Optimized geometries of the transition states for 3-quinolylethynyl carborane synthesis, along with key bond lengths and Wiberg bond indices. All calculations were performed at the B3LYP-D3/BSL level of theory in 1,4-dioxane solvent. Bond lengths are reported in Angstroms (Å).

**Figure S8.** Some of the intrinsic reaction coordinate (IRC) analyses for the transition states involved in the formation of 3-quinolylethynyl carborane via Sonogashira and Kumada coupling reactions. All calculations were performed at the B3LYP-D3/BSL level of theory in 1,4-dioxane solvent.

**Table S1.** The resulting energies are Included the Monomeric $MgCl_2$ (Int3-K) vs Dimeric $MgCl_2$ (Int3-K)comparison for the Kumada reaction in the synthesis of 3-quinolylethynyl carborane, calculated at the B3LYP-D3/BSL level of theory with 1,4-dioxane as the solvent.

**Table S2.** The resulting energies are Included the Int3-K, Int4-K, TS-RE1 and Int5 comparison for the Kumada reaction in the synthesis of 3-quinolylethynyl carborane, calculated at the B3LYP-D3/BSL and CAM-B3LYP/BSL level of theory with 1,4-dioxane as the solvent.

Figure S1. Optimized structures of reactants, transition states, and intermediates for the standard Sonogashira reaction (showing the common structures) involved in the synthesis of 3-quinolylethynyl carborane. All structures were calculated at the B3LYP-D3/BSL level of theory with 1,4-dioxane as the solvent.

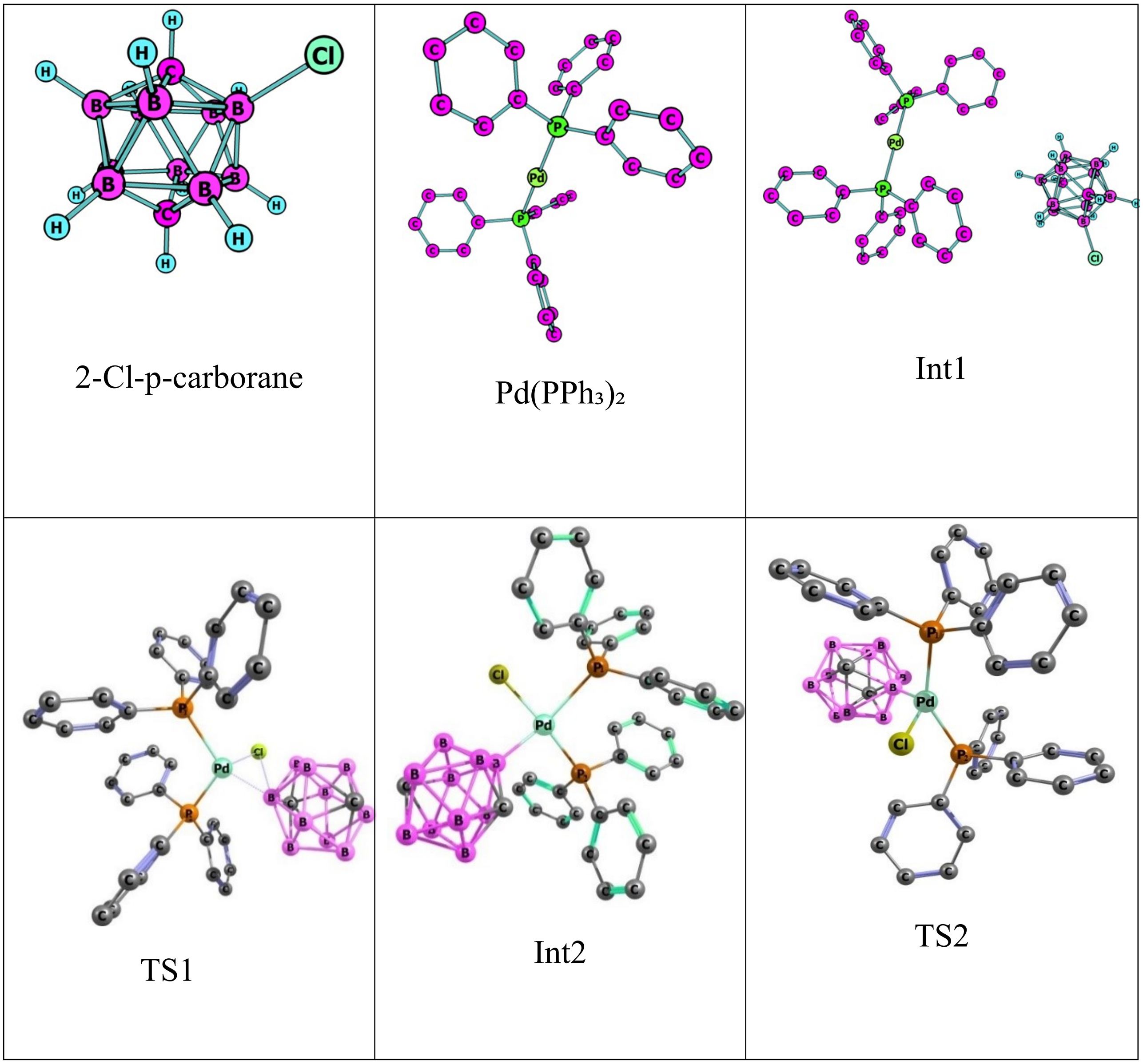

| | | |
|---|---|---|
| 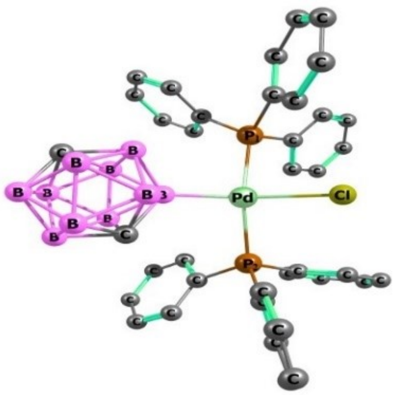<br>Int3 | 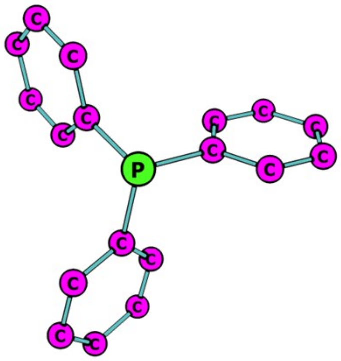<br>PPh3 | 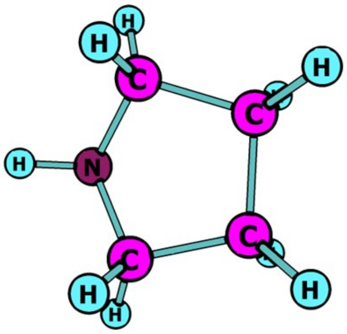<br>Pyrrolidine |
| 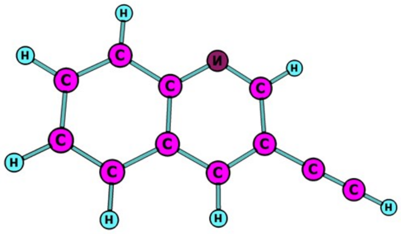<br>3-ethynyl quinoline | 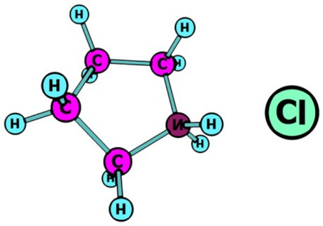<br>H-Base$^{+}$ Cl$^{-}$ | 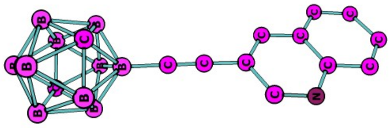<br>3-quinolylethynyl carborane |

Figure S2. Optimized structures of reactants, transition states, and intermediates for the copper-free carbopalladation mechanism of the Sonogashira reaction in the synthesis of 3-quinolylethynyl carborane, calculated at the B3LYP-D3/BSL level of theory with 1,4-dioxane as the solvent.

| | | |
|---|---|---|
| 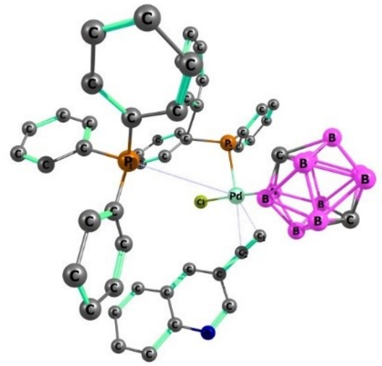 Ts3 | 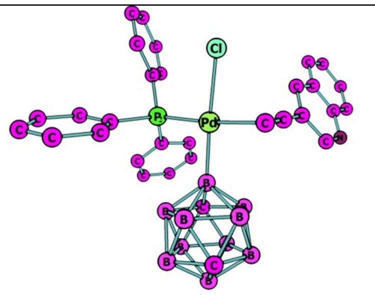 Int4 | 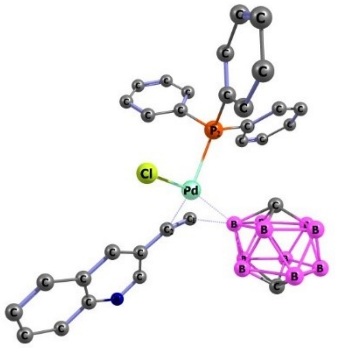 TS1-C |
| 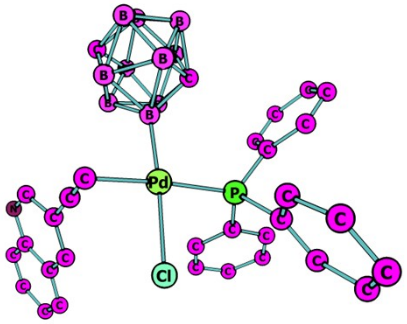 Int1-C | 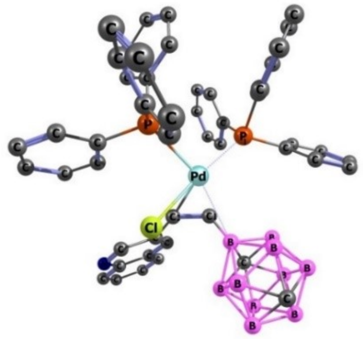 TS2-C | 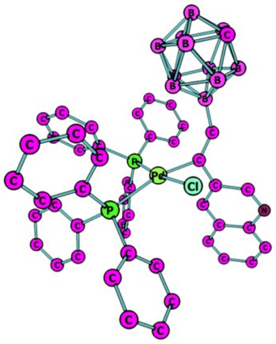 Int1-RE-C |
| 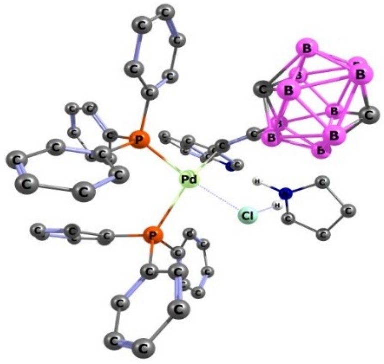 TS1-RE-C | 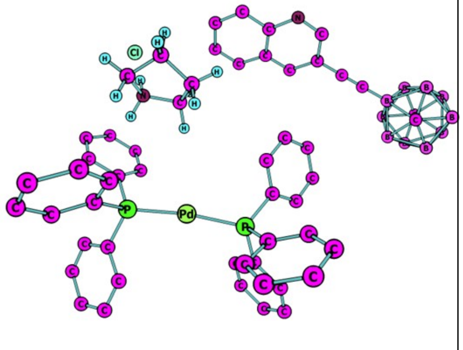 Int2-RE-C | |

Figure S3. Optimized structures of reactants, transition states, and intermediates for the cationic mechanism of the Sonogashira reaction for the synthesis of 3-quinolylethynyl carborane, calculated at the B3LYP-D3/BSL level of theory with 1,4-dioxane as the solvent.

| | | |
|---|---|---|
| 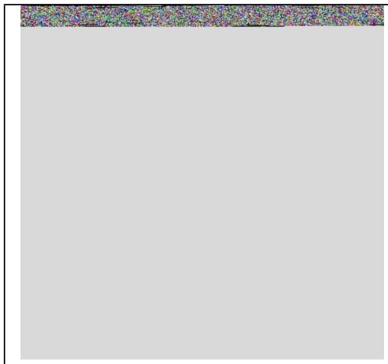 TS3 | 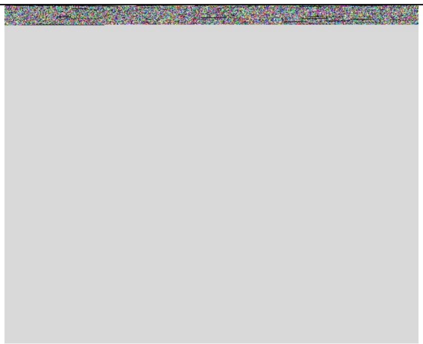 Int4 | 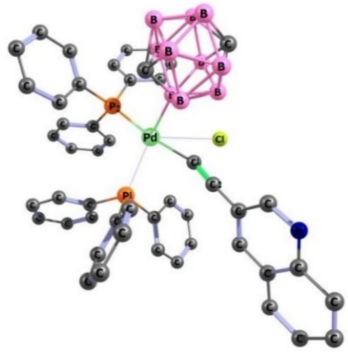 TS1-DC |
| 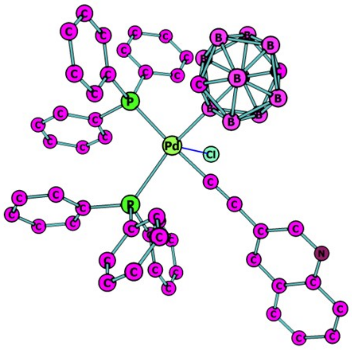 Int1-DC | 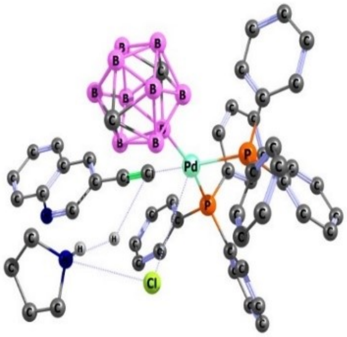 TS2-DC | 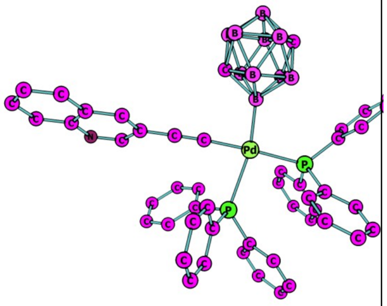 Int1-RE |
| 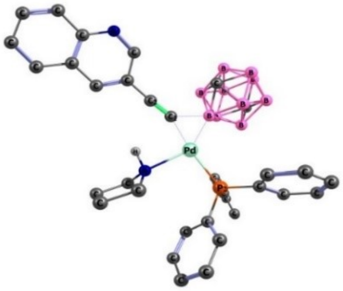 TS-RE1 | 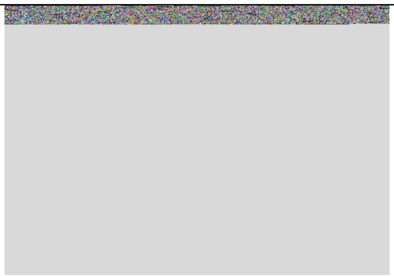 Int2-RE | |

Figure S4. Optimized structures of reactants, transition states, and intermediates for the anionic mechanism of the Sonogashira reaction in the synthesis of 3-quinolylethynyl carborane, calculated at the B3LYP-D3/BSL level of theory with 1,4-dioxane as the solvent.

| | | |
|---|---|---|
| 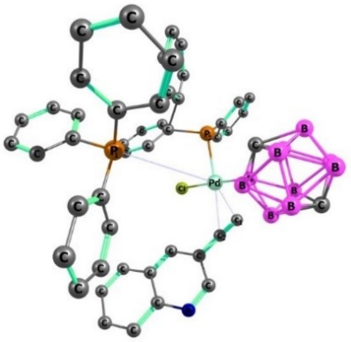 TS3 | 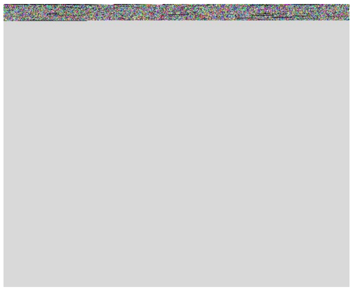 Int4 | 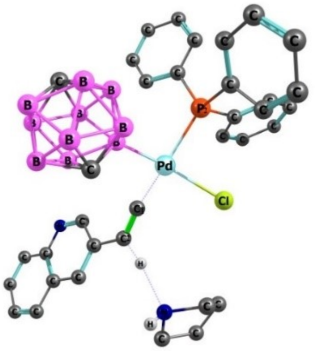 TS1-DA |
| 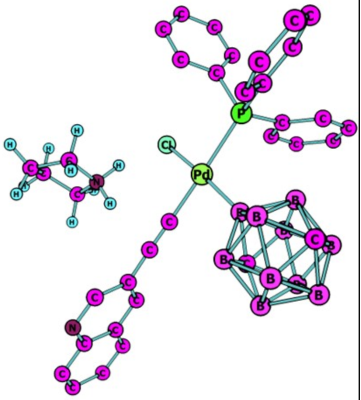 Int1-DA | 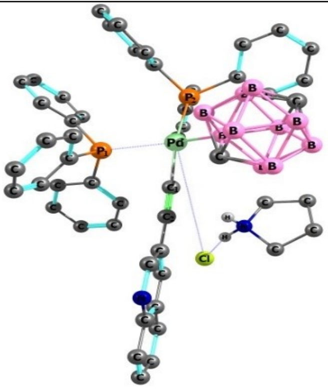 TS2-DA | 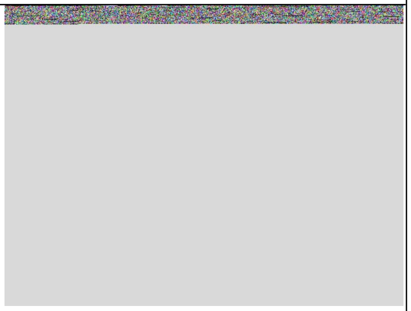 Int1-RE |
| 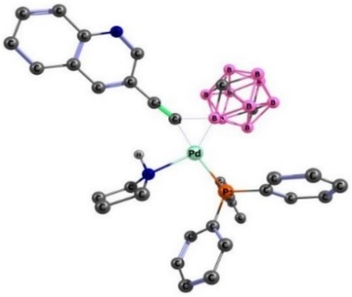 TS-RE1 | 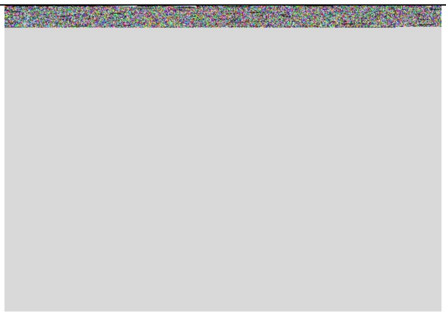 Int2-RE | |

Figure S5. Optimized structures of reactants, transition states, and intermediates for the ionic mechanism of the Sonogashira reaction for the synthesis of 3-quinolylethynyl carborane, calculated at the B3LYP-D3/BSL level of theory with 1,4-dioxane as the solvent.

| | | |
|---|---|---|
| 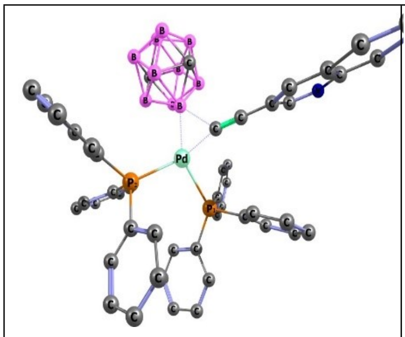 TS1-I | 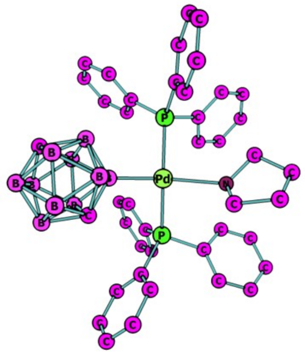 Int1-I | 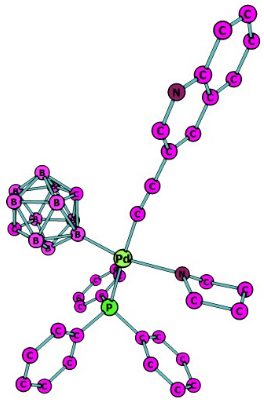 Int1-RE2 |
| 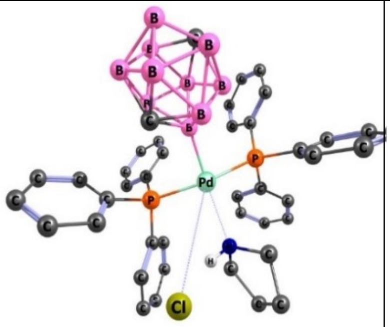 TS1-RE2 | 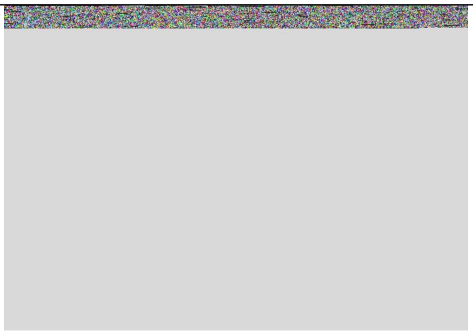 Int2-RE2 | |

Figure S6. Optimized structures of reactants, transition states, and intermediates for the Kumada reaction in the synthesis of 3-quinolylethynyl carborane, calculated at the B3LYP-D3/BSL level of theory with 1,4-dioxane as the solvent.

| | | |
|---|---|---|
| 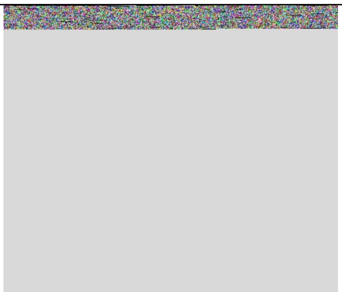<br>2-Cl-p-carborane | 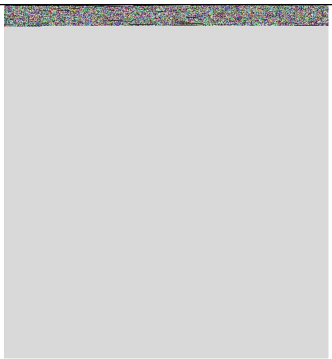<br>$Pd(PPh_3)_2$ | 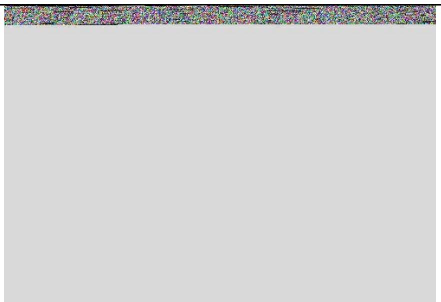<br>Int1 |
| 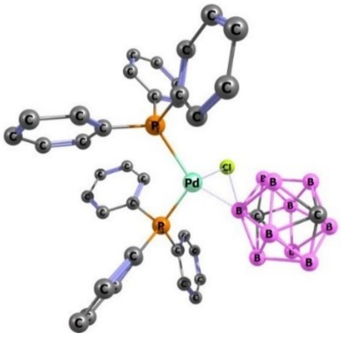<br>TS1 | 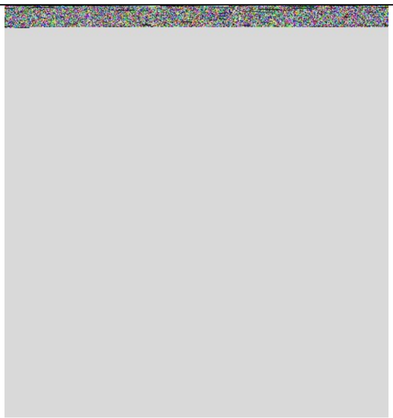<br>Int2 | 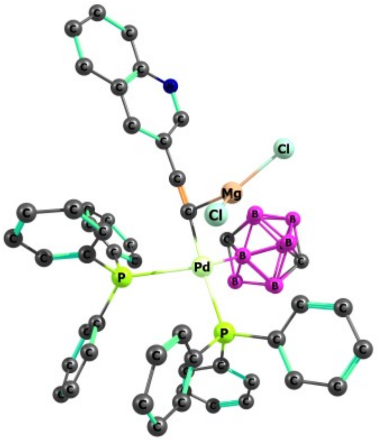<br>Int3-k |

| | | |
|---|---|---|
| 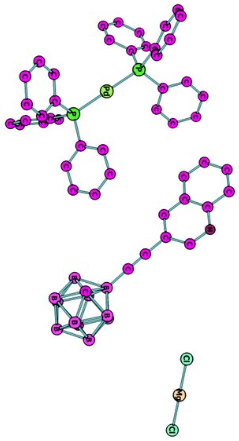<br>Int5 | 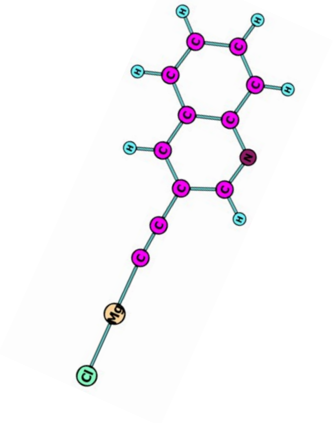<br>3-quinolylethynyl magnesium bromide | 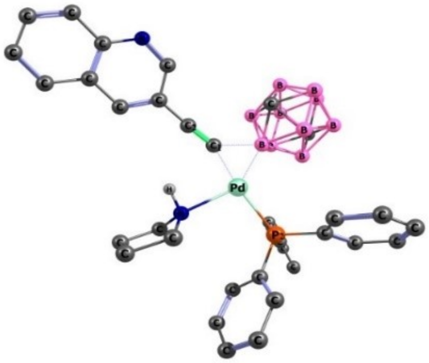<br>TS-RE1 |
| 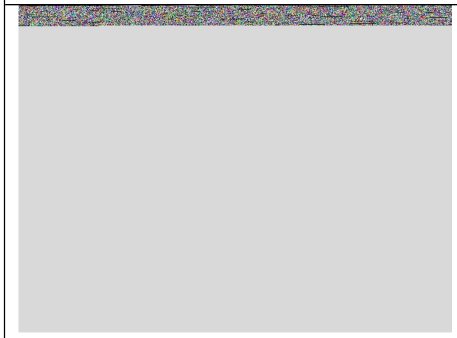<br>Int4-K | 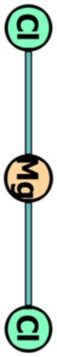<br>MgCl2 | 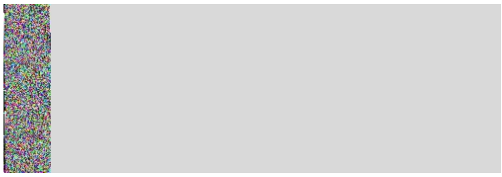<br>3-quinolylethynyl carborane |

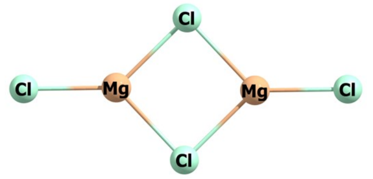


$Mg_2Cl_4$

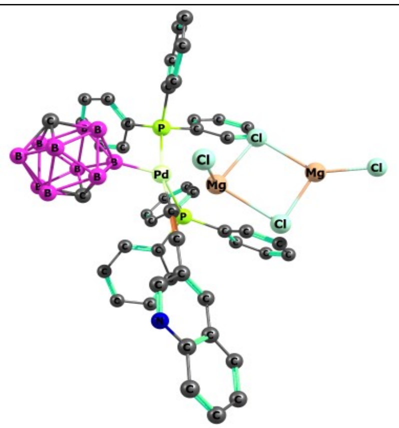

Dimeric $MgCl_2$ (Int3-K)

Figure S7. Optimized geometries of the transition states for 3-quinolylethynyl carborane synthesis, along with key bond lengths and Wiberg bond indices. All calculations were performed at the B3LYP-D3/BSL level of theory in 1,4-dioxane solvent. Bond lengths are reported in Angstroms (Å).

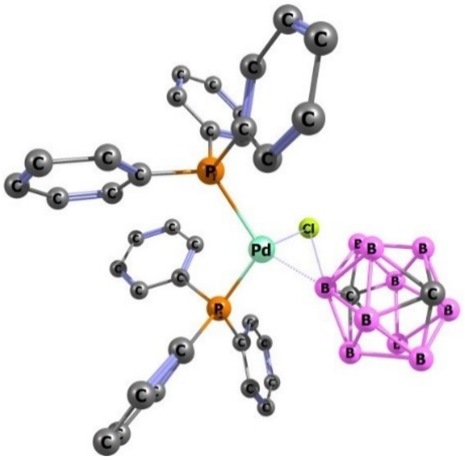

**TS1**

**Pd-Cl: 3.1(0.14)**
**Pd-B:2.0(0.56)**
**B-Cl: 3.0(0.09)**

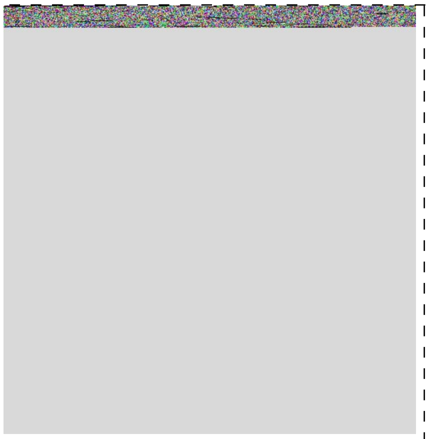

**TS2**

**Pd-P1:2.3(0.46)**
**Pd-P2:2.5(0.39)**

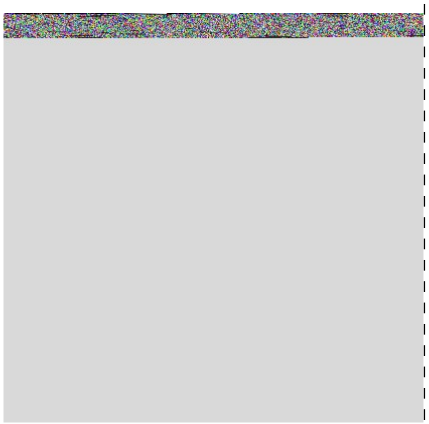

**TS3**

**Pd-P1:6.2(0.004)**
**Pd-P2:2.4(0.48)**
**Pd-C10:2.55(0.13)**
**Pd-C11: 2.24(0.22)**

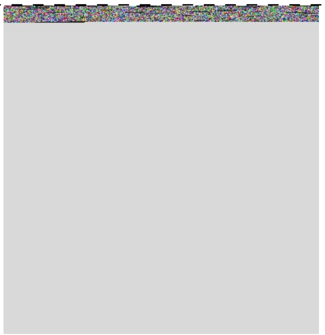

**TS1**-C

**Pd-B: 2.2(0.43)**
**Pd-C10:2.1(0.51)**
**B-C11: 2.1(0.47)**
**C10-C11:1.29(1.14)**

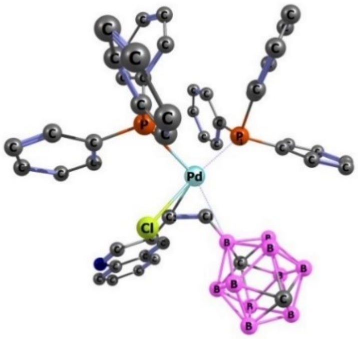

**TS2-C**

**Pd-P1:2.43(0.45)**
**Pd-B:3.7(0.02)**
**B-C29:1.58(0.92)**

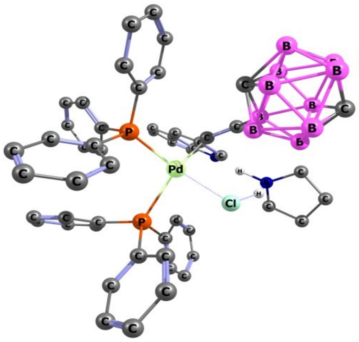

**TS1-RE-C**

**Pd-Cl:2.53(0.39)**
**Pd-C28:2.04(0.57)**
**Cl-H48:1.96(1.0)**

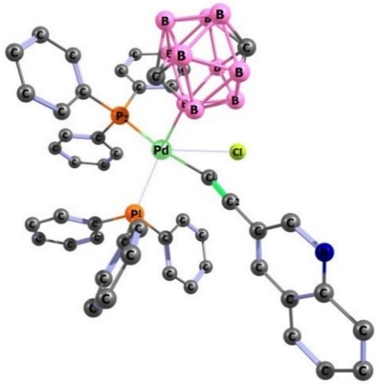

## TS1-DC

**Pd-Cl:3.73(0.08)**
**Pd-P1:2.68(0.42)**

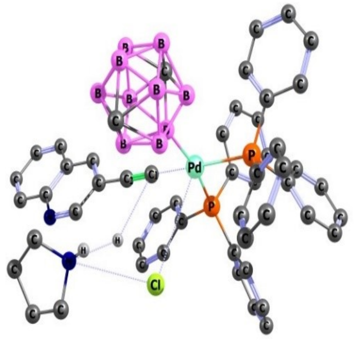

## TS2-DC

**Pd-Cl:4.1(0.05)**
**Cl-H48:3.3(0.07)**
**N2-H57:2.77(0.12)**
**Pd-C30:3.2(0.05)**
**Pd-C31:1.98(0.57)**

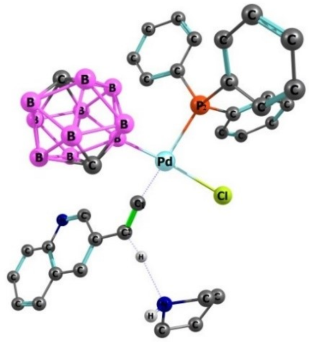

## TS1-DA

**Pd-C31:1.90(0.78)**
**Pd-B:2.12(0.58)**
**Pd-C30:3.18(0.05)**
**H42-N2:1.87(0.9)**

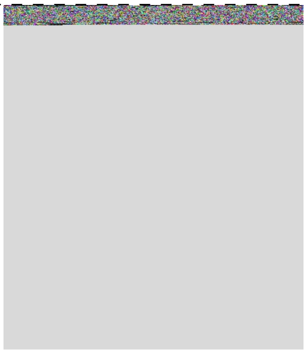

## TS2-DA

**Pd-Cl:6.3(0.00)**
**Pd-P2:2.69(0.2)**
**Cl-H57:1.94(0.28)**

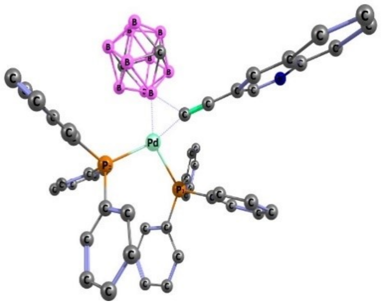

## TS1-I

**Pd-Cl:4.34(0.09)**
**Pd-N1:2.39(0.15)**

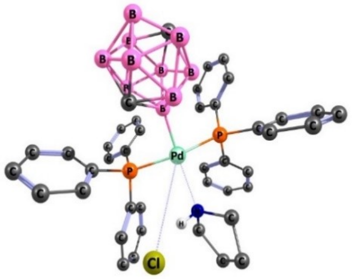

## TS1-RE2

**Pd-B:2.21(0.43)**
**B-C31:2.04(0.2)**
**Pd-C31:1.96(0.44)**

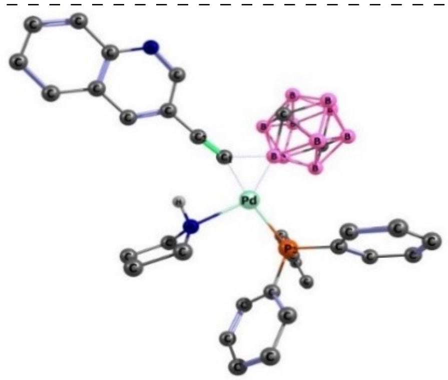

**TS-RE1**

Pd-B:2.2(0.4)
Pd-C47:1.96(0.5)
B-C47:2.0(0.43)

Figure S8. Some of the intrinsic reaction coordinate (IRC) analyses for the transition states involved in the formation of 3-quinolylethynyl carborane via Sonogashira and Kumada coupling reactions. All calculations were performed at the B3LYP-D3/BSL level of theory in 1,4-dioxane solvent.

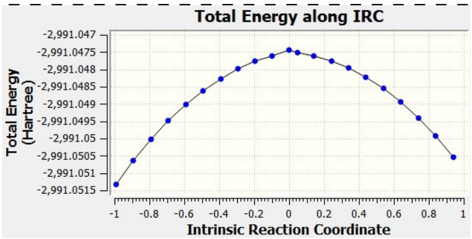


**IRC TS1**

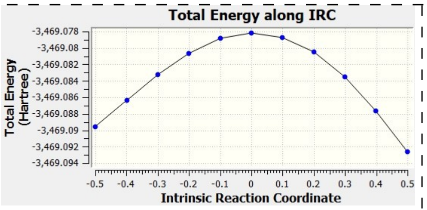


**IRC TS3**

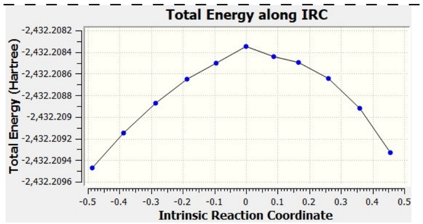


**IRC TS1-C**

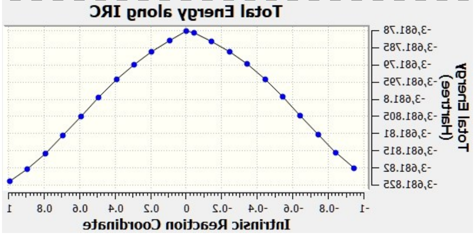

**IRC TS1-RE-C**

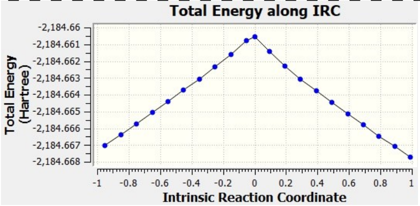


**IRC TS1-RE2**

| | E(Hartree) | ΔE = E(Int3-K) – E(Int2) (kcal $mol^{-1}$) |
|---|---|---|
| Monomeric $MgCl_2$ (Int3-K) | -4130.372336 | -64.43 |
| Dimeric $MgCl_2$ (Int3-K) | -5251.162856 | -55.09 |

**Table S1.** The resulting energies are Included the Monomeric $MgCl_2$ (Int3-K) vs Dimeric $MgCl_2$ (Int3-K) comparison for the Kumada reaction in the synthesis of 3-quinolylethynyl carborane, calculated at the B3LYP-D3/BSL level of theory with 1,4-dioxane as the solvent.

| | ΔE (kcal $mol^{-1}$) B3LYP-D3 | ΔE (kcal $mol^{-1}$) Cam- B3LYP |
|---|---|---|
| Int3-K | -64.43 | -54.6 |
| Int4-K | -58.6 | -49.3 |
| TS-RE1 | -28 | -16.3 |
| Int5 | -72.2 | -71 |

**Table S2.** The resulting energies are Included the Int3-K, Int4-K, TS-RE1 and Int5 comparison for the Kumada reaction in the synthesis of 3-quinolylethynyl carborane, calculated at the B3LYP-D3/BSL and CAM-B3LYP/BSL level of theory with 1,4-dioxane as the solvent.

2-Cl-p-carborane

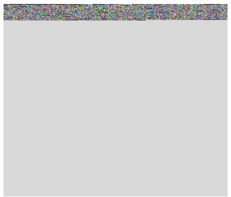

| Atom | x | y | z |
|---|---|---|---|
| H | 1.753374000 | 1.483016000 | -2.188026000 |
| H | 1.753372000 | -1.483144000 | -2.187940000 |
| H | -0.635217000 | -0.000066000 | -2.288894000 |
| H | -0.727477000 | -2.391334000 | -0.829343000 |
| B | 1.346567000 | 0.893207000 | -1.245850000 |
| B | 1.346567000 | -0.893280000 | -1.245798000 |
| C | -0.106255000 | -0.000039000 | -1.342003000 |
| B | -0.137187000 | -1.447122000 | -0.429780000 |
| B | 1.400050000 | 1.444472000 | 0.432303000 |
| H | 1.972737000 | 2.398081000 | 0.836951000 |
| B | 2.318117000 | -0.000003000 | -0.070539000 |
| H | 3.499354000 | 0.000000000 | 0.003010000 |
| C | 1.357850000 | 0.000038000 | 1.342806000 |
| H | 1.875605000 | 0.000066000 | 2.295538000 |
| B | -0.137186000 | 1.447096000 | -0.429864000 |
| H | -0.727475000 | 2.391286000 | -0.829481000 |
| B | -0.089360000 | 0.895786000 | 1.248870000 |
| H | -0.510495000 | 1.480719000 | 2.187065000 |
| B | 1.400048000 | -1.444446000 | 0.432387000 |
| H | 1.972733000 | -2.398033000 | 0.837092000 |
| B | -0.089361000 | -0.895712000 | 1.248922000 |
| H | -0.510496000 | -1.480591000 | 2.187151000 |

| | | | |
|---|---|---|---|
| B | -1.064956000 | 0.000003000 | 0.074213000 |
| Cl | -2.864240000 | 0.000000000 | -0.006015000 |

## $Pd(PPh_3)_2$

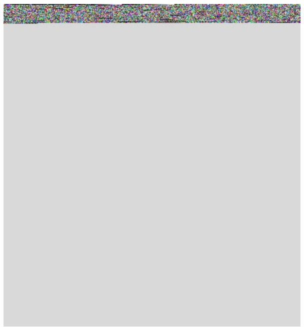

| Atom | x | y | z |
|---|---|---|---|
| Pd | 0.000073000 | 0.007461000 | 0.012440000 |
| P | -2.328074000 | 0.002844000 | 0.004040000 |
| P | 2.327948000 | 0.002205000 | 0.004007000 |
| C | -3.100097000 | -1.604767000 | -0.492127000 |
| C | -2.431191000 | -2.784395000 | -0.124461000 |
| H | -1.485130000 | -2.713515000 | 0.407141000 |
| C | -2.960725000 | -4.033681000 | -0.449135000 |
| H | -2.431255000 | -4.936549000 | -0.156476000 |
| C | -4.159730000 | -4.122389000 | -1.161298000 |
| H | -4.568191000 | -5.094799000 | -1.423422000 |
| C | -4.825645000 | -2.956445000 | -1.544220000 |
| H | -5.754495000 | -3.018300000 | -2.105388000 |
| C | -4.301214000 | -1.704767000 | -1.211077000 |
| H | -4.826978000 | -0.805928000 | -1.518902000 |
| C | -3.124936000 | 0.375258000 | 1.633085000 |

| | | | |
|---|---|---|---|
| C | -4.329717000 | -0.202634000 | 2.062188000 |
| H | -4.842971000 | -0.921612000 | 1.430723000 |
| C | -4.874110000 | 0.133296000 | 3.304545000 |
| H | -5.805630000 | -0.325883000 | 3.625378000 |
| C | -4.224664000 | 1.051730000 | 4.131673000 |
| H | -4.648511000 | 1.309755000 | 5.098585000 |
| C | -3.022356000 | 1.629684000 | 3.715628000 |
| H | -2.505748000 | 2.337772000 | 4.358167000 |
| C | -2.473101000 | 1.287694000 | 2.479566000 |
| H | -1.525071000 | 1.718045000 | 2.165655000 |
| C | -3.109836000 | 1.227390000 | -1.143124000 |
| C | -2.431712000 | 1.520417000 | -2.338245000 |
| H | -1.473552000 | 1.044777000 | -2.534112000 |
| C | -2.968205000 | 2.421594000 | -3.258503000 |
| H | -2.431411000 | 2.636644000 | -4.178702000 |
| C | -4.183848000 | 3.056148000 | -2.990094000 |
| H | -4.597921000 | 3.765770000 | -3.701513000 |
| C | -4.859670000 | 2.782893000 | -1.799194000 |
| H | -5.801777000 | 3.279044000 | -1.580635000 |
| C | -4.328006000 | 1.873356000 | -0.881275000 |
| H | -4.861364000 | 1.673515000 | 0.043273000 |
| C | 3.103074000 | -1.387311000 | -0.942025000 |
| C | 2.426650000 | -1.851046000 | -2.082902000 |
| H | 1.473582000 | -1.402398000 | -2.353027000 |
| C | 2.958207000 | -2.884907000 | -2.854386000 |
| H | 2.422901000 | -3.231307000 | -3.734501000 |
| C | 4.166911000 | -3.482350000 | -2.487347000 |
| H | 4.576971000 | -4.294528000 | -3.081774000 |

| | | | |
|---|---|---|---|
| C | 4.840645000 | -3.038925000 | -1.347619000 |
| H | 5.777173000 | -3.504733000 | -1.051949000 |
| C | 4.314066000 | -1.997035000 | -0.579695000 |
| H | 4.845904000 | -1.665222000 | 0.306959000 |
| C | 3.111401000 | 1.510727000 | -0.729100000 |
| C | 4.314492000 | 1.492547000 | -1.451391000 |
| H | 4.835615000 | 0.554254000 | -1.615826000 |
| C | 4.847429000 | 2.675414000 | -1.970662000 |
| H | 5.777738000 | 2.645072000 | -2.531998000 |
| C | 4.188156000 | 3.890120000 | -1.772680000 |
| H | 4.603105000 | 4.808464000 | -2.179587000 |
| C | 2.987316000 | 3.918503000 | -1.058525000 |
| H | 2.462955000 | 4.858622000 | -0.908937000 |
| C | 2.449306000 | 2.736717000 | -0.548176000 |
| H | 1.501663000 | 2.753899000 | -0.014937000 |
| C | 3.120315000 | -0.121668000 | 1.672680000 |
| C | 4.331277000 | 0.503465000 | 2.008649000 |
| H | 4.851668000 | 1.110211000 | 1.273728000 |
| C | 4.872227000 | 0.359405000 | 3.289087000 |
| H | 5.808276000 | 0.853519000 | 3.536282000 |
| C | 4.213125000 | -0.412437000 | 4.247964000 |
| H | 4.633903000 | -0.520734000 | 5.244067000 |
| C | 3.004936000 | -1.035792000 | 3.924919000 |
| H | 2.481123000 | -1.630039000 | 4.668962000 |
| C | 2.459119000 | -0.884795000 | 2.649752000 |
| H | 1.506616000 | -1.348864000 | 2.404959000 |

Int1

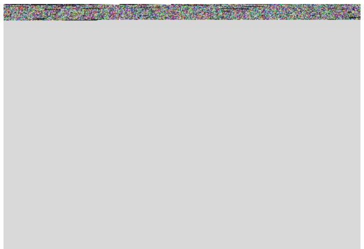

| Atom | x | y | z |
|---|---|---|---|
| Pd | -2.108039000 | 0.024650000 | -0.006556000 |
| P | -2.082773000 | 2.353055000 | 0.002345000 |
| P | -2.130571000 | -2.303091000 | -0.007194000 |
| C | -3.696451000 | 3.158725000 | -0.414537000 |
| C | -4.871582000 | 2.506256000 | -0.005514000 |
| C | -6.123543000 | 3.062007000 | -0.270768000 |
| C | -6.220207000 | 4.271456000 | -0.963949000 |
| C | -5.059399000 | 4.921681000 | -1.387328000 |
| C | -3.804696000 | 4.370825000 | -1.113644000 |
| C | -1.625604000 | 3.123955000 | 1.621943000 |
| C | -2.156188000 | 4.337898000 | 2.085032000 |
| C | -1.757468000 | 4.861298000 | 3.317775000 |
| C | -0.822618000 | 4.181436000 | 4.100982000 |
| C | -0.291423000 | 2.969879000 | 3.650941000 |
| C | -0.696208000 | 2.441549000 | 2.424723000 |
| C | -0.898098000 | 3.127802000 | -1.190583000 |
| C | -0.685160000 | 2.467916000 | -2.412578000 |
| C | 0.181251000 | 3.000654000 | -3.367601000 |

| | | | |
|---|---|---|---|
| C | 0.861263000 | 4.193579000 | -3.108292000 |
| C | 0.668096000 | 4.850448000 | -1.891472000 |
| C | -0.207145000 | 4.322984000 | -0.938368000 |
| C | -3.580991000 | -3.075885000 | -0.859431000 |
| C | -4.107159000 | -2.407439000 | -1.977599000 |
| C | -5.189768000 | -2.937975000 | -2.679802000 |
| C | -5.773586000 | -4.137733000 | -2.264638000 |
| C | -5.267579000 | -4.803604000 | -1.146562000 |
| C | -4.177192000 | -4.278020000 | -0.448390000 |
| C | -0.676778000 | -3.100233000 | -0.830487000 |
| C | -0.749119000 | -4.306845000 | -1.543580000 |
| C | 0.394474000 | -4.850624000 | -2.134478000 |
| C | 1.623676000 | -4.198689000 | -2.017988000 |
| C | 1.705737000 | -2.994566000 | -1.313701000 |
| C | 0.562616000 | -2.445896000 | -0.731603000 |
| C | -2.153893000 | -3.084989000 | 1.670688000 |
| C | -1.520879000 | -4.300447000 | 1.973517000 |
| C | -1.587732000 | -4.832253000 | 3.263993000 |
| C | -2.289365000 | -4.159352000 | 4.266249000 |
| C | -2.919370000 | -2.946263000 | 3.976281000 |
| C | -2.845102000 | -2.409626000 | 2.690532000 |
| H | 7.677383000 | -2.089519000 | -2.455408000 |
| H | 5.008507000 | -1.085271000 | -1.641583000 |
| H | 7.136039000 | 0.660596000 | -2.217621000 |
| H | 5.405804000 | 1.458508000 | -0.161475000 |
| B | 7.538948000 | -1.571971000 | -1.399849000 |
| B | 5.931081000 | -0.967293000 | -0.908910000 |
| C | 7.208214000 | 0.100832000 | -1.291478000 |

| | | | |
|---|---|---|---|
| B | 6.160174000 | 0.558819000 | -0.018053000 |
| B | 8.466474000 | -1.921320000 | 0.063205000 |
| H | 9.236246000 | -2.808810000 | 0.208088000 |
| B | 6.716178000 | -2.260328000 | 0.004399000 |
| H | 6.330067000 | -3.374078000 | 0.110886000 |
| C | 7.424610000 | -1.454018000 | 1.333666000 |
| H | 7.501772000 | -2.003897000 | 2.264982000 |
| B | 8.766059000 | -0.421166000 | -0.813090000 |
| H | 9.711440000 | -0.159911000 | -1.474640000 |
| B | 8.703150000 | -0.392072000 | 0.953337000 |
| H | 9.625488000 | -0.257820000 | 1.682045000 |
| B | 5.865831000 | -0.943586000 | 0.856813000 |
| H | 4.919705000 | -1.186231000 | 1.525222000 |
| B | 7.090320000 | 0.214255000 | 1.445364000 |
| H | 6.960696000 | 0.743907000 | 2.495246000 |
| B | 7.916701000 | 0.904940000 | 0.040489000 |
| Cl | 8.514648000 | 2.599121000 | -0.091045000 |

## Ts1

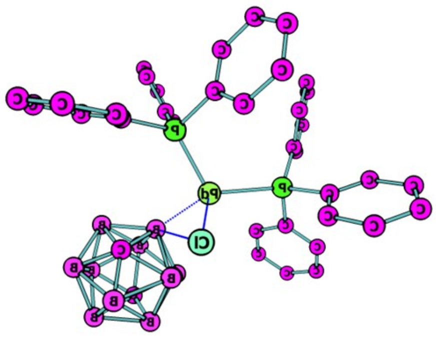

| Atom | x | y | z |
|---|---|---|---|
| Cl | -1.032057000 | -2.749288000 | -0.618569000 |

| | | | |
|---|---|---|---|
| Pd | 0.399542000 | 0.016625000 | -0.479819000 |
| P | 1.683834000 | 0.300821000 | 1.787939000 |
| P | 1.733770000 | -0.105189000 | -2.364943000 |
| C | 3.348520000 | 1.108932000 | 1.837325000 |
| C | 3.561114000 | 2.381167000 | 2.388646000 |
| H | 2.738017000 | 2.922652000 | 2.842900000 |
| C | 4.832989000 | 2.960680000 | 2.365746000 |
| H | 4.980035000 | 3.946012000 | 2.800026000 |
| C | 5.908356000 | 2.277081000 | 1.797552000 |
| H | 6.896892000 | 2.727899000 | 1.783265000 |
| C | 5.706266000 | 1.007964000 | 1.247862000 |
| H | 6.536006000 | 0.465477000 | 0.803103000 |
| C | 4.437413000 | 0.429773000 | 1.262039000 |
| H | 4.299871000 | -0.558268000 | 0.833494000 |
| C | 0.645731000 | 1.385689000 | 2.865820000 |
| C | 0.415063000 | 1.124887000 | 4.225051000 |
| H | 0.854584000 | 0.250740000 | 4.694059000 |
| C | -0.383973000 | 1.982907000 | 4.984303000 |
| H | -0.558589000 | 1.764030000 | 6.034230000 |
| C | -0.954982000 | 3.115467000 | 4.400469000 |
| H | -1.575934000 | 3.780983000 | 4.993735000 |
| C | -0.731333000 | 3.385130000 | 3.048086000 |
| H | -1.178001000 | 4.259777000 | 2.583342000 |
| C | 0.054693000 | 2.521060000 | 2.284580000 |
| H | 0.205912000 | 2.728214000 | 1.228153000 |
| C | 1.931115000 | -1.234341000 | 2.777909000 |

| | | | |
|---|---|---|---|
| C | 1.106237000 | -2.337285000 | 2.504554000 |
| H | 0.371168000 | -2.286797000 | 1.705111000 |
| C | 1.234150000 | -3.514505000 | 3.245791000 |
| H | 0.589264000 | -4.359886000 | 3.022412000 |
| C | 2.191137000 | -3.606489000 | 4.258217000 |
| H | 2.293899000 | -4.524859000 | 4.830020000 |
| C | 3.021280000 | -2.515701000 | 4.531516000 |
| H | 3.769640000 | -2.582638000 | 5.316690000 |
| C | 2.893030000 | -1.335551000 | 3.797444000 |
| H | 3.545744000 | -0.495536000 | 4.015233000 |
| C | 3.153983000 | -1.171610000 | -1.832589000 |
| C | 2.869611000 | -2.313213000 | -1.061334000 |
| H | 1.845935000 | -2.529031000 | -0.767184000 |
| C | 3.896441000 | -3.169991000 | -0.660043000 |
| H | 3.658672000 | -4.047888000 | -0.066044000 |
| C | 5.220052000 | -2.892979000 | -1.010297000 |
| H | 6.019313000 | -3.557092000 | -0.693170000 |
| C | 5.511522000 | -1.754035000 | -1.763981000 |
| H | 6.538877000 | -1.527030000 | -2.035659000 |
| C | 4.487075000 | -0.896732000 | -2.172518000 |
| H | 4.738149000 | -0.011674000 | -2.745605000 |
| C | 1.196110000 | -0.915426000 | -3.937637000 |
| C | 1.039348000 | -2.312365000 | -3.972242000 |
| H | 1.254526000 | -2.910652000 | -3.094098000 |
| C | 0.599182000 | -2.947854000 | -5.133451000 |
| H | 0.485468000 | -4.028128000 | -5.140670000 |

| | | | |
|---|---|---|---|
| C | 0.306907000 | -2.201620000 | -6.277677000 |
| H | -0.033122000 | -2.698431000 | -7.181945000 |
| C | 0.449891000 | -0.814122000 | -6.250306000 |
| H | 0.219623000 | -0.222473000 | -7.131907000 |
| C | 0.884790000 | -0.173058000 | -5.088162000 |
| H | 0.981774000 | 0.906439000 | -5.086853000 |
| C | 2.454463000 | 1.508856000 | -2.872097000 |
| C | 3.296614000 | 1.650473000 | -3.990883000 |
| H | 3.515588000 | 0.795882000 | -4.623344000 |
| C | 3.845499000 | 2.893100000 | -4.308892000 |
| H | 4.493937000 | 2.987580000 | -5.175350000 |
| C | 3.558408000 | 4.011095000 | -3.520905000 |
| H | 3.984780000 | 4.977970000 | -3.773223000 |
| C | 2.716774000 | 3.883675000 | -2.415131000 |
| H | 2.481988000 | 4.750067000 | -1.803427000 |
| C | 2.167322000 | 2.640593000 | -2.094155000 |
| H | 1.506105000 | 2.539846000 | -1.237970000 |
| H | -4.022500000 | -2.314488000 | -2.081210000 |
| H | -3.766113000 | -0.549965000 | -4.440397000 |
| H | -1.601966469 | -1.594596314 | -2.710587653 |
| H | -1.243082000 | 1.120695000 | -4.001034000 |
| B | -3.563839000 | -1.236648000 | -1.880455000 |
| B | -3.432728000 | -0.154188000 | -3.371884000 |
| C | -2.168617469 | -0.822347314 | -2.206269653 |
| B | -1.970672000 | 0.812332000 | -3.119112000 |
| B | -3.966957000 | -0.198296000 | -0.506459000 |

| | | | |
|---|---|---|---|
| H | -4.774994000 | -0.347397000 | 0.350505000 |
| B | -4.580095000 | 0.239786000 | -2.091517000 |
| H | -5.756603000 | 0.322789000 | -2.221375000 |
| C | -3.753924000 | 1.376881000 | -1.130901000 |
| H | -4.322012000 | 2.154557000 | -0.632053000 |
| B | -2.398723531 | -1.013786686 | -0.634518347 |
| H | -2.474124531 | -1.431698686 | 0.478804653 |
| B | -2.441931000 | 0.725807000 | -0.215017000 |
| H | -2.209707000 | 1.261870000 | 0.818432000 |
| B | -3.551414000 | 1.520767000 | -2.790839000 |
| H | -4.030156000 | 2.440716000 | -3.366351000 |
| B | -2.176322000 | 1.757198000 | -1.651152000 |
| H | -1.749181000 | 2.849770000 | -1.468843000 |
| B | -1.353529000 | 0.180278000 | -1.529054000 |

Int2

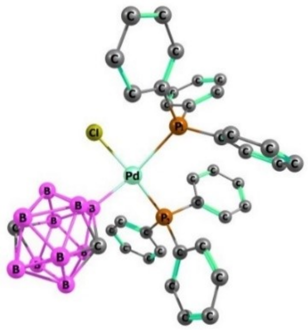

| Atom | x | y | z |
|---|---|---|---|
| Cl | 0.110228000 | -1.470095000 | -2.663172000 |
| Pd | 0.016165000 | -0.034784000 | -0.522909000 |
| P | -2.361296000 | -0.379415000 | -0.055710000 |
| P | 2.282251000 | -0.566740000 | 0.032920000 |

| | | | |
|---|---|---|---|
| C | -2.612465000 | -2.177996000 | 0.351145000 |
| C | -3.386303000 | -2.603515000 | 1.444604000 |
| H | -3.836851000 | -1.880429000 | 2.114550000 |
| C | -3.590301000 | -3.964766000 | 1.683224000 |
| H | -4.191866000 | -4.273556000 | 2.534180000 |
| C | -3.031157000 | -4.920492000 | 0.833910000 |
| H | -3.191741000 | -5.978962000 | 1.021219000 |
| C | -2.267265000 | -4.505948000 | -0.259141000 |
| H | -1.829476000 | -5.239100000 | -0.931294000 |
| C | -2.055432000 | -3.147283000 | -0.501121000 |
| H | -1.452385000 | -2.839786000 | -1.350608000 |
| C | -3.099022000 | 0.449698000 | 1.422674000 |
| C | -4.262831000 | 1.229178000 | 1.368534000 |
| H | -4.778994000 | 1.371556000 | 0.425308000 |
| C | -4.769952000 | 1.829753000 | 2.524467000 |
| H | -5.671684000 | 2.432992000 | 2.462240000 |
| C | -4.128538000 | 1.651966000 | 3.750458000 |
| H | -4.527775000 | 2.114280000 | 4.649043000 |
| C | -2.967284000 | 0.876864000 | 3.815894000 |
| H | -2.458650000 | 0.733957000 | 4.765464000 |
| C | -2.451496000 | 0.291089000 | 2.660429000 |
| H | -1.544413000 | -0.304318000 | 2.723487000 |
| C | -3.565207000 | -0.107475000 | -1.423739000 |
| C | -3.153495000 | 0.593593000 | -2.564994000 |
| H | -2.132427000 | 0.951259000 | -2.636418000 |
| C | -4.041599000 | 0.803438000 | -3.622896000 |

| | | | |
|---|---|---|---|
| H | -3.708125000 | 1.343570000 | -4.504490000 |
| C | -5.344169000 | 0.309114000 | -3.552175000 |
| H | -6.033166000 | 0.468342000 | -4.377322000 |
| C | -5.758791000 | -0.404668000 | -2.423487000 |
| H | -6.768307000 | -0.803255000 | -2.369779000 |
| C | -4.874179000 | -0.617072000 | -1.366937000 |
| H | -5.199905000 | -1.187424000 | -0.501717000 |
| C | 2.582903000 | -2.356618000 | -0.315679000 |
| C | 3.744016000 | -2.824864000 | -0.944281000 |
| H | 4.490631000 | -2.126778000 | -1.307860000 |
| C | 3.947075000 | -4.196999000 | -1.116227000 |
| H | 4.849639000 | -4.546683000 | -1.610276000 |
| C | 2.997850000 | -5.111430000 | -0.659934000 |
| H | 3.157173000 | -6.177557000 | -0.796910000 |
| C | 1.838316000 | -4.649960000 | -0.030773000 |
| H | 1.087351000 | -5.352730000 | 0.319452000 |
| C | 1.630275000 | -3.282684000 | 0.137185000 |
| H | 0.715169000 | -2.937256000 | 0.609355000 |
| C | 3.603540000 | 0.316350000 | -0.892310000 |
| C | 3.535439000 | 0.272276000 | -2.299190000 |
| H | 2.748280000 | -0.301392000 | -2.783363000 |
| C | 4.465713000 | 0.970770000 | -3.068450000 |
| H | 4.406457000 | 0.924782000 | -4.152259000 |
| C | 5.461857000 | 1.732995000 | -2.449896000 |
| H | 6.180814000 | 2.281699000 | -3.051975000 |
| C | 5.524135000 | 1.793451000 | -1.057340000 |

| | | | |
|---|---|---|---|
| H | 6.290071000 | 2.390387000 | -0.569978000 |
| C | 4.600939000 | 1.088865000 | -0.278974000 |
| H | 4.659833000 | 1.148775000 | 0.802431000 |
| C | 2.743355000 | -0.511166000 | 1.822598000 |
| C | 3.992999000 | -0.982979000 | 2.261687000 |
| H | 4.722118000 | -1.345976000 | 1.543236000 |
| C | 4.302652000 | -1.005219000 | 3.621227000 |
| H | 5.273989000 | -1.367988000 | 3.946018000 |
| C | 3.363190000 | -0.574009000 | 4.562238000 |
| H | 3.604132000 | -0.597032000 | 5.621513000 |
| C | 2.113461000 | -0.122350000 | 4.137311000 |
| H | 1.376364000 | 0.209093000 | 4.863547000 |
| C | 1.805748000 | -0.090678000 | 2.774904000 |
| H | 0.835561000 | 0.264551000 | 2.442315000 |
| H | 1.373199000 | 4.798849000 | -2.462986000 |
| H | 2.964166000 | 4.734615000 | 0.050686000 |
| H | 2.151874000 | 2.405024000 | -1.263200000 |
| H | 2.216479000 | 2.347555000 | 1.547136000 |
| B | 0.880066000 | 4.422480000 | -1.453824000 |
| B | 1.832416000 | 4.384457000 | 0.048982000 |
| C | 1.368841000 | 2.946783000 | -0.747136000 |
| B | 1.369704000 | 2.909852000 | 0.945971000 |
| B | -0.823377000 | 4.550833000 | -1.021548000 |
| H | -1.655827000 | 5.152112000 | -1.612586000 |
| B | 0.411526000 | 5.421339000 | -0.074308000 |
| H | 0.404139000 | 6.605734000 | -0.037474000 |

| | | | |
|---|---|---|---|
| C | -0.820720000 | 4.520464000 | 0.682915000 |
| H | -1.602239000 | 5.071984000 | 1.193605000 |
| B | -0.161777000 | 2.969328000 | -1.468955000 |
| H | -0.352141000 | 2.429821000 | -2.505549000 |
| B | -1.281248000 | 3.086780000 | -0.114849000 |
| H | -2.417837000 | 2.768962000 | -0.120277000 |
| B | 0.716764000 | 4.484488000 | 1.412195000 |
| H | 0.913765000 | 5.046620000 | 2.436698000 |
| B | -0.330184000 | 3.035869000 | 1.389668000 |
| H | -0.823585000 | 2.682146000 | 2.404604000 |
| B | 0.120470000 | 1.985518000 | 0.027765000 |

TS2

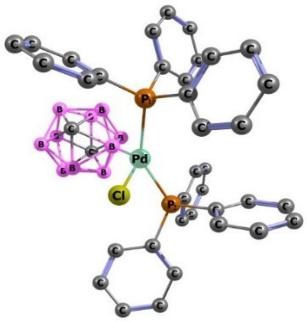

| Atom | x | y | z |
|---|---|---|---|
| Cl | 3.238865000 | 0.821063000 | -0.074578000 |
| Pd | 0.770934000 | 0.116369000 | -0.240444000 |
| P | 0.552994000 | -0.401312000 | 2.092274000 |
| P | 1.658799000 | 0.600899000 | -2.580778000 |
| C | 0.023853000 | -2.143047000 | 2.384604000 |
| C | 0.810637000 | -3.162939000 | 1.817695000 |

| | | | |
|---|---|---|---|
| H | 1.704193000 | -2.909429000 | 1.253371000 |
| C | 0.455804000 | -4.502205000 | 1.969706000 |
| H | 1.080049000 | -5.276603000 | 1.532854000 |
| C | -0.705947000 | -4.845426000 | 2.667771000 |
| H | -0.988145000 | -5.888685000 | 2.777798000 |
| C | -1.503959000 | -3.841397000 | 3.215198000 |
| H | -2.412244000 | -4.097637000 | 3.753431000 |
| C | -1.140450000 | -2.497877000 | 3.080122000 |
| H | -1.771605000 | -1.733766000 | 3.520975000 |
| C | 2.102157000 | -0.228442000 | 3.090857000 |
| C | 2.670361000 | -1.285410000 | 3.814683000 |
| H | 2.233376000 | -2.276762000 | 3.777573000 |
| C | 3.804996000 | -1.071642000 | 4.603481000 |
| H | 4.234006000 | -1.901777000 | 5.158184000 |
| C | 4.376683000 | 0.197563000 | 4.683725000 |
| H | 5.257187000 | 0.362420000 | 5.298744000 |
| C | 3.811914000 | 1.257138000 | 3.968102000 |
| H | 4.253585000 | 2.248334000 | 4.019266000 |
| C | 2.688252000 | 1.046107000 | 3.172170000 |
| H | 2.273319000 | 1.872839000 | 2.605992000 |
| C | -0.583113000 | 0.672208000 | 3.073207000 |
| C | -1.200831000 | 1.764507000 | 2.449994000 |
| H | -1.036473000 | 1.936058000 | 1.391466000 |
| C | -2.011850000 | 2.636067000 | 3.182924000 |
| H | -2.484329000 | 3.477989000 | 2.684893000 |
| C | -2.208364000 | 2.424865000 | 4.547345000 |

| | | | |
|---|---|---|---|
| H | -2.838826000 | 3.100509000 | 5.118708000 |
| C | -1.581132000 | 1.348024000 | 5.183173000 |
| H | -1.719342000 | 1.188548000 | 6.248953000 |
| C | -0.766522000 | 0.482351000 | 4.455450000 |
| H | -0.263990000 | -0.334264000 | 4.965577000 |
| C | 2.176945000 | 2.374318000 | -2.535844000 |
| C | 3.404094000 | 2.851678000 | -3.012751000 |
| H | 4.151872000 | 2.162836000 | -3.388293000 |
| C | 3.691564000 | 4.217562000 | -2.975704000 |
| H | 4.652620000 | 4.571194000 | -3.339385000 |
| C | 2.757795000 | 5.123666000 | -2.468896000 |
| H | 2.985467000 | 6.185743000 | -2.442069000 |
| C | 1.533298000 | 4.655309000 | -1.987994000 |
| H | 0.802177000 | 5.349708000 | -1.582754000 |
| C | 1.247352000 | 3.289071000 | -2.015641000 |
| H | 0.299859000 | 2.931326000 | -1.619781000 |
| C | 3.083423000 | -0.498762000 | -3.010908000 |
| C | 3.825863000 | -0.296436000 | -4.186824000 |
| H | 3.592293000 | 0.532588000 | -4.846924000 |
| C | 4.855898000 | -1.171338000 | -4.536418000 |
| H | 5.429271000 | -0.990669000 | -5.441560000 |
| C | 5.135467000 | -2.280648000 | -3.735993000 |
| H | 5.930998000 | -2.967010000 | -4.013159000 |
| C | 4.380152000 | -2.509497000 | -2.584155000 |
| H | 4.582961000 | -3.376174000 | -1.960993000 |
| C | 3.366301000 | -1.621629000 | -2.220249000 |

| | | | |
|---|---|---|---|
| H | 2.801163000 | -1.790846000 | -1.310525000 |
| C | 0.805231000 | 0.552077000 | -4.247784000 |
| C | 0.670614000 | -0.673909000 | -4.922728000 |
| H | 1.063550000 | -1.583225000 | -4.480437000 |
| C | 0.055309000 | -0.742823000 | -6.172981000 |
| H | -0.033461000 | -1.702974000 | -6.674248000 |
| C | -0.433709000 | 0.415745000 | -6.780925000 |
| H | -0.908009000 | 0.363747000 | -7.757220000 |
| C | -0.296721000 | 1.641508000 | -6.128482000 |
| H | -0.662217000 | 2.552789000 | -6.594323000 |
| C | 0.315233000 | 1.709387000 | -4.874609000 |
| H | 0.411420000 | 2.674668000 | -4.391095000 |
| H | -4.824707000 | 0.191221000 | 0.368829000 |
| H | -4.045032000 | -2.678418000 | 0.455389000 |
| H | -2.289070000 | -0.633721000 | 1.156326000 |
| H | -1.186162000 | -2.871469000 | -0.155365000 |
| B | -4.043239000 | -0.241476000 | -0.410329000 |
| B | -3.576667000 | -1.955589000 | -0.358692000 |
| C | -2.489354000 | -0.723673000 | 0.096908000 |
| B | -1.823013000 | -2.048804000 | -0.715798000 |
| B | -3.778459000 | 0.350852000 | -2.048223000 |
| H | -4.441874000 | 1.147448000 | -2.622788000 |
| B | -4.394347000 | -1.300683000 | -1.778226000 |
| H | -5.469765000 | -1.604695000 | -2.173182000 |
| C | -3.124240000 | -0.988648000 | -2.871469000 |
| H | -3.340061000 | -1.078217000 | -3.930226000 |

| | | | |
|---|---|---|---|
| B | -2.570609000 | 0.704861000 | -0.801844000 |
| H | -2.450536000 | 1.772600000 | -0.303546000 |
| B | -2.037288000 | 0.245226000 | -2.407156000 |
| H | -1.618225000 | 0.976458000 | -3.229437000 |
| B | -3.021295000 | -2.424201000 | -1.963410000 |
| H | -3.183571000 | -3.478057000 | -2.481115000 |
| B | -1.565032000 | -1.472143000 | -2.353408000 |
| H | -0.809638000 | -1.930096000 | -3.135621000 |
| B | -1.136247000 | -0.392109000 | -1.002420000 |

## Int3

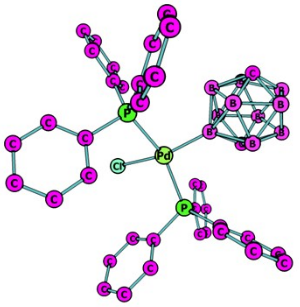

| Atom | x | y | z |
|---|---|---|---|
| Pd | 0.020141000 | 0.113623000 | -0.385427000 |
| P | -2.359573000 | 0.404388000 | -0.000348000 |
| P | 2.374679000 | 0.576490000 | -0.026683000 |
| C | -2.802637000 | 2.176345000 | 0.349604000 |
| C | -2.893639000 | 3.069999000 | -0.732079000 |
| C | -3.222731000 | 4.407908000 | -0.519536000 |
| C | -3.458338000 | 4.881589000 | 0.772942000 |
| C | -3.370096000 | 4.003301000 | 1.852683000 |
| C | -3.047415000 | 2.659776000 | 1.643898000 |

| | | | |
|---|---|---|---|
| C | -3.475304000 | 0.012187000 | -1.413507000 |
| C | -4.840381000 | 0.352903000 | -1.391406000 |
| C | -5.659316000 | 0.054703000 | -2.479382000 |
| C | -5.125816000 | -0.575297000 | -3.608343000 |
| C | -3.769603000 | -0.897677000 | -3.648107000 |
| C | -2.947258000 | -0.602052000 | -2.557308000 |
| C | -3.065330000 | -0.482780000 | 1.460245000 |
| C | -2.400487000 | -0.348859000 | 2.692811000 |
| C | -2.896265000 | -0.960545000 | 3.843164000 |
| C | -4.044545000 | -1.753785000 | 3.774709000 |
| C | -4.690554000 | -1.925949000 | 2.550781000 |
| C | -4.210791000 | -1.289275000 | 1.402114000 |
| C | 2.419813000 | 2.333258000 | 0.562874000 |
| C | 1.378200000 | 2.791415000 | 1.385300000 |
| C | 1.394661000 | 4.086913000 | 1.901844000 |
| C | 2.446239000 | 4.950925000 | 1.590609000 |
| C | 3.477209000 | 4.510707000 | 0.759551000 |
| C | 3.466602000 | 3.210781000 | 0.249383000 |
| C | 3.343387000 | -0.278110000 | 1.306541000 |
| C | 3.086215000 | 0.044484000 | 2.649755000 |
| C | 3.748065000 | -0.616986000 | 3.683873000 |
| C | 4.682888000 | -1.614436000 | 3.396967000 |
| C | 4.946808000 | -1.944693000 | 2.067507000 |
| C | 4.280306000 | -1.286841000 | 1.031027000 |
| C | 3.466984000 | 0.491422000 | -1.507776000 |
| C | 4.863684000 | 0.643370000 | -1.429937000 |

| | | | |
|---|---|---|---|
| C | 5.648302000 | 0.585891000 | -2.582428000 |
| C | 5.051017000 | 0.372050000 | -3.827023000 |
| C | 3.666848000 | 0.217724000 | -3.913957000 |
| C | 2.878302000 | 0.277013000 | -2.763174000 |
| Cl | 0.021557000 | 2.127817000 | -1.934339000 |
| B | -0.830337000 | -4.417696000 | -1.311051000 |
| B | -1.238639000 | -4.535362000 | 0.417388000 |
| C | -1.199927000 | -3.029813000 | -0.383849000 |
| B | -0.646802000 | -3.046909000 | 1.218832000 |
| B | 0.925569000 | -4.369652000 | -1.457140000 |
| B | 0.153290000 | -5.405220000 | -0.227841000 |
| C | 1.475581000 | -4.399358000 | 0.153311000 |
| B | 0.009932000 | -2.858032000 | -1.559810000 |
| B | 1.509675000 | -2.893652000 | -0.645718000 |
| B | 0.265599000 | -4.557481000 | 1.336869000 |
| B | 1.101578000 | -3.002050000 | 1.080271000 |
| B | 0.129276000 | -1.953025000 | 0.008963000 |

PPh$_3$

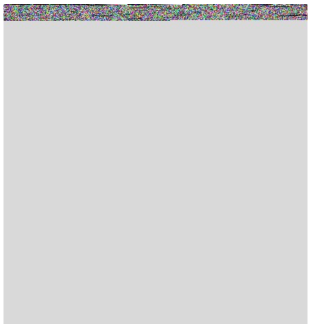

| Atom | x | y | z |
|---|---|---|---|
| P | 0.000060000 | -0.000150000 | -1.203446000 |

| | | | |
|---|---|---|---|
| C | -1.153156000 | -1.209717000 | -0.402384000 |
| C | -1.307393000 | -2.455727000 | -1.034848000 |
| H | -0.755867000 | -2.665937000 | -1.948480000 |
| C | -2.162742000 | -3.424699000 | -0.509061000 |
| H | -2.265915000 | -4.383656000 | -1.010117000 |
| C | -2.894238000 | -3.156446000 | 0.650696000 |
| H | -3.568522000 | -3.905942000 | 1.056488000 |
| C | -2.760916000 | -1.917621000 | 1.281208000 |
| H | -3.330380000 | -1.700612000 | 2.181398000 |
| C | -1.895774000 | -0.951836000 | 0.760626000 |
| H | -1.801317000 | 0.007397000 | 1.260554000 |
| C | 1.624221000 | -0.393808000 | -0.402347000 |
| C | 1.772018000 | -1.163570000 | 0.762231000 |
| H | 0.893979000 | -1.559883000 | 1.263220000 |
| C | 3.040965000 | -1.429654000 | 1.282966000 |
| H | 3.137631000 | -2.029512000 | 2.184386000 |
| C | 4.180630000 | -0.927796000 | 0.651041000 |
| H | 5.166827000 | -1.136823000 | 1.056997000 |
| C | 4.047376000 | -0.162491000 | -0.510250000 |
| H | 4.929574000 | 0.225980000 | -1.012372000 |
| C | 2.780535000 | 0.093577000 | -1.036130000 |
| H | 2.686924000 | 0.674465000 | -1.950940000 |
| C | -0.470908000 | 1.603410000 | -0.402642000 |
| C | 0.123169000 | 2.117273000 | 0.760795000 |
| H | 0.906223000 | 1.555517000 | 1.261015000 |
| C | -0.280746000 | 3.349373000 | 1.281344000 |

| | | | |
|---|---|---|---|
| H | 0.191505000 | 3.733790000 | 2.181857000 |
| C | -1.286555000 | 4.084484000 | 0.650478000 |
| H | -1.598553000 | 5.043121000 | 1.056362000 |
| C | -1.884094000 | 3.585405000 | -0.509670000 |
| H | -2.662708000 | 4.154419000 | -1.010952000 |
| C | -1.472466000 | 2.360193000 | -1.035449000 |
| H | -1.929851000 | 1.987828000 | -1.949352000 |

## pyrrolidine

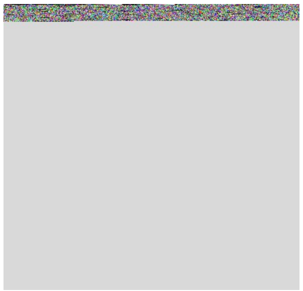

| Atom | x | y | z |
|---|---|---|---|
| C | 1.161477000 | -0.448459000 | 0.171310000 |
| C | -1.161682000 | -0.448020000 | 0.171191000 |
| C | -0.778616000 | 1.030194000 | -0.056469000 |
| C | 0.779043000 | 1.029854000 | -0.056627000 |
| H | 1.322947000 | -0.629572000 | 1.251001000 |
| H | 2.076060000 | -0.742366000 | -0.355656000 |
| H | -2.076293000 | -0.741480000 | -0.355977000 |
| H | -1.323425000 | -0.629213000 | 1.250824000 |
| H | -1.161338000 | 1.380747000 | -1.019838000 |
| H | -1.199172000 | 1.677022000 | 0.719576000 |

| | | | |
|---|---|---|---|
| H | 1.161702000 | 1.379937000 | -1.020194000 |
| H | 1.200064000 | 1.676722000 | 0.719132000 |
| N | -0.000209000 | -1.172246000 | -0.357243000 |
| H | -0.000413000 | -2.147495000 | -0.064592000 |

## 3-ethynylquinoline

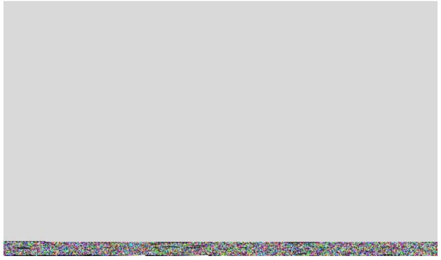

| **Atom** | **x** | **y** | **z** |
|---|---|---|---|
| C | 0.841609000 | 0.780100000 | -0.000202000 |
| C | 0.530707000 | -0.617853000 | 0.000019000 |
| C | 1.590824000 | -1.564221000 | 0.000020000 |
| C | 2.901418000 | -1.141091000 | -0.000190000 |
| C | 3.208115000 | 0.243252000 | -0.000408000 |
| C | 2.201233000 | 1.184501000 | -0.000415000 |
| N | -0.130040000 | 1.743767000 | -0.000216000 |
| C | -1.384936000 | 1.359019000 | -0.000018000 |
| C | -1.812906000 | -0.008423000 | 0.000213000 |
| C | -0.834226000 | -0.989375000 | 0.000228000 |
| C | -3.205001000 | -0.319972000 | 0.000418000 |
| C | -4.390433000 | -0.565479000 | 0.000591000 |
| H | -5.434286000 | -0.786049000 | 0.000752000 |
| H | 1.349870000 | -2.624406000 | 0.000188000 |
| H | 3.707933000 | -1.868819000 | -0.000188000 |

| | | | |
|---|---|---|---|
| H | 4.247342000 | 0.559959000 | -0.000572000 |
| H | 2.413703000 | 2.249061000 | -0.000580000 |
| H | -2.142411000 | 2.141396000 | -0.000033000 |
| H | -1.110298000 | -2.040261000 | 0.000399000 |

## H-Base$^+$Cl$^-$

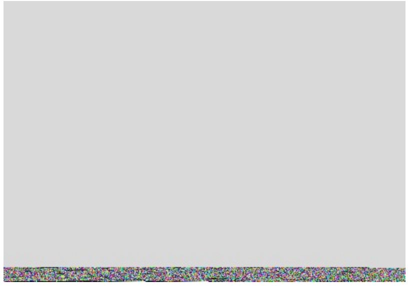

| **Atom** | **x** | **y** | **z** |
|---|---|---|---|
| Cl | 2.438361000 | 0.468614000 | 0.928839000 |
| H | -0.791955000 | 0.561920000 | 2.195012000 |
| C | -1.038465000 | 1.304646000 | 0.214975000 |
| C | -0.852199000 | -1.065020000 | 0.843848000 |
| C | -1.746969000 | 0.415736000 | -0.820070000 |
| H | -1.693837000 | 2.025886000 | 0.704559000 |
| H | -0.176113000 | 1.827524000 | -0.204550000 |
| C | -1.056206000 | -0.950579000 | -0.666790000 |
| H | -0.040160000 | -1.729099000 | 1.144752000 |
| H | -1.773337000 | -1.346159000 | 1.360612000 |

| | | | |
|---|---|---|---|
| H | -1.656675000 | 0.827406000 | -1.828400000 |
| H | -2.813747000 | 0.331349000 | -0.584022000 |
| H | -0.083733000 | -0.950409000 | -1.170577000 |
| H | -1.650052000 | -1.779376000 | -1.060574000 |
| N | -0.483465000 | 0.340637000 | 1.247357000 |
| H | 0.609861000 | 0.416972000 | 1.226481000 |

Product

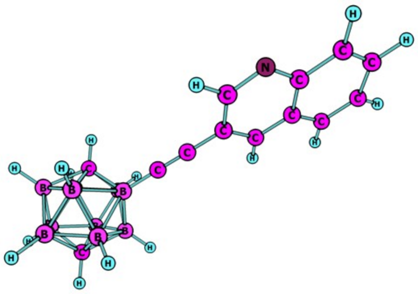

| Atom | x | y | z |
|---|---|---|---|
| C | 4.600910000 | -0.735623000 | 0.008530000 |
| C | 4.101833000 | 0.606616000 | -0.007177000 |
| C | 5.022572000 | 1.689314000 | -0.005358000 |
| C | 6.378577000 | 1.449524000 | 0.011208000 |
| C | 6.871573000 | 0.120085000 | 0.026620000 |
| C | 6.002854000 | -0.950101000 | 0.025328000 |
| N | 3.770142000 | -1.823263000 | 0.007447000 |
| C | 2.474628000 | -1.613871000 | -0.009396000 |
| C | 1.862499000 | -0.317480000 | -0.025239000 |
| C | 2.699485000 | 0.788216000 | -0.023200000 |
| C | 0.443471000 | -0.197836000 | -0.043475000 |
| C | -0.771514000 | -0.111058000 | -0.061317000 |

| | | | |
|---|---|---|---|
| H | 4.638184000 | 2.706248000 | -0.017293000 |
| H | 7.078112000 | 2.280611000 | 0.012416000 |
| H | 7.944308000 | -0.051296000 | 0.039448000 |
| H | 6.358501000 | -1.975656000 | 0.036728000 |
| H | 1.831479000 | -2.492679000 | -0.011951000 |
| H | 2.282778000 | 1.791585000 | -0.034625000 |
| H | -5.050753000 | 0.484073000 | 2.650558000 |
| H | -5.237666000 | -2.157825000 | 1.312585000 |
| H | -2.766816000 | -1.029320000 | 2.028448000 |
| H | -2.813992000 | -2.509690000 | -0.354231000 |
| B | -4.672389000 | 0.357712000 | 1.535712000 |
| B | -4.784502000 | -1.231364000 | 0.730962000 |
| C | -3.280872000 | -0.569246000 | 1.191918000 |
| B | -3.326999000 | -1.446270000 | -0.273701000 |
| B | -4.671300000 | 1.608916000 | 0.288248000 |
| H | -5.178299000 | 2.675995000 | 0.367842000 |
| B | -5.686956000 | 0.152650000 | 0.103507000 |
| H | -6.865389000 | 0.258017000 | 0.060527000 |
| C | -4.714253000 | 0.731365000 | -1.175776000 |
| H | -5.222047000 | 1.192795000 | -2.015343000 |
| B | -3.144843000 | 1.125506000 | 1.029161000 |
| H | -2.511659000 | 1.755360000 | 1.805714000 |
| B | -3.212619000 | 1.396620000 | -0.716958000 |
| H | -2.754736000 | 2.319502000 | -1.299772000 |
| B | -4.854038000 | -0.962388000 | -1.013929000 |
| H | -5.481907000 | -1.593874000 | -1.794282000 |

| | | | |
|---|---|---|---|
| B | -3.326046000 | -0.195469000 | -1.523829000 |
| H | -2.942387000 | -0.319030000 | -2.636604000 |
| B | -2.290166000 | 0.011806000 | -0.094831000 |

## Ts3

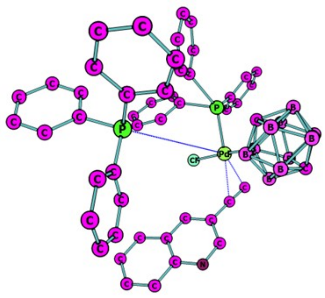

| Atom | x | y | z |
|---|---|---|---|
| Pd | 3.422927000 | -2.968934000 | 0.715982000 |
| Cl | 4.963901000 | -4.474805000 | -0.660310000 |
| P | -1.011614000 | -2.369948000 | -3.592928000 |
| P | 3.949872000 | -0.995619000 | -0.560224000 |
| C | 0.159314000 | -7.959312000 | 0.421525000 |
| C | 1.288672000 | -7.612447000 | -0.389275000 |
| C | 1.454542000 | -8.237095000 | -1.656108000 |
| C | 0.538446000 | -9.165049000 | -2.098533000 |
| C | -0.579148000 | -9.507650000 | -1.294932000 |
| C | -0.766696000 | -8.918800000 | -0.061483000 |
| N | -0.058598000 | -7.397621000 | 1.650830000 |
| C | 0.801507000 | -6.507878000 | 2.087345000 |
| C | 1.963076000 | -6.087858000 | 1.359616000 |
| C | 2.198589000 | -6.656718000 | 0.114596000 |

| | | | |
|---|---|---|---|
| C | 2.859695000 | -5.143023000 | 1.935175000 |
| C | 3.639959000 | -4.355856000 | 2.470530000 |
| B | -0.320726000 | -0.124515000 | 1.791017000 |
| B | 0.998137000 | 0.834588000 | 2.507554000 |
| C | 1.298916000 | -0.346161000 | 1.314725000 |
| B | 2.460125000 | -0.198729000 | 2.544982000 |
| B | -0.644385000 | -1.495143000 | 2.849090000 |
| B | -0.227931000 | 0.098142000 | 3.539957000 |
| C | 0.509569000 | -1.333208000 | 4.093694000 |
| B | 0.338157000 | -1.742803000 | 1.390389000 |
| B | 0.816608000 | -2.513802000 | 2.899492000 |
| B | 1.495076000 | 0.054011000 | 4.008590000 |
| B | 2.136614000 | -1.563007000 | 3.611101000 |
| B | 2.080960000 | -1.813884000 | 1.844308000 |
| C | 3.498549000 | 0.065310000 | -2.423831000 |
| C | 2.117753000 | 0.235860000 | -2.681058000 |
| C | 4.377476000 | 1.120785000 | -2.738928000 |
| C | 1.643829000 | 1.437012000 | -3.195412000 |
| C | 3.879523000 | 2.319532000 | -3.250009000 |
| C | 2.515154000 | 2.495094000 | -3.481945000 |
| C | 4.168711000 | -1.582447000 | -2.445814000 |
| C | 5.466204000 | -1.657105000 | -3.050358000 |
| C | 3.193968000 | -2.521586000 | -2.925899000 |
| C | 5.728412000 | -2.516613000 | -4.101979000 |
| C | 3.463401000 | -3.371383000 | -3.980780000 |
| C | 4.727164000 | -3.367245000 | -4.587964000 |

| | | | |
|---|---|---|---|
| C | 5.655990000 | -0.467771000 | -0.112706000 |
| C | 5.851002000 | 0.868253000 | 0.281390000 |
| C | 6.728095000 | -1.373729000 | -0.014773000 |
| C | 7.093293000 | 1.293147000 | 0.757833000 |
| C | 7.966736000 | -0.944528000 | 0.464152000 |
| C | 8.152012000 | 0.387111000 | 0.848927000 |
| C | -1.989628000 | -0.795742000 | -3.595590000 |
| C | -2.581107000 | -0.231663000 | -4.737014000 |
| C | -2.109043000 | -0.114899000 | -2.370565000 |
| C | -3.278599000 | 0.976424000 | -4.653614000 |
| C | -2.814476000 | 1.086338000 | -2.285490000 |
| C | -3.399246000 | 1.636981000 | -3.429196000 |
| C | -2.276231000 | -3.639142000 | -3.129463000 |
| C | -3.663074000 | -3.451595000 | -3.245884000 |
| C | -1.802491000 | -4.853452000 | -2.603807000 |
| C | -4.550706000 | -4.456462000 | -2.854640000 |
| C | -2.688945000 | -5.861833000 | -2.221333000 |
| C | -4.066248000 | -5.663390000 | -2.344636000 |
| C | -0.737209000 | -2.680911000 | -5.398711000 |
| C | 0.374861000 | -2.056653000 | -5.992306000 |
| C | -1.538757000 | -3.517941000 | -6.190284000 |
| C | 0.665273000 | -2.248121000 | -7.343663000 |
| C | -1.240412000 | -3.719103000 | -7.540697000 |
| C | -0.141098000 | -3.083438000 | -8.121455000 |

Int4

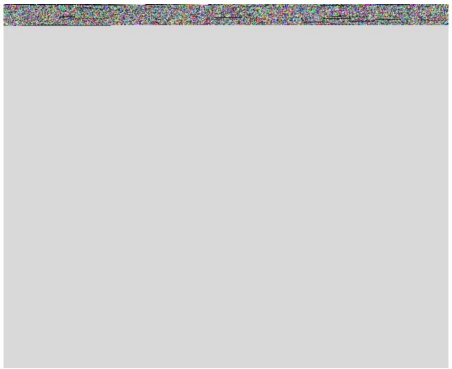

| Atom | x | y | z |
|---|---|---|---|
| Pd | 0.029200000 | 0.172957000 | 1.167504000 |
| Cl | -0.669571000 | -1.937437000 | 2.373801000 |
| P | 1.583152000 | -1.043113000 | -0.155170000 |
| C | -5.710459000 | 0.247637000 | -0.232711000 |
| C | -4.956669000 | -0.884543000 | 0.220004000 |
| C | -5.412563000 | -2.195354000 | -0.089467000 |
| C | -6.567124000 | -2.373696000 | -0.817991000 |
| C | -7.311306000 | -1.252720000 | -1.266330000 |
| C | -6.894438000 | 0.030361000 | -0.981643000 |
| N | -5.328975000 | 1.535742000 | 0.032066000 |
| C | -4.229291000 | 1.727117000 | 0.721851000 |
| C | -3.401954000 | 0.669255000 | 1.221324000 |
| C | -3.782097000 | -0.642378000 | 0.965546000 |
| C | -2.246543000 | 0.965606000 | 1.998480000 |
| C | -1.273417000 | 1.202033000 | 2.710962000 |

| | | | |
|---|---|---|---|
| H | -0.707126000 | 1.485079000 | 3.573772000 |
| H | -4.835460000 | -3.047203000 | 0.260468000 |
| H | -6.914245000 | -3.375892000 | -1.052108000 |
| H | -8.220484000 | -1.411584000 | -1.839573000 |
| H | -7.451862000 | 0.899864000 | -1.315375000 |
| H | -3.944281000 | 2.757688000 | 0.925072000 |
| H | -3.166531000 | -1.460705000 | 1.331165000 |
| H | -0.466805000 | 4.406824000 | -2.593165000 |
| H | 2.438062000 | 3.780356000 | -2.621827000 |
| H | 0.508548000 | 1.867865000 | -1.987346000 |
| H | 2.958822000 | 1.739322000 | -0.606724000 |
| B | 0.104446000 | 4.188758000 | -1.578209000 |
| B | 1.846009000 | 3.814923000 | -1.596209000 |
| C | 0.679764000 | 2.619552000 | -1.227737000 |
| B | 2.137911000 | 2.552569000 | -0.368104000 |
| B | -0.296728000 | 4.854617000 | 0.001631000 |
| H | -1.089791000 | 5.706223000 | 0.225020000 |
| B | 1.257385000 | 5.258423000 | -0.774817000 |
| H | 1.499419000 | 6.380167000 | -1.070310000 |
| C | 1.173421000 | 4.789377000 | 0.861277000 |
| H | 1.345052000 | 5.552766000 | 1.612052000 |
| B | -0.659948000 | 3.151040000 | -0.337232000 |
| H | -1.745081000 | 2.730057000 | -0.556113000 |
| B | 0.011740000 | 3.599645000 | 1.227729000 |
| H | -0.568008000 | 3.684618000 | 2.254128000 |
| B | 2.517923000 | 4.242560000 | -0.022461000 |

| | | | |
|---|---|---|---|
| H | 3.597892000 | 4.687301000 | 0.177965000 |
| B | 1.740997000 | 3.208651000 | 1.213607000 |
| H | 2.316410000 | 3.009933000 | 2.231258000 |
| B | 0.583190000 | 2.088431000 | 0.427288000 |
| C | 1.368061000 | -2.884093000 | -0.187852000 |
| C | 0.869038000 | -3.570698000 | -1.303033000 |
| C | 1.747367000 | -3.619306000 | 0.948441000 |
| C | 0.755159000 | -4.963817000 | -1.284216000 |
| C | 1.640891000 | -5.007999000 | 0.959597000 |
| C | 1.142010000 | -5.685589000 | -0.156024000 |
| H | 0.577856000 | -3.033697000 | -2.198148000 |
| H | 2.122934000 | -3.108153000 | 1.827348000 |
| H | 0.369980000 | -5.479685000 | -2.159667000 |
| H | 1.939670000 | -5.559947000 | 1.846289000 |
| H | 1.056386000 | -6.768808000 | -0.143901000 |
| C | 3.323878000 | -0.910591000 | 0.428831000 |
| C | 3.631458000 | -0.090225000 | 1.522737000 |
| C | 4.345100000 | -1.682709000 | -0.153670000 |
| C | 4.937991000 | -0.021379000 | 2.011587000 |
| C | 5.650987000 | -1.603642000 | 0.328907000 |
| C | 5.949921000 | -0.771282000 | 1.411402000 |
| H | 2.845385000 | 0.491120000 | 1.994104000 |
| H | 4.118918000 | -2.357964000 | -0.973448000 |
| H | 5.160584000 | 0.617044000 | 2.861760000 |
| H | 6.432208000 | -2.200817000 | -0.133035000 |
| H | 6.966646000 | -0.716985000 | 1.790597000 |

| | | | |
|---|---|---|---|
| C | 1.536671000 | -0.600427000 | -1.945488000 |
| C | 2.672273000 | -0.303745000 | -2.711086000 |
| C | 0.272067000 | -0.559163000 | -2.562170000 |
| C | 2.546967000 | 0.016959000 | -4.065903000 |
| C | 0.151469000 | -0.254254000 | -3.918633000 |
| C | 1.291193000 | 0.036901000 | -4.673821000 |
| H | 3.656152000 | -0.308840000 | -2.255535000 |
| H | -0.622366000 | -0.757243000 | -1.976305000 |
| H | 3.436153000 | 0.253677000 | -4.643390000 |
| H | -0.831878000 | -0.232088000 | -4.379697000 |
| H | 1.198308000 | 0.285386000 | -5.727186000 |

TS1-C

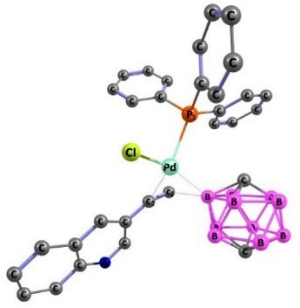

| **Atom** | **x** | **y** | **z** |
|---|---|---|---|
| Pd | -0.060709000 | 0.163849000 | -1.049308000 |
| P | 2.190420000 | 0.160017000 | 0.057763000 |
| C | -5.190520000 | 2.402197000 | -3.209902000 |
| C | -4.640020000 | 2.495886000 | -1.890579000 |
| C | -5.395571000 | 3.126256000 | -0.864617000 |
| C | -6.642675000 | 3.643124000 | -1.138112000 |

| | | | |
|---|---|---|---|
| C | -7.184781000 | 3.550563000 | -2.444712000 |
| C | -6.475787000 | 2.943066000 | -3.460398000 |
| N | -4.517562000 | 1.800232000 | -4.241862000 |
| C | -3.330839000 | 1.300592000 | -3.997109000 |
| C | -2.679870000 | 1.345240000 | -2.717204000 |
| C | -3.357721000 | 1.946139000 | -1.665194000 |
| C | -1.376276000 | 0.771581000 | -2.575899000 |
| C | -0.322871000 | 0.245701000 | -3.103286000 |
| H | 0.306129000 | 0.229254000 | -3.979160000 |
| H | -4.971634000 | 3.190626000 | 0.133976000 |
| H | -7.217528000 | 4.123909000 | -0.351929000 |
| H | -8.170116000 | 3.962747000 | -2.643773000 |
| H | -6.875849000 | 2.861773000 | -4.466256000 |
| H | -2.814404000 | 0.822017000 | -4.828519000 |
| H | -2.901860000 | 1.977490000 | -0.678828000 |
| H | 0.706021000 | -4.988720000 | -0.308528000 |
| H | 2.238338000 | -4.725134000 | -2.843112000 |
| H | 1.797251000 | -2.561446000 | -1.154118000 |
| H | 1.778297000 | -2.005197000 | -3.903861000 |
| B | 0.246767000 | -4.391634000 | -1.222022000 |
| B | 1.167609000 | -4.230693000 | -2.740086000 |
| C | 0.927990000 | -2.900086000 | -1.702231000 |
| B | 0.892929000 | -2.582831000 | -3.372083000 |
| B | -1.471913000 | -4.216166000 | -1.596231000 |
| H | -2.360869000 | -4.795363000 | -1.071168000 |
| B | -0.381670000 | -5.079434000 | -2.720414000 |

| | | | |
|---|---|---|---|
| H | -0.551636000 | -6.230716000 | -2.938991000 |
| C | -1.495994000 | -3.914998000 | -3.269255000 |
| H | -2.357167000 | -4.265035000 | -3.827367000 |
| B | -0.589678000 | -2.840296000 | -0.919574000 |
| H | -0.703761000 | -2.472914000 | 0.197082000 |
| B | -1.739722000 | -2.573081000 | -2.232706000 |
| H | -2.808183000 | -2.073523000 | -2.140135000 |
| B | 0.017423000 | -3.964457000 | -4.051682000 |
| H | 0.113882000 | -4.367187000 | -5.161022000 |
| B | -0.825745000 | -2.425108000 | -3.750478000 |
| H | -1.289695000 | -1.845276000 | -4.671260000 |
| B | -0.186356000 | -1.686176000 | -2.249208000 |
| C | 2.805155000 | 1.906111000 | -0.013500000 |
| C | 4.155297000 | 2.215764000 | -0.253645000 |
| C | 1.882052000 | 2.946074000 | 0.201362000 |
| C | 4.588544000 | 3.551193000 | -0.286496000 |
| C | 2.298594000 | 4.286207000 | 0.175544000 |
| C | 3.633777000 | 4.520493000 | -0.071376000 |
| H | 4.879929000 | 1.425321000 | -0.422157000 |
| H | 0.839439000 | 2.713634000 | 0.402391000 |
| H | 5.632143000 | 3.788356000 | -0.474739000 |
| H | 1.583981000 | 5.087325000 | 0.343119000 |
| C | 3.557048000 | -0.793049000 | -0.745251000 |
| C | 4.276026000 | -1.796556000 | -0.077411000 |
| C | 3.844448000 | -0.542885000 | -2.100565000 |
| C | 5.261638000 | -2.528034000 | -0.747490000 |

| | | | |
|---|---|---|---|
| C | 4.837620000 | -1.264221000 | -2.761546000 |
| C | 5.547383000 | -2.262437000 | -2.086777000 |
| H | 4.073018000 | -2.006728000 | 0.967359000 |
| H | 3.297319000 | 0.227453000 | -2.637853000 |
| H | 5.808412000 | -3.301686000 | -0.215523000 |
| H | 5.053562000 | -1.050818000 | -3.804710000 |
| H | 6.316515000 | -2.828951000 | -2.604119000 |
| C | 2.318170000 | -0.288597000 | 1.839848000 |
| C | 3.348612000 | 0.204466000 | 2.657467000 |
| C | 1.367327000 | -1.160071000 | 2.389784000 |
| C | 3.430665000 | -0.179637000 | 3.996029000 |
| C | 1.454579000 | -1.547037000 | 3.728828000 |
| C | 2.485334000 | -1.058189000 | 4.533026000 |
| H | 4.083831000 | 0.893245000 | 2.252429000 |
| H | 0.547495000 | -1.517565000 | 1.776684000 |
| H | 4.229967000 | 0.210684000 | 4.620057000 |
| H | 0.709138000 | -2.219630000 | 4.143662000 |
| H | 2.548675000 | -1.353282000 | 5.576851000 |
| Cl | -1.130484000 | 1.124866000 | 1.006386000 |

## Int1-C

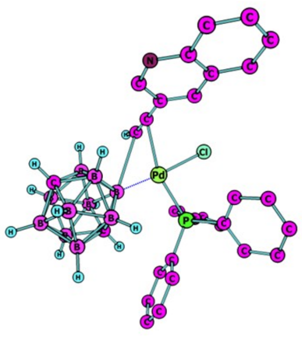

| Atom | x | y | z |
|---|---|---|---|
| Pd | 0.557740000 | -0.351279000 | -2.351488000 |
| P | 2.119378000 | 0.197608000 | -0.630937000 |
| C | -4.801763000 | 2.135998000 | -3.038809000 |
| C | -3.546254000 | 2.825166000 | -3.095571000 |
| C | -3.505913000 | 4.223801000 | -2.842221000 |
| C | -4.662788000 | 4.907545000 | -2.542288000 |
| C | -5.904065000 | 4.223859000 | -2.484202000 |
| C | -5.974836000 | 2.868319000 | -2.727064000 |
| N | -4.910647000 | 0.791793000 | -3.276462000 |
| C | -3.818355000 | 0.120657000 | -3.555713000 |
| C | -2.513476000 | 0.708108000 | -3.632057000 |
| C | -2.392035000 | 2.073463000 | -3.406023000 |
| C | -1.387452000 | -0.091291000 | -3.979689000 |
| C | -0.423947000 | -0.771368000 | -4.329797000 |
| H | 0.219572000 | -1.329900000 | -4.978469000 |
| H | -2.550067000 | 4.738760000 | -2.891756000 |
| H | -4.629948000 | 5.975860000 | -2.349483000 |
| H | -6.807168000 | 4.778843000 | -2.246250000 |
| H | -6.916217000 | 2.329425000 | -2.688202000 |
| H | -3.926277000 | -0.945323000 | -3.746236000 |
| H | -1.411690000 | 2.540689000 | -3.456360000 |
| H | -0.111939000 | -4.349520000 | 1.643271000 |
| H | 1.493455000 | -5.650996000 | -0.486394000 |
| H | 1.575481000 | -2.914597000 | -0.041531000 |
| H | 1.783273000 | -3.751031000 | -2.714867000 |

| | | | |
|---|---|---|---|
| B | -0.380856000 | -4.168742000 | 0.503840000 |
| B | 0.582388000 | -4.950601000 | -0.775346000 |
| C | 0.665422000 | -3.270241000 | -0.502894000 |
| B | 0.758468000 | -3.778087000 | -2.118544000 |
| B | -1.984589000 | -3.827185000 | -0.145296000 |
| H | -3.006774000 | -3.880486000 | 0.451528000 |
| B | -1.129814000 | -5.331668000 | -0.588845000 |
| H | -1.585658000 | -6.383091000 | -0.287363000 |
| C | -1.892963000 | -4.357005000 | -1.760901000 |
| H | -2.802313000 | -4.735158000 | -2.214587000 |
| B | -0.793110000 | -2.520789000 | -0.056869000 |
| H | -0.824911000 | -1.642529000 | 0.736874000 |
| B | -1.802192000 | -2.667820000 | -1.491059000 |
| H | -2.723725000 | -1.980244000 | -1.773413000 |
| B | -0.425923000 | -5.097381000 | -2.211667000 |
| H | -0.414713000 | -5.990403000 | -2.990433000 |
| B | -0.846615000 | -3.457406000 | -2.762698000 |
| H | -1.131998000 | -3.315620000 | -3.901233000 |
| B | -0.078626000 | -2.239860000 | -1.682672000 |
| C | 1.596400000 | 1.685715000 | 0.324614000 |
| C | 2.359147000 | 2.132564000 | 1.420800000 |
| C | 0.412681000 | 2.359599000 | -0.014481000 |
| C | 1.960100000 | 3.254429000 | 2.161448000 |
| C | -0.011202000 | 3.476758000 | 0.723003000 |
| C | 0.789863000 | 3.869775000 | 1.771326000 |
| H | 3.260976000 | 1.604263000 | 1.715877000 |

| | | | |
|---|---|---|---|
| H | -0.174520000 | 2.025568000 | -0.862600000 |
| H | 2.553044000 | 3.601279000 | 3.003054000 |
| H | -0.930265000 | 3.993137000 | 0.460898000 |
| C | 3.710985000 | 0.581581000 | -1.479935000 |
| C | 4.143010000 | -0.295241000 | -2.488699000 |
| C | 4.507838000 | 1.685301000 | -1.149869000 |
| C | 5.359382000 | -0.083365000 | -3.138171000 |
| C | 5.719346000 | 1.901273000 | -1.810323000 |
| C | 6.150602000 | 1.017164000 | -2.800360000 |
| H | 3.520175000 | -1.137477000 | -2.778799000 |
| H | 4.182740000 | 2.392250000 | -0.394928000 |
| H | 5.680398000 | -0.770348000 | -3.916175000 |
| H | 6.322176000 | 2.767408000 | -1.551818000 |
| H | 7.092945000 | 1.189136000 | -3.312915000 |
| C | 2.655992000 | -0.929794000 | 0.739504000 |
| C | 3.813334000 | -1.719022000 | 0.631564000 |
| C | 1.869973000 | -1.040212000 | 1.901456000 |
| C | 4.172556000 | -2.596547000 | 1.657776000 |
| C | 2.233556000 | -1.915726000 | 2.924814000 |
| C | 3.384833000 | -2.698412000 | 2.805465000 |
| H | 4.442732000 | -1.647201000 | -0.248714000 |
| H | 0.975530000 | -0.436663000 | 2.013917000 |
| H | 5.073365000 | -3.195539000 | 1.558089000 |
| H | 1.615619000 | -1.984779000 | 3.815530000 |
| H | 3.666348000 | -3.380306000 | 3.602803000 |
| Cl | 1.214061000 | 1.931766000 | -3.319672000 |

TS2-C

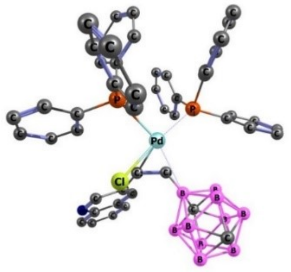

| Atom | x | y | z |
|---|---|---|---|
| Pd | -0.170508000 | 0.871300000 | -2.129057000 |
| P | 0.171041000 | -1.408279000 | -2.885498000 |
| C | 1.727172000 | -1.740607000 | -3.829346000 |
| C | 1.871559000 | -2.830544000 | -4.704792000 |
| H | 1.025749000 | -3.477171000 | -4.917968000 |
| C | 3.103603000 | -3.097033000 | -5.301579000 |
| H | 3.199902000 | -3.937329000 | -5.983852000 |
| C | 4.214318000 | -2.296608000 | -5.013534000 |
| H | 5.173959000 | -2.511207000 | -5.475806000 |
| C | 4.087349000 | -1.229823000 | -4.123299000 |
| H | 4.943972000 | -0.604781000 | -3.887220000 |
| C | 2.848743000 | -0.953383000 | -3.536900000 |
| H | -1.461337000 | -3.995455000 | -2.678770000 |
| C | 0.222820000 | -2.938372000 | -1.822381000 |
| C | -0.656126000 | -4.023187000 | -1.953811000 |
| H | 2.750835000 | -0.117852000 | -2.851386000 |
| C | -0.500687000 | -5.163274000 | -1.159519000 |

| | | | |
|---|---|---|---|
| H | -1.193690000 | -5.992513000 | -1.274763000 |
| C | 0.538741000 | -5.241913000 | -0.233011000 |
| H | 0.657701000 | -6.128974000 | 0.382931000 |
| C | 1.432189000 | -4.174336000 | -0.109176000 |
| H | 2.252570000 | -4.227437000 | 0.601144000 |
| C | 1.274019000 | -3.034188000 | -0.895141000 |
| H | 1.981227000 | -2.217193000 | -0.791420000 |
| C | -1.245381000 | -1.721681000 | -4.031807000 |
| C | -2.531601000 | -1.880023000 | -3.478899000 |
| H | -2.664835000 | -1.897550000 | -2.400755000 |
| C | -3.650180000 | -2.042189000 | -4.295112000 |
| H | -4.629945000 | -2.173662000 | -3.844051000 |
| C | -3.509627000 | -2.035369000 | -5.685570000 |
| H | -4.380107000 | -2.159129000 | -6.323717000 |
| C | -2.244495000 | -1.862914000 | -6.245433000 |
| H | -2.121335000 | -1.842488000 | -7.324285000 |
| C | -1.120707000 | -1.705458000 | -5.428495000 |
| H | -0.153019000 | -1.557114000 | -5.892071000 |
| C | -5.529701000 | 3.956210000 | -0.370433000 |
| C | -5.575445000 | 2.532414000 | -0.212622000 |
| C | -6.800026000 | 1.914550000 | 0.164120000 |
| C | -7.927324000 | 2.677159000 | 0.376897000 |
| C | -7.879771000 | 4.083914000 | 0.215078000 |
| C | -6.705336000 | 4.711089000 | -0.152547000 |
| N | -4.381603000 | 4.613853000 | -0.738271000 |
| C | -3.308563000 | 3.905019000 | -0.976138000 |

| | | | |
|---|---|---|---|
| C | -3.219330000 | 2.468854000 | -0.815972000 |
| C | -4.384320000 | 1.805720000 | -0.416750000 |
| C | -2.056644000 | 1.778565000 | -1.254681000 |
| C | -1.552492000 | 0.553914000 | -0.675975000 |
| H | -6.828936000 | 0.834768000 | 0.287290000 |
| H | -8.859209000 | 2.201634000 | 0.668573000 |
| H | -8.777782000 | 4.671420000 | 0.383553000 |
| H | -6.649310000 | 5.787499000 | -0.280253000 |
| H | -2.404883000 | 4.433429000 | -1.270645000 |
| H | -4.399511000 | 0.725372000 | -0.315987000 |
| P | 0.726744000 | 2.134027000 | -4.255188000 |
| C | -0.196161000 | 3.729121000 | -4.472766000 |
| C | -1.572984000 | 3.751406000 | -4.202591000 |
| H | -2.052510000 | 2.867823000 | -3.792870000 |
| C | -2.323757000 | 4.908312000 | -4.420065000 |
| H | -3.387846000 | 4.907705000 | -4.199775000 |
| C | -1.705061000 | 6.067731000 | -4.892812000 |
| H | -2.286225000 | 6.972267000 | -5.050444000 |
| C | -0.333254000 | 6.059830000 | -5.149401000 |
| H | 0.160707000 | 6.959457000 | -5.507000000 |
| C | 0.415852000 | 4.898380000 | -4.946312000 |
| H | 1.481096000 | 4.916039000 | -5.148852000 |
| C | 2.512971000 | 2.628709000 | -4.196861000 |
| C | 3.224935000 | 3.029138000 | -5.341691000 |
| H | 2.733960000 | 3.051710000 | -6.309197000 |
| C | 4.570982000 | 3.389774000 | -5.254869000 |

| | | | |
|---|---|---|---|
| H | 5.103533000 | 3.696828000 | -6.151088000 |
| C | 5.228993000 | 3.354557000 | -4.023398000 |
| H | 6.277362000 | 3.633061000 | -3.956699000 |
| C | 4.531463000 | 2.962816000 | -2.879235000 |
| H | 5.032246000 | 2.939132000 | -1.915118000 |
| C | 3.184167000 | 2.603448000 | -2.963602000 |
| H | 2.640410000 | 2.327487000 | -2.066473000 |
| C | 0.577367000 | 1.420941000 | -5.973768000 |
| C | 1.562994000 | 0.560483000 | -6.490419000 |
| H | 2.450511000 | 0.336873000 | -5.909709000 |
| C | 1.428221000 | -0.004689000 | -7.760154000 |
| H | 2.210022000 | -0.657820000 | -8.138778000 |
| C | 0.301433000 | 0.269363000 | -8.539127000 |
| H | 0.199968000 | -0.165877000 | -9.529728000 |
| C | -0.688105000 | 1.114704000 | -8.034829000 |
| H | -1.567643000 | 1.343255000 | -8.631049000 |
| C | -0.551516000 | 1.684498000 | -6.767051000 |
| H | -1.326638000 | 2.351599000 | -6.405935000 |
| Cl | 0.386902000 | 3.312205000 | -1.096532000 |
| H | -2.172761000 | -0.230508000 | -1.148648000 |
| H | -2.690206000 | -2.513470000 | 3.054442000 |
| H | -3.467054000 | 0.218152000 | 3.943097000 |
| H | -3.520170000 | -0.557879000 | 1.266881000 |
| H | -2.761077000 | 2.101779000 | 1.831228000 |
| B | -2.003312000 | -1.582383000 | 2.796218000 |
| B | -2.468002000 | 0.052525000 | 3.326930000 |

| | | | |
|---|---|---|---|
| C | -2.531745000 | -0.401624000 | 1.684203000 |
| B | -2.040736000 | 1.185498000 | 2.017085000 |
| B | -0.253780000 | -1.612423000 | 2.580488000 |
| H | 0.464257000 | -2.525617000 | 2.813446000 |
| B | -0.978781000 | -0.685135000 | 3.918726000 |
| H | -0.742123000 | -0.977717000 | 5.042199000 |
| C | 0.228507000 | -0.016339000 | 2.916783000 |
| H | 1.212414000 | 0.125726000 | 3.349378000 |
| B | -1.295943000 | -1.437002000 | 1.168540000 |
| H | -1.528798000 | -2.282602000 | 0.374218000 |
| B | 0.168769000 | -0.465987000 | 1.280152000 |
| H | 1.179000000 | -0.618644000 | 0.686675000 |
| B | -1.006177000 | 1.032814000 | 3.431991000 |
| H | -0.790405000 | 1.876384000 | 4.235448000 |
| B | -0.297393000 | 1.169014000 | 1.799227000 |
| H | 0.400031000 | 2.090604000 | 1.563421000 |
| B | -1.306921000 | 0.295512000 | 0.563660000 |

## Int1-RE-C

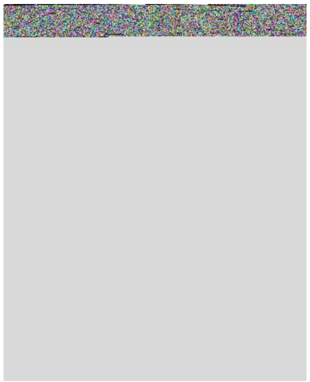

| **Atom** | **x** | **y** | **z** |
|---|---|---|---|

| | | | |
|---|---|---|---|
| Pd | -0.227488000 | 1.067218000 | -2.165900000 |
| P | -0.485003000 | -1.119155000 | -3.081454000 |
| C | 1.083576000 | -1.678245000 | -3.879368000 |
| C | 1.103237000 | -2.520155000 | -5.001931000 |
| C | 2.317513000 | -2.960351000 | -5.532381000 |
| C | 3.524849000 | -2.567029000 | -4.949463000 |
| C | 3.514819000 | -1.729547000 | -3.831432000 |
| C | 2.301564000 | -1.285553000 | -3.301387000 |
| C | -0.895550000 | -2.547783000 | -1.974457000 |
| C | -2.119361000 | -2.554327000 | -1.282399000 |
| C | -2.469091000 | -3.631002000 | -0.468682000 |
| C | -1.598502000 | -4.714197000 | -0.319098000 |
| C | -0.379792000 | -4.716886000 | -0.997074000 |
| C | -0.031017000 | -3.643822000 | -1.823583000 |
| C | -1.780680000 | -1.279032000 | -4.391429000 |
| C | -2.363535000 | -2.519338000 | -4.707356000 |
| C | -3.326003000 | -2.613510000 | -5.713562000 |
| C | -3.723381000 | -1.472957000 | -6.415757000 |
| C | -3.154618000 | -0.236009000 | -6.106617000 |
| C | -2.192761000 | -0.139949000 | -5.098856000 |
| C | -5.295736000 | 2.199693000 | -0.687825000 |
| C | -4.842680000 | 1.443039000 | -1.812695000 |
| C | -5.719603000 | 1.248081000 | -2.914085000 |
| C | -6.990876000 | 1.780054000 | -2.895854000 |
| C | -7.438956000 | 2.529319000 | -1.778807000 |
| C | -6.608741000 | 2.736619000 | -0.697570000 |

| | | | |
|---|---|---|---|
| N | -4.493718000 | 2.429357000 | 0.396319000 |
| C | -3.283481000 | 1.917687000 | 0.392431000 |
| C | -2.720043000 | 1.117741000 | -0.661811000 |
| C | -3.526522000 | 0.917371000 | -1.769765000 |
| C | -1.339897000 | 0.598752000 | -0.506584000 |
| C | -0.931953000 | 0.101846000 | 0.676547000 |
| P | 0.900182000 | 2.306814000 | -4.162986000 |
| C | 0.138513000 | 3.988084000 | -4.273125000 |
| C | 0.865688000 | 5.120174000 | -4.666440000 |
| C | 0.233980000 | 6.360226000 | -4.785626000 |
| C | -1.131757000 | 6.481904000 | -4.522763000 |
| C | -1.864447000 | 5.359040000 | -4.130912000 |
| C | -1.231391000 | 4.122633000 | -3.997855000 |
| C | 2.711470000 | 2.610735000 | -3.945219000 |
| C | 3.562281000 | 2.907492000 | -5.024329000 |
| C | 4.921767000 | 3.143782000 | -4.811784000 |
| C | 5.451287000 | 3.087580000 | -3.520567000 |
| C | 4.613616000 | 2.797594000 | -2.441917000 |
| C | 3.253436000 | 2.559466000 | -2.650453000 |
| C | 0.792606000 | 1.740730000 | -5.930513000 |
| C | -0.109650000 | 2.322668000 | -6.836650000 |
| C | -0.210768000 | 1.849986000 | -8.147469000 |
| C | 0.590490000 | 0.791972000 | -8.578662000 |
| C | 1.494143000 | 0.208010000 | -7.688117000 |
| C | 1.591750000 | 0.673586000 | -6.376392000 |
| B | 0.452027000 | -0.288053000 | 1.306229000 |

| | | | |
|---|---|---|---|
| Cl | 0.154230000 | 3.149789000 | -0.952642000 |
| B | 3.148546000 | -1.377464000 | 1.100437000 |
| B | 2.200395000 | -2.486660000 | 2.126066000 |
| C | 1.477686000 | -1.603132000 | 0.850565000 |
| B | 0.555085000 | -1.817317000 | 2.261266000 |
| B | 3.251692000 | 0.181606000 | 1.925651000 |
| B | 3.323398000 | -1.337099000 | 2.858475000 |
| C | 2.324977000 | -0.038093000 | 3.337425000 |
| B | 2.085943000 | -0.029950000 | 0.615662000 |
| B | 1.602821000 | 0.848401000 | 2.064618000 |
| B | 1.713946000 | -1.611940000 | 3.577423000 |
| B | 0.656303000 | -0.263382000 | 3.083602000 |

## TS1-RE-C

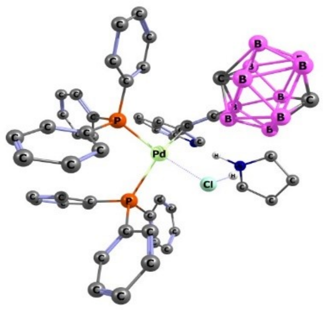

| Atom | x | y | z |
|---|---|---|---|
| Pd | -0.042429000 | 1.073554000 | -2.132170000 |

| | | | |
|---|---|---|---|
| P | -0.270243000 | -1.140763000 | -3.023823000 |
| C | 1.273371000 | -1.564714000 | -3.947842000 |
| C | 1.265146000 | -2.378326000 | -5.092340000 |
| H | 0.328513000 | -2.757674000 | -5.486929000 |
| C | 2.460442000 | -2.700451000 | -5.737880000 |
| H | 2.439012000 | -3.328965000 | -6.624023000 |
| C | 3.676836000 | -2.215261000 | -5.250632000 |
| H | 4.605217000 | -2.463837000 | -5.757366000 |
| C | 3.694463000 | -1.406231000 | -4.112415000 |
| H | 4.633597000 | -1.015566000 | -3.731680000 |
| C | 2.499995000 | -1.081201000 | -3.466241000 |
| H | -2.384668000 | -1.909650000 | -1.124440000 |
| C | -0.487068000 | -2.603046000 | -1.907229000 |
| C | -1.647058000 | -2.703464000 | -1.118131000 |
| H | 2.519271000 | -0.440141000 | -2.590562000 |
| C | -1.862934000 | -3.820276000 | -0.310816000 |
| H | -2.766992000 | -3.880491000 | 0.288322000 |
| C | -0.918519000 | -4.850043000 | -0.264625000 |
| H | -1.085355000 | -5.716164000 | 0.369383000 |
| C | 0.241888000 | -4.755340000 | -1.034121000 |
| H | 0.984554000 | -5.547683000 | -1.004326000 |
| C | 0.456039000 | -3.641854000 | -1.852801000 |
| H | 1.359510000 | -3.590800000 | -2.449526000 |
| C | -1.625115000 | -1.410296000 | -4.253990000 |
| C | -2.162678000 | -2.684107000 | -4.508965000 |
| H | -1.803788000 | -3.548556000 | -3.960026000 |

| | | | |
|---|---|---|---|
| C | -3.167727000 | -2.849128000 | -5.463898000 |
| H | -3.575764000 | -3.839228000 | -5.647772000 |
| C | -3.648519000 | -1.747809000 | -6.176325000 |
| H | -4.432820000 | -1.878204000 | -6.916887000 |
| C | -3.121894000 | -0.478494000 | -5.928354000 |
| H | -3.491429000 | 0.385226000 | -6.472949000 |
| C | -2.121762000 | -0.310818000 | -4.969696000 |
| H | -1.731218000 | 0.680201000 | -4.769848000 |
| C | -5.010878000 | 2.470940000 | -1.501863000 |
| C | -4.683049000 | 1.232768000 | -2.132317000 |
| C | -5.619732000 | 0.622925000 | -3.009572000 |
| C | -6.840505000 | 1.216823000 | -3.252046000 |
| C | -7.169985000 | 2.445468000 | -2.626504000 |
| C | -6.275273000 | 3.057723000 | -1.772950000 |
| N | -4.147071000 | 3.115749000 | -0.652014000 |
| C | -2.958876000 | 2.589808000 | -0.349758000 |
| C | -2.562670000 | 1.308748000 | -0.945292000 |
| C | -3.414304000 | 0.674449000 | -1.835503000 |
| C | -1.266361000 | 0.712270000 | -0.536709000 |
| C | -1.004769000 | 0.281411000 | 0.707800000 |
| H | -5.357412000 | -0.319060000 | -3.486966000 |
| H | -7.554146000 | 0.745332000 | -3.922696000 |
| H | -8.135542000 | 2.904590000 | -2.824096000 |
| H | -6.507860000 | 3.999511000 | -1.283190000 |
| H | -3.144033000 | -0.261248000 | -2.316813000 |
| P | 0.943029000 | 2.396103000 | -4.194229000 |

| | | | |
|---|---|---|---|
| C | 0.253114000 | 4.100897000 | -3.990452000 |
| C | 0.960995000 | 5.261778000 | -4.333929000 |
| H | 1.973961000 | 5.190242000 | -4.716462000 |
| C | 0.374884000 | 6.520088000 | -4.177715000 |
| H | 0.938588000 | 7.410647000 | -4.442805000 |
| C | -0.926192000 | 6.635513000 | -3.683311000 |
| H | -1.379617000 | 7.615829000 | -3.563848000 |
| C | -1.639668000 | 5.485172000 | -3.336686000 |
| H | -2.647683000 | 5.559284000 | -2.938320000 |
| C | -1.050241000 | 4.227896000 | -3.481904000 |
| H | -1.608229000 | 3.344731000 | -3.183082000 |
| C | 2.772490000 | 2.633624000 | -4.356433000 |
| C | 3.364421000 | 3.066486000 | -5.556365000 |
| H | 2.748393000 | 3.261760000 | -6.428275000 |
| C | 4.746706000 | 3.240151000 | -5.645572000 |
| H | 5.186608000 | 3.575988000 | -6.580839000 |
| C | 5.559403000 | 2.981796000 | -4.539238000 |
| H | 6.635628000 | 3.114931000 | -4.610039000 |
| C | 4.981861000 | 2.551876000 | -3.343101000 |
| H | 5.605851000 | 2.351416000 | -2.476240000 |
| C | 3.599113000 | 2.376965000 | -3.250949000 |
| H | 3.156666000 | 2.062511000 | -2.312173000 |
| C | 0.458531000 | 1.982582000 | -5.937782000 |
| C | -0.563234000 | 2.667796000 | -6.613735000 |
| H | -1.062178000 | 3.506531000 | -6.140050000 |
| C | -0.944174000 | 2.287646000 | -7.903855000 |

| | | | |
|---|---|---|---|
| H | -1.733393000 | 2.835902000 | -8.411585000 |
| C | -0.308930000 | 1.220889000 | -8.540720000 |
| H | -0.602632000 | 0.929096000 | -9.545330000 |
| C | 0.711325000 | 0.533381000 | -7.877960000 |
| H | 1.216350000 | -0.297076000 | -8.363959000 |
| C | 1.088856000 | 0.906557000 | -6.588214000 |
| H | 1.889835000 | 0.365605000 | -6.094629000 |
| H | 2.180522000 | -0.326506000 | -0.217426000 |
| B | 0.242576000 | -0.261678000 | 1.498532000 |
| Cl | 1.168422000 | 2.827547000 | -0.765636000 |
| H | -1.885294000 | 0.360326000 | 1.359507000 |
| H | 3.269944000 | -2.737889000 | 1.067006000 |
| H | 1.201868000 | -3.827225000 | 2.897921000 |
| H | 0.534667000 | -2.467877000 | 0.545697000 |
| H | -1.168350000 | -2.057744000 | 2.728278000 |
| B | 2.583339000 | -2.001533000 | 1.692557000 |
| B | 1.344830000 | -2.655308000 | 2.794762000 |
| C | 0.923971000 | -1.852634000 | 1.345114000 |
| B | -0.079820000 | -1.591482000 | 2.686005000 |
| B | 3.044402000 | -0.398510000 | 2.270067000 |
| H | 4.131515000 | 0.071515000 | 2.230316000 |
| B | 2.690818000 | -1.706931000 | 3.431586000 |
| H | 3.543030000 | -2.097115000 | 4.156541000 |
| C | 2.034909000 | -0.143955000 | 3.619200000 |
| H | 2.427250000 | 0.467171000 | 4.424293000 |
| B | 1.916207000 | -0.538866000 | 0.914392000 |

| | | | |
|---|---|---|---|
| B | 1.615967000 | 0.662896000 | 2.171305000 |
| H | 1.771673000 | 1.830655000 | 2.089416000 |
| B | 1.035137000 | -1.455615000 | 4.048156000 |
| H | 0.784845000 | -1.678137000 | 5.184954000 |
| B | 0.375932000 | 0.005847000 | 3.265400000 |
| H | -0.308726000 | 0.741454000 | 3.894505000 |
| H | -0.154184000 | 3.719026000 | 0.379567000 |
| H | -0.229577000 | 3.118051000 | 2.636151000 |
| C | -0.715703000 | 4.063088000 | 2.389809000 |
| C | 0.118352000 | 5.312179000 | 2.750396000 |
| H | -1.697234000 | 4.083780000 | 2.877718000 |
| C | -1.087797000 | 5.588370000 | 0.622267000 |
| C | -0.115827000 | 6.310658000 | 1.579057000 |
| H | 1.179088000 | 5.053996000 | 2.826801000 |
| H | -0.186895000 | 5.724844000 | 3.716883000 |
| H | -0.884430000 | 5.762731000 | -0.436241000 |
| H | -2.129004000 | 5.865481000 | 0.822745000 |
| H | 0.827514000 | 6.529452000 | 1.069409000 |
| H | -0.531058000 | 7.263853000 | 1.920301000 |
| N | -0.939350000 | 4.145056000 | 0.928142000 |
| H | -1.873564000 | 3.481448000 | 0.515498000 |

# Int2-RE-C

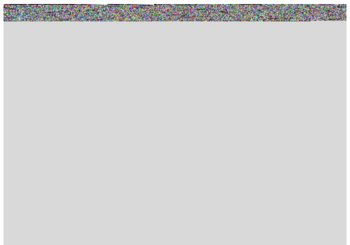

| Atom | x | y | z |
|---|---|---|---|
| Pd | 2.700852000 | -1.306725000 | 0.017222000 |
| P | 0.823429000 | -2.646671000 | -0.373900000 |
| P | 4.728294000 | -0.238066000 | 0.410274000 |
| C | 0.799656000 | -4.162445000 | 0.686744000 |
| C | 1.291689000 | -4.047556000 | 1.997810000 |
| H | 1.689532000 | -3.093064000 | 2.334136000 |
| C | 1.287243000 | -5.145988000 | 2.858059000 |
| H | 1.670277000 | -5.040426000 | 3.869558000 |
| C | 0.805188000 | -6.380369000 | 2.414840000 |
| H | 0.810146000 | -7.239075000 | 3.080845000 |
| C | 0.325510000 | -6.508859000 | 1.109841000 |
| H | -0.044696000 | -7.467838000 | 0.756856000 |
| C | 0.320085000 | -5.407121000 | 0.250464000 |
| H | -0.053711000 | -5.521219000 | -0.762535000 |
| C | -0.850853000 | -1.906537000 | -0.102778000 |
| C | -1.919674000 | -2.599007000 | 0.486253000 |
| H | -1.784649000 | -3.622095000 | 0.823155000 |
| C | -3.162387000 | -1.979947000 | 0.647182000 |
| H | -3.979708000 | -2.528025000 | 1.108936000 |
| C | -3.356003000 | -0.665782000 | 0.218170000 |
| H | -4.324402000 | -0.187855000 | 0.340070000 |

| | | | |
|---|---|---|---|
| C | -2.296218000 | 0.033775000 | -0.366459000 |
| H | -2.438178000 | 1.060081000 | -0.693942000 |
| C | -1.052375000 | -0.579150000 | -0.516933000 |
| H | -0.224450000 | -0.028115000 | -0.956767000 |
| C | 0.711412000 | -3.317781000 | -2.094895000 |
| C | 1.913970000 | -3.564144000 | -2.778377000 |
| H | 2.857891000 | -3.332464000 | -2.290378000 |
| C | 1.902723000 | -4.086581000 | -4.072131000 |
| H | 2.841558000 | -4.272843000 | -4.586906000 |
| C | 0.687885000 | -4.356273000 | -4.707793000 |
| H | 0.677681000 | -4.754458000 | -5.718888000 |
| C | -0.513401000 | -4.103716000 | -4.042333000 |
| H | -1.461705000 | -4.305273000 | -4.533640000 |
| C | -0.503401000 | -3.589275000 | -2.743251000 |
| H | -1.444501000 | -3.394438000 | -2.237816000 |
| C | 5.959597000 | -1.262140000 | 1.336214000 |
| C | 5.971581000 | -2.644255000 | 1.083001000 |
| H | 5.250847000 | -3.063098000 | 0.384897000 |
| C | 6.885424000 | -3.476995000 | 1.729006000 |
| H | 6.882898000 | -4.543658000 | 1.521410000 |
| C | 7.790140000 | -2.942880000 | 2.650456000 |
| H | 8.495770000 | -3.592332000 | 3.161473000 |
| C | 7.777212000 | -1.573172000 | 2.919953000 |
| H | 8.472566000 | -1.152481000 | 3.641506000 |
| C | 6.869160000 | -0.735902000 | 2.266332000 |
| H | 6.867830000 | 0.326532000 | 2.490223000 |

| | | | |
|---|---|---|---|
| C | 5.653627000 | 0.269415000 | -1.110983000 |
| C | 7.052268000 | 0.373054000 | -1.172343000 |
| H | 7.653856000 | 0.129866000 | -0.301419000 |
| C | 7.682338000 | 0.775606000 | -2.352518000 |
| H | 8.766102000 | 0.848393000 | -2.385843000 |
| C | 6.925278000 | 1.077697000 | -3.486590000 |
| H | 7.418106000 | 1.386584000 | -4.404394000 |
| C | 5.533280000 | 0.965911000 | -3.441142000 |
| H | 4.939526000 | 1.179246000 | -4.326104000 |
| C | 4.903363000 | 0.557320000 | -2.263708000 |
| H | 3.822929000 | 0.433167000 | -2.234492000 |
| C | 4.646042000 | 1.325108000 | 1.404881000 |
| C | 5.505820000 | 2.423136000 | 1.221810000 |
| H | 6.276287000 | 2.388824000 | 0.457420000 |
| C | 5.372743000 | 3.570019000 | 2.012596000 |
| H | 6.034576000 | 4.416054000 | 1.850368000 |
| C | 4.383238000 | 3.636433000 | 2.996731000 |
| H | 4.266743000 | 4.536814000 | 3.591253000 |
| C | 3.526601000 | 2.548868000 | 3.190369000 |
| H | 2.747995000 | 2.598107000 | 3.946249000 |
| C | 3.651579000 | 1.406921000 | 2.396845000 |
| H | 2.967282000 | 0.572232000 | 2.529964000 |
| C | -3.570728000 | 4.756309000 | -0.876625000 |
| C | -3.537190000 | 4.139336000 | 0.416452000 |
| C | -2.512575000 | 4.501990000 | 1.333584000 |
| C | -1.559573000 | 5.432505000 | 0.981715000 |

| | | | |
|---|---|---|---|
| C | -1.595162000 | 6.042919000 | -0.299297000 |
| C | -2.579003000 | 5.715518000 | -1.208307000 |
| N | -4.526684000 | 4.448446000 | -1.807040000 |
| C | -5.434343000 | 3.553669000 | -1.493114000 |
| C | -5.494996000 | 2.868109000 | -0.235831000 |
| C | -4.532877000 | 3.180514000 | 0.713985000 |
| C | -6.509924000 | 1.896676000 | 0.000437000 |
| C | -7.379059000 | 1.061936000 | 0.182121000 |
| H | -2.494745000 | 4.030923000 | 2.313589000 |
| H | -0.761556000 | 5.707195000 | 1.664978000 |
| H | -0.828484000 | 6.770779000 | -0.547332000 |
| H | -2.626713000 | 6.174352000 | -2.191241000 |
| H | -6.184879000 | 3.323864000 | -2.248092000 |
| H | -4.539099000 | 2.689517000 | 1.683265000 |
| H | -8.662559000 | -3.871207000 | 0.017382000 |
| H | -10.417118000 | -2.457480000 | -1.913231000 |
| H | -7.833940000 | -1.536699000 | -1.307430000 |
| H | -9.775192000 | 0.431313000 | -1.782922000 |
| B | -9.108075000 | -2.808772000 | 0.291110000 |
| B | -10.163034000 | -1.958176000 | -0.870114000 |
| C | -8.582858000 | -1.409751000 | -0.533647000 |
| B | -9.779402000 | -0.218346000 | -0.793813000 |
| B | -9.493773000 | -2.228077000 | 1.914806000 |
| H | -9.492391000 | -2.874382000 | 2.906913000 |
| B | -10.788396000 | -2.454235000 | 0.706741000 |
| H | -11.643259000 | -3.249747000 | 0.901533000 |

| | | | |
|---|---|---|---|
| C | -10.687101000 | -1.035679000 | 1.653232000 |
| H | -11.432952000 | -0.902655000 | 2.428898000 |
| B | -8.071597000 | -1.594656000 | 1.085145000 |
| H | -6.942742000 | -1.850609000 | 1.334047000 |
| B | -9.108639000 | -0.487471000 | 1.993669000 |
| H | -8.852302000 | 0.016418000 | 3.033464000 |
| B | -11.201959000 | -0.852680000 | 0.035875000 |
| H | -12.329356000 | -0.590625000 | -0.213267000 |
| B | -10.166975000 | 0.364329000 | 0.830483000 |
| H | -10.606024000 | 1.428297000 | 1.105558000 |
| B | -8.472212000 | 0.025960000 | 0.416416000 |
| Cl | 2.058891000 | 6.432257000 | 1.089320000 |
| H | 3.328899000 | 3.293567000 | 0.295618000 |
| C | 1.313270000 | 3.006779000 | -0.318966000 |
| C | 3.043711000 | 4.186227000 | -1.596602000 |
| C | 0.941946000 | 3.043438000 | -1.813541000 |
| H | 1.486646000 | 1.998334000 | 0.060880000 |
| H | 0.575801000 | 3.510983000 | 0.308176000 |
| C | 1.729631000 | 4.241602000 | -2.374051000 |
| H | 3.576434000 | 5.136558000 | -1.533278000 |
| H | 3.711939000 | 3.406782000 | -1.973122000 |
| H | -0.136669000 | 3.148961000 | -1.954737000 |
| H | 1.259279000 | 2.116257000 | -2.304037000 |
| H | 1.211286000 | 5.180347000 | -2.152040000 |
| H | 1.890026000 | 4.182618000 | -3.453812000 |
| N | 2.599146000 | 3.803437000 | -0.210093000 |

| | | | |
|---|---|---|---|
| H | 2.411142000 | 4.721785000 | 0.335933000 |

# TS1-DC

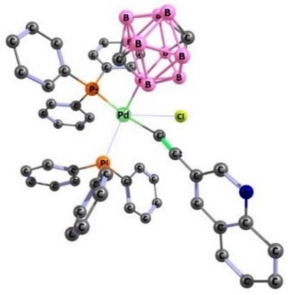

| Atom | x | y | z |
|---|---|---|---|
| Pd | 0.398022000 | 0.282459000 | -0.737810000 |
| Cl | 0.310241000 | 0.234979000 | -4.468455000 |
| P | -1.158532000 | 2.370584000 | -0.065439000 |
| P | 2.539189000 | 0.993860000 | 0.097876000 |
| C | -6.267322000 | -1.764756000 | -3.116432000 |
| C | -5.985619000 | -0.360078000 | -3.131314000 |
| C | -7.023379000 | 0.553763000 | -3.461906000 |
| C | -8.285707000 | 0.093471000 | -3.763775000 |
| C | -8.563037000 | -1.296996000 | -3.748109000 |
| C | -7.576587000 | -2.207368000 | -3.431710000 |
| N | -5.314286000 | -2.699711000 | -2.809303000 |
| C | -4.104996000 | -2.278861000 | -2.523601000 |
| C | -3.707958000 | -0.898666000 | -2.500836000 |

| | | | |
|---|---|---|---|
| C | -4.673558000 | 0.053039000 | -2.809530000 |
| C | -2.375154000 | -0.543198000 | -2.175575000 |
| C | -1.190735000 | -0.251824000 | -1.954018000 |
| H | -0.464648000 | -0.038228000 | -3.123727000 |
| H | -6.801119000 | 1.617789000 | -3.472431000 |
| H | -9.075683000 | 0.794902000 | -4.016452000 |
| H | -9.563921000 | -1.644138000 | -3.989121000 |
| H | -7.769275000 | -3.275541000 | -3.415568000 |
| H | -3.359275000 | -3.035999000 | -2.287167000 |
| H | -4.419992000 | 1.109259000 | -2.806370000 |
| H | 0.085206000 | -4.534350000 | 1.947071000 |
| H | 3.014835000 | -4.148887000 | 1.594152000 |
| H | 1.229339000 | -2.003688000 | 1.608861000 |
| H | 3.437631000 | -1.840052000 | -0.122208000 |
| B | 0.519800000 | -4.184829000 | 0.901088000 |
| B | 2.272550000 | -3.956118000 | 0.690551000 |
| C | 1.197596000 | -2.625439000 | 0.721665000 |
| B | 2.505737000 | -2.524269000 | -0.349387000 |
| B | -0.181167000 | -4.524361000 | -0.680843000 |
| H | -1.085440000 | -5.258215000 | -0.901173000 |
| B | 1.418901000 | -5.179798000 | -0.247126000 |
| H | 1.583419000 | -6.351744000 | -0.182188000 |
| C | 1.138051000 | -4.420314000 | -1.747955000 |
| H | 1.114462000 | -5.048046000 | -2.631635000 |
| B | -0.310278000 | -2.891106000 | -0.010470000 |
| H | -1.302940000 | -2.445107000 | 0.453572000 |

| | | | |
|---|---|---|---|
| B | 0.065038000 | -3.094894000 | -1.721613000 |
| H | -0.660743000 | -2.940390000 | -2.639082000 |
| B | 2.649861000 | -4.148212000 | -1.022321000 |
| H | 3.635673000 | -4.631594000 | -1.468350000 |
| B | 1.810077000 | -2.853114000 | -1.929687000 |
| H | 2.246041000 | -2.530446000 | -2.980591000 |
| B | 0.907891000 | -1.805369000 | -0.791418000 |
| C | 2.763676000 | 2.822707000 | 0.290858000 |
| C | 3.290770000 | 3.412510000 | 1.446641000 |
| C | 2.513684000 | 3.627478000 | -0.833233000 |
| C | 3.575225000 | 4.781203000 | 1.472192000 |
| C | 2.800634000 | 4.990638000 | -0.805605000 |
| C | 3.336590000 | 5.571030000 | 0.347847000 |
| H | 3.493777000 | 2.809247000 | 2.325315000 |
| H | 2.111932000 | 3.183500000 | -1.740398000 |
| H | 3.988586000 | 5.225314000 | 2.373769000 |
| H | 2.608612000 | 5.598854000 | -1.685183000 |
| H | 3.564557000 | 6.633133000 | 0.368352000 |
| C | 3.931196000 | 0.703347000 | -1.084903000 |
| C | 3.657430000 | 0.168295000 | -2.350602000 |
| C | 5.239136000 | 1.133416000 | -0.795303000 |
| C | 4.677520000 | 0.029187000 | -3.295823000 |
| C | 6.258064000 | 0.977735000 | -1.734282000 |
| C | 5.979957000 | 0.420416000 | -2.986134000 |
| H | 2.645355000 | -0.125073000 | -2.607893000 |
| H | 5.461994000 | 1.613080000 | 0.152914000 |

| | | | |
|---|---|---|---|
| H | 4.445060000 | -0.382762000 | -4.273608000 |
| H | 7.265608000 | 1.305674000 | -1.493421000 |
| H | 6.773466000 | 0.307920000 | -3.719854000 |
| C | 3.010666000 | 0.320073000 | 1.751844000 |
| C | 4.256850000 | -0.243202000 | 2.062431000 |
| C | 2.022287000 | 0.361870000 | 2.752054000 |
| C | 4.511736000 | -0.737331000 | 3.344517000 |
| C | 2.281663000 | -0.122142000 | 4.034919000 |
| C | 3.530053000 | -0.673734000 | 4.334366000 |
| H | 5.029426000 | -0.314792000 | 1.305737000 |
| H | 1.039617000 | 0.764893000 | 2.522306000 |
| H | 5.480618000 | -1.176863000 | 3.564378000 |
| H | 1.505847000 | -0.077912000 | 4.794179000 |
| H | 3.731721000 | -1.059660000 | 5.329590000 |
| C | -0.725220000 | 3.897138000 | 0.901995000 |
| C | -0.344457000 | 3.758976000 | 2.248636000 |
| C | -0.791701000 | 5.186806000 | 0.355424000 |
| C | -0.064222000 | 4.877915000 | 3.031135000 |
| C | -0.507374000 | 6.308309000 | 1.139996000 |
| C | -0.150106000 | 6.159411000 | 2.479977000 |
| H | -0.291623000 | 2.772232000 | 2.700741000 |
| H | -1.080730000 | 5.324105000 | -0.681018000 |
| H | 0.216481000 | 4.748809000 | 4.073081000 |
| H | -0.574921000 | 7.299653000 | 0.699695000 |
| H | 0.061904000 | 7.032648000 | 3.090887000 |
| C | -2.515970000 | 1.709045000 | 1.017439000 |

| | | | |
|---|---|---|---|
| C | -3.394722000 | 2.561465000 | 1.712090000 |
| C | -2.663250000 | 0.323267000 | 1.179152000 |
| C | -4.404530000 | 2.039367000 | 2.520902000 |
| C | -3.669913000 | -0.198871000 | 1.996026000 |
| C | -4.546195000 | 0.656588000 | 2.664271000 |
| H | -3.282878000 | 3.638314000 | 1.636268000 |
| H | -1.990386000 | -0.350416000 | 0.662270000 |
| H | -5.075856000 | 2.714548000 | 3.044780000 |
| H | -3.764014000 | -1.275613000 | 2.108103000 |
| H | -5.329630000 | 0.251091000 | 3.298739000 |
| C | -2.005588000 | 3.077255000 | -1.550885000 |
| C | -3.322924000 | 3.563202000 | -1.538784000 |
| C | -1.268405000 | 3.152007000 | -2.742381000 |
| C | -3.882930000 | 4.120108000 | -2.690725000 |
| C | -1.826441000 | 3.715199000 | -3.892000000 |
| C | -3.134652000 | 4.201373000 | -3.868571000 |
| H | -3.920405000 | 3.502264000 | -0.635439000 |
| H | -0.264375000 | 2.741565000 | -2.786646000 |
| H | -4.904143000 | 4.491648000 | -2.665853000 |
| H | -1.242638000 | 3.752676000 | -4.807006000 |
| H | -3.572892000 | 4.632952000 | -4.764338000 |

# Int1-DC

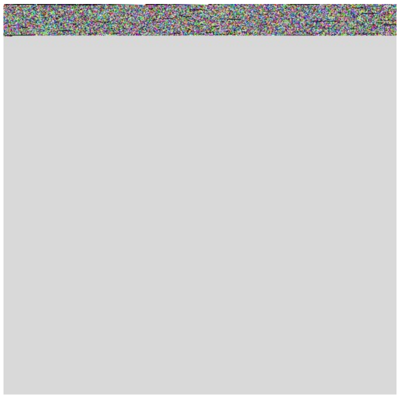

| Atom | x | y | z |
|---|---|---|---|
| Pd | 0.318919000 | 0.218426000 | -0.532970000 |
| Cl | 0.393366000 | 0.747816000 | -3.980906000 |
| P | -1.202066000 | 2.314963000 | 0.343557000 |
| P | 2.533185000 | 0.943196000 | 0.094434000 |
| C | -6.107929000 | -1.585452000 | -3.825239000 |
| C | -5.755867000 | -0.197651000 | -3.771807000 |
| C | -6.657160000 | 0.772205000 | -4.287525000 |
| C | -7.859232000 | 0.379810000 | -4.832723000 |
| C | -8.207741000 | -0.993795000 | -4.884462000 |
| C | -7.352809000 | -1.957234000 | -4.392542000 |
| N | -5.285622000 | -2.570234000 | -3.346076000 |
| C | -4.139635000 | -2.217816000 | -2.813656000 |
| C | -3.689244000 | -0.863237000 | -2.718116000 |
| C | -4.504503000 | 0.143900000 | -3.209862000 |
| C | -2.436095000 | -0.547597000 | -2.094824000 |
| C | -1.377522000 | -0.253264000 | -1.494705000 |
| H | -1.306067000 | -0.935988000 | -2.561879000 |
| H | -6.379801000 | 1.822073000 | -4.244157000 |

| | | | |
|---|---|---|---|
| H | -8.546746000 | 1.122249000 | -5.227231000 |
| H | -9.159943000 | -1.285796000 | -5.318218000 |
| H | -7.602363000 | -3.013090000 | -4.424118000 |
| H | -3.505516000 | -3.014216000 | -2.428344000 |
| H | -4.188218000 | 1.181620000 | -3.164524000 |
| H | 0.106763000 | -4.519210000 | 2.340860000 |
| H | 3.018242000 | -4.138221000 | 1.857800000 |
| H | 1.226170000 | -2.006563000 | 1.878754000 |
| H | 3.368792000 | -1.897118000 | 0.048517000 |
| B | 0.499712000 | -4.209251000 | 1.266511000 |
| B | 2.239680000 | -3.982999000 | 0.978233000 |
| C | 1.162731000 | -2.653152000 | 1.011552000 |
| B | 2.431354000 | -2.590183000 | -0.120896000 |
| B | -0.262194000 | -4.607281000 | -0.270112000 |
| H | -1.175204000 | -5.344043000 | -0.435409000 |
| B | 1.355454000 | -5.241859000 | 0.118680000 |
| H | 1.526192000 | -6.410384000 | 0.214557000 |
| C | 1.014056000 | -4.537328000 | -1.388811000 |
| H | 0.954807000 | -5.200616000 | -2.245014000 |
| B | -0.369592000 | -2.950784000 | 0.344150000 |
| H | -1.349241000 | -2.488915000 | 0.819382000 |
| B | -0.053906000 | -3.218238000 | -1.364827000 |
| H | -0.817451000 | -3.109890000 | -2.262366000 |
| B | 2.552085000 | -4.237054000 | -0.740731000 |
| H | 3.520468000 | -4.731953000 | -1.209864000 |
| B | 1.675543000 | -2.974263000 | -1.661099000 |

| | | | |
|---|---|---|---|
| H | 2.058830000 | -2.684753000 | -2.741263000 |
| B | 0.816844000 | -1.885658000 | -0.524832000 |
| C | 2.672355000 | 2.786359000 | 0.181171000 |
| C | 3.196101000 | 3.472777000 | 1.282868000 |
| C | 2.335700000 | 3.501310000 | -0.982978000 |
| C | 3.395571000 | 4.855476000 | 1.217883000 |
| C | 2.535779000 | 4.879330000 | -1.040004000 |
| C | 3.070239000 | 5.559309000 | 0.058906000 |
| H | 3.461165000 | 2.936827000 | 2.188551000 |
| H | 1.944257000 | 2.978148000 | -1.853761000 |
| H | 3.807441000 | 5.377950000 | 2.077021000 |
| H | 2.280521000 | 5.420547000 | -1.946915000 |
| H | 3.230859000 | 6.632863000 | 0.010022000 |
| C | 3.858461000 | 0.644626000 | -1.157368000 |
| C | 3.537664000 | 0.012611000 | -2.364455000 |
| C | 5.153673000 | 1.166012000 | -0.977256000 |
| C | 4.505251000 | -0.127080000 | -3.363511000 |
| C | 6.119457000 | 1.009494000 | -1.969958000 |
| C | 5.797288000 | 0.358011000 | -3.165159000 |
| H | 2.528534000 | -0.344039000 | -2.539349000 |
| H | 5.405370000 | 1.718186000 | -0.076900000 |
| H | 4.235447000 | -0.607752000 | -4.299323000 |
| H | 7.117817000 | 1.410081000 | -1.816516000 |
| H | 6.548628000 | 0.245145000 | -3.942116000 |
| C | 3.120974000 | 0.336567000 | 1.734814000 |
| C | 4.395197000 | -0.196948000 | 1.978816000 |

| | | | |
|---|---|---|---|
| C | 2.196418000 | 0.382745000 | 2.793432000 |
| C | 4.740556000 | -0.650956000 | 3.254408000 |
| C | 2.546199000 | -0.060595000 | 4.069406000 |
| C | 3.822745000 | -0.578403000 | 4.303152000 |
| H | 5.118561000 | -0.276107000 | 1.175965000 |
| H | 1.193387000 | 0.758974000 | 2.613947000 |
| H | 5.730123000 | -1.066309000 | 3.423084000 |
| H | 1.818980000 | -0.010868000 | 4.875067000 |
| H | 4.095280000 | -0.932193000 | 5.293449000 |
| C | -0.678758000 | 3.873168000 | 1.213655000 |
| C | -0.161376000 | 3.779857000 | 2.517712000 |
| C | -0.807198000 | 5.143766000 | 0.634091000 |
| C | 0.191291000 | 4.924229000 | 3.231261000 |
| C | -0.448831000 | 6.290124000 | 1.348999000 |
| C | 0.044687000 | 6.186164000 | 2.649520000 |
| H | -0.060253000 | 2.809268000 | 2.994992000 |
| H | -1.198649000 | 5.246650000 | -0.371932000 |
| H | 0.577079000 | 4.829510000 | 4.242825000 |
| H | -0.564468000 | 7.266096000 | 0.885248000 |
| H | 0.314415000 | 7.079477000 | 3.206237000 |
| C | -2.410766000 | 1.654484000 | 1.588723000 |
| C | -3.233123000 | 2.511557000 | 2.345290000 |
| C | -2.513745000 | 0.272799000 | 1.801501000 |
| C | -4.140368000 | 1.995430000 | 3.270552000 |
| C | -3.419379000 | -0.244258000 | 2.732180000 |
| C | -4.236807000 | 0.614649000 | 3.466626000 |

| | | | |
|---|---|---|---|
| H | -3.161113000 | 3.586919000 | 2.218134000 |
| H | -1.878999000 | -0.404639000 | 1.242569000 |
| H | -4.768944000 | 2.673459000 | 3.841541000 |
| H | -3.478497000 | -1.318878000 | 2.882255000 |
| H | -4.940366000 | 0.213948000 | 4.191236000 |
| C | -2.247432000 | 2.944566000 | -1.043213000 |
| C | -3.630711000 | 3.166671000 | -0.949875000 |
| C | -1.597082000 | 3.199456000 | -2.260543000 |
| C | -4.338520000 | 3.660851000 | -2.049031000 |
| C | -2.303136000 | 3.692683000 | -3.356492000 |
| C | -3.676449000 | 3.931223000 | -3.249872000 |
| H | -4.160616000 | 2.953845000 | -0.027618000 |
| H | -0.535640000 | 2.947776000 | -2.373827000 |
| H | -5.407976000 | 3.835904000 | -1.964857000 |
| H | -1.784467000 | 3.869142000 | -4.293584000 |
| H | -4.230739000 | 4.313654000 | -4.103170000 |

## TS2-DC

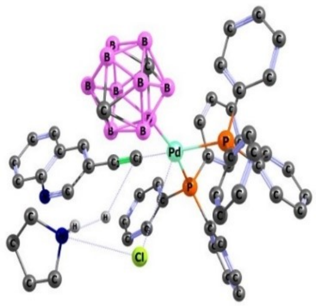


| Atom | x | y | z |
|---|---|---|---|
| Pd | 2.609651000 | -0.265799000 | -0.550663000 |
| P | 4.926445000 | -0.882633000 | -1.201953000 |

| | | | |
|---|---|---|---|
| C | 5.950960000 | 0.211193000 | -2.311610000 |
| C | 6.309655000 | -0.150277000 | -3.618742000 |
| H | 5.974709000 | -1.092062000 | -4.038414000 |
| C | 7.110708000 | 0.691336000 | -4.395436000 |
| H | 7.381003000 | 0.390025000 | -5.403992000 |
| C | 7.565658000 | 1.904837000 | -3.879779000 |
| H | 8.190132000 | 2.557246000 | -4.483957000 |
| C | 7.216573000 | 2.272591000 | -2.578128000 |
| H | 7.565634000 | 3.213384000 | -2.161976000 |
| C | 6.418675000 | 1.433408000 | -1.800689000 |
| H | 4.612200000 | -0.356335000 | 1.631263000 |
| C | 6.103898000 | -0.995635000 | 0.221266000 |
| C | 5.649010000 | -0.652233000 | 1.502113000 |
| H | 6.172122000 | 1.729830000 | -0.786571000 |
| C | 6.517253000 | -0.662352000 | 2.596991000 |
| H | 6.147345000 | -0.390047000 | 3.581758000 |
| C | 7.856117000 | -1.014475000 | 2.422563000 |
| H | 8.532790000 | -1.026721000 | 3.272501000 |
| C | 8.328840000 | -1.331498000 | 1.145937000 |
| H | 9.375260000 | -1.584305000 | 0.998974000 |
| C | 7.464298000 | -1.308328000 | 0.051472000 |
| H | 7.858285000 | -1.512104000 | -0.939252000 |
| C | 4.970776000 | -2.472485000 | -2.145779000 |
| C | 5.925690000 | -3.484963000 | -1.975147000 |
| H | 6.677954000 | -3.406278000 | -1.198567000 |
| C | 5.908235000 | -4.619620000 | -2.789102000 |

| | | | |
|---|---|---|---|
| H | 6.649269000 | -5.399416000 | -2.635785000 |
| C | 4.947048000 | -4.753343000 | -3.793153000 |
| H | 4.939681000 | -5.634819000 | -4.428265000 |
| C | 3.987349000 | -3.754552000 | -3.967286000 |
| H | 3.223222000 | -3.855365000 | -4.732861000 |
| C | 3.990634000 | -2.630082000 | -3.139898000 |
| H | 3.213297000 | -1.879515000 | -3.259061000 |
| H | -0.373866000 | -2.582645000 | -0.173564000 |
| H | 0.660207000 | -1.210698000 | 2.474930000 |
| H | 3.373769000 | -1.879831000 | 2.605684000 |
| H | 4.494215000 | -3.290462000 | 0.472664000 |
| B | 0.545522000 | -2.977899000 | 0.461140000 |
| B | 1.075734000 | -2.189311000 | 1.961034000 |
| C | 2.723978000 | -2.571640000 | 2.085113000 |
| B | 3.360343000 | -3.425141000 | 0.766312000 |
| B | 0.817140000 | -4.722219000 | 0.646748000 |
| H | 0.129290000 | -5.592429000 | 0.224223000 |
| B | 0.256374000 | -3.761884000 | 2.031600000 |
| H | -0.791865000 | -3.982309000 | 2.540207000 |
| C | 1.531501000 | -4.897500000 | 2.187944000 |
| H | 1.339826000 | -5.802913000 | 2.752562000 |
| B | 1.970227000 | -3.741295000 | -0.286537000 |
| H | 2.079006000 | -3.894051000 | -1.457612000 |
| B | 2.561548000 | -5.003663000 | 0.825424000 |
| H | 3.047046000 | -6.045370000 | 0.532594000 |
| B | 1.649627000 | -3.451193000 | 3.058087000 |

| | | | |
|---|---|---|---|
| H | 1.629105000 | -3.384740000 | 4.238956000 |
| B | 3.072550000 | -4.220275000 | 2.316218000 |
| H | 3.916455000 | -4.618081000 | 3.043363000 |
| B | 2.137834000 | -2.118449000 | 0.482055000 |
| C | -4.284161000 | 1.518410000 | 1.500813000 |
| C | -3.199227000 | 2.058979000 | 2.261903000 |
| C | -3.488345000 | 2.911130000 | 3.362766000 |
| C | -4.792040000 | 3.212224000 | 3.692123000 |
| C | -5.865004000 | 2.675756000 | 2.936613000 |
| C | -5.615629000 | 1.846355000 | 1.863479000 |
| N | -4.075357000 | 0.689426000 | 0.431611000 |
| C | -2.837147000 | 0.390583000 | 0.105037000 |
| C | -1.670550000 | 0.874909000 | 0.789304000 |
| C | -1.882470000 | 1.714794000 | 1.874953000 |
| C | -0.372523000 | 0.488352000 | 0.355901000 |
| C | 0.754404000 | 0.145414000 | 0.008204000 |
| H | -2.662551000 | 3.320434000 | 3.940173000 |
| H | -5.003095000 | 3.863976000 | 4.535688000 |
| H | -6.887817000 | 2.921679000 | 3.208855000 |
| H | -6.421057000 | 1.422488000 | 1.270918000 |
| H | -2.701361000 | -0.285621000 | -0.738652000 |
| H | -1.037118000 | 2.112208000 | 2.430516000 |
| P | 2.581911000 | 2.220771000 | -1.493829000 |
| C | 1.001150000 | 3.170086000 | -1.293563000 |
| C | 0.846280000 | 4.213185000 | -0.371455000 |
| H | 1.667008000 | 4.494111000 | 0.279782000 |

| | | | |
|---|---|---|---|
| C | -0.363013000 | 4.911187000 | -0.286378000 |
| H | -0.464196000 | 5.720794000 | 0.431727000 |
| C | -1.429056000 | 4.576109000 | -1.120164000 |
| H | -2.367549000 | 5.119665000 | -1.054092000 |
| C | -1.284272000 | 3.532530000 | -2.039590000 |
| H | -2.110758000 | 3.259587000 | -2.690060000 |
| C | -0.081981000 | 2.833101000 | -2.123222000 |
| H | 0.013137000 | 2.022201000 | -2.839601000 |
| C | 3.766802000 | 3.217904000 | -0.479977000 |
| C | 4.565205000 | 4.259046000 | -0.976053000 |
| H | 4.530954000 | 4.520185000 | -2.027874000 |
| C | 5.427392000 | 4.958711000 | -0.127990000 |
| H | 6.041141000 | 5.760251000 | -0.530542000 |
| C | 5.501679000 | 4.633112000 | 1.227965000 |
| H | 6.172513000 | 5.179535000 | 1.885229000 |
| C | 4.711043000 | 3.598368000 | 1.733761000 |
| H | 4.762871000 | 3.335328000 | 2.786799000 |
| C | 3.856373000 | 2.892044000 | 0.885227000 |
| H | 3.252622000 | 2.080289000 | 1.283325000 |
| C | 2.934705000 | 2.608275000 | -3.268245000 |
| C | 2.772289000 | 3.901581000 | -3.799940000 |
| H | 2.454212000 | 4.717239000 | -3.157614000 |
| C | 2.990189000 | 4.144527000 | -5.155436000 |
| H | 2.864020000 | 5.149343000 | -5.549466000 |
| C | 3.352644000 | 3.096281000 | -6.007077000 |
| H | 3.512770000 | 3.284999000 | -7.065156000 |

| | | | |
|---|---|---|---|
| C | 3.492211000 | 1.806210000 | -5.496320000 |
| H | 3.758313000 | 0.983214000 | -6.153624000 |
| C | 3.282716000 | 1.565286000 | -4.135695000 |
| H | 3.378894000 | 0.557535000 | -3.748064000 |
| Cl | -0.033233000 | -0.945911000 | -3.617749000 |
| H | -1.889493000 | -3.190021000 | -2.048259000 |
| C | -3.677557000 | -3.071930000 | -1.013908000 |
| C | -3.451546000 | -2.619426000 | -3.287317000 |
| C | -4.991194000 | -2.356588000 | -1.404322000 |
| H | -3.813488000 | -4.167141000 | -1.064692000 |
| H | -3.337920000 | -2.823825000 | -0.003162000 |
| C | -4.807226000 | -1.994545000 | -2.907453000 |
| H | -2.914727000 | -2.060178000 | -4.060094000 |
| H | -3.604181000 | -3.649631000 | -3.658874000 |
| H | -5.130566000 | -1.452681000 | -0.804829000 |
| H | -5.856378000 | -3.005907000 | -1.236301000 |
| H | -4.771211000 | -0.907727000 | -3.034365000 |
| H | -5.617106000 | -2.373254000 | -3.538865000 |
| N | -2.712090000 | -2.590860000 | -2.016618000 |
| H | -0.496087000 | -0.964497000 | -2.403983000 |

Int1-RE

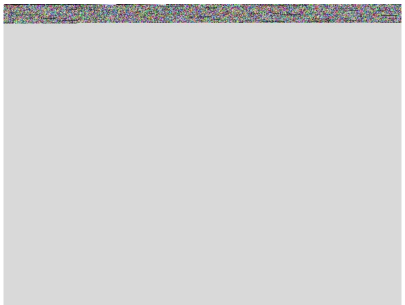

| Atom | x | y | z |
|---|---|---|---|
| Pd | 1.541070000 | 0.079847000 | 0.394433000 |
| P | 3.972226000 | 0.063871000 | 0.817008000 |
| C | 4.888576000 | 0.343795000 | -0.762154000 |
| C | 6.284555000 | 0.505887000 | -0.824531000 |
| H | 6.879888000 | 0.477046000 | 0.082501000 |
| C | 6.924534000 | 0.675103000 | -2.052318000 |
| H | 8.002638000 | 0.806514000 | -2.082966000 |
| C | 6.185591000 | 0.659440000 | -3.239270000 |
| H | 6.687965000 | 0.783299000 | -4.194686000 |
| C | 4.804314000 | 0.466100000 | -3.193207000 |
| H | 4.223438000 | 0.434389000 | -4.110633000 |
| C | 4.161461000 | 0.309112000 | -1.962029000 |
| H | 2.570364000 | 0.931430000 | 3.145590000 |
| C | 4.300056000 | 1.474889000 | 1.968343000 |
| C | 3.422329000 | 1.601597000 | 3.059891000 |
| H | 3.086566000 | 0.153760000 | -1.929305000 |
| C | 3.635406000 | 2.575049000 | 4.037940000 |
| H | 2.952037000 | 2.651530000 | 4.879199000 |
| C | 4.719600000 | 3.448208000 | 3.929308000 |
| H | 4.885771000 | 4.209185000 | 4.686837000 |

| | | | |
|---|---|---|---|
| C | 5.582317000 | 3.345542000 | 2.836555000 |
| H | 6.418176000 | 4.032032000 | 2.735451000 |
| C | 5.378165000 | 2.364316000 | 1.864128000 |
| H | 6.053202000 | 2.312908000 | 1.018322000 |
| C | 5.026525000 | -1.265318000 | 1.584462000 |
| C | 5.286889000 | -1.290934000 | 2.963552000 |
| H | 4.885758000 | -0.518304000 | 3.610345000 |
| C | 6.070981000 | -2.302152000 | 3.522693000 |
| H | 6.263753000 | -2.299519000 | 4.592055000 |
| C | 6.607519000 | -3.305765000 | 2.714631000 |
| H | 7.220260000 | -4.090406000 | 3.149918000 |
| C | 6.355787000 | -3.290513000 | 1.341204000 |
| H | 6.771466000 | -4.063206000 | 0.700147000 |
| C | 5.572384000 | -2.280316000 | 0.781142000 |
| H | 5.396756000 | -2.281409000 | -0.289358000 |
| H | -1.464735000 | -4.708943000 | -0.284169000 |
| H | -1.922533000 | -3.900868000 | 2.538674000 |
| H | -1.310568000 | -2.036346000 | 0.541182000 |
| H | -0.267298000 | -1.537008000 | 3.096409000 |
| B | -0.564167000 | -4.309824000 | 0.375611000 |
| B | -0.838616000 | -3.825992000 | 2.064793000 |
| C | -0.498878000 | -2.677340000 | 0.855465000 |
| B | 0.167112000 | -2.377596000 | 2.382584000 |
| B | 1.128135000 | -4.765412000 | 0.199988000 |
| H | 1.554279000 | -5.628204000 | -0.492839000 |
| B | 0.234632000 | -5.176790000 | 1.689028000 |

| | | | |
|---|---|---|---|
| H | 0.065803000 | -6.312010000 | 1.986372000 |
| C | 1.790580000 | -4.488154000 | 1.744090000 |
| H | 2.609596000 | -5.124926000 | 2.059342000 |
| B | 0.606728000 | -3.155398000 | -0.333854000 |
| H | 0.472643000 | -2.844989000 | -1.470190000 |
| B | 2.127530000 | -3.328675000 | 0.531103000 |
| H | 3.206016000 | -3.284722000 | 0.051414000 |
| B | 0.684549000 | -3.981446000 | 2.936462000 |
| H | 0.815849000 | -4.324546000 | 4.063763000 |
| B | 1.855418000 | -2.845002000 | 2.220773000 |
| H | 2.752142000 | -2.473920000 | 2.895625000 |
| B | 1.090381000 | -1.924564000 | 0.897697000 |
| C | -5.730448000 | 1.045993000 | -0.704985000 |
| C | -5.106208000 | 0.048292000 | -1.519492000 |
| C | -5.893909000 | -0.659389000 | -2.467960000 |
| C | -7.238461000 | -0.389161000 | -2.601226000 |
| C | -7.854942000 | 0.599592000 | -1.793807000 |
| C | -7.116260000 | 1.302337000 | -0.865340000 |
| N | -5.030904000 | 1.763718000 | 0.227858000 |
| C | -3.748558000 | 1.515625000 | 0.367302000 |
| C | -3.012338000 | 0.539930000 | -0.388081000 |
| C | -3.722874000 | -0.186210000 | -1.334543000 |
| C | -1.621036000 | 0.348990000 | -0.149557000 |
| C | -0.420280000 | 0.202681000 | 0.068204000 |
| H | -5.417552000 | -1.417806000 | -3.084829000 |
| H | -7.833338000 | -0.935474000 | -3.328083000 |

| | | | |
|---|---|---|---|
| H | -8.916291000 | 0.801016000 | -1.910427000 |
| H | -7.566814000 | 2.063574000 | -0.235422000 |
| H | -3.212389000 | 2.100323000 | 1.113047000 |
| H | -3.219482000 | -0.938393000 | -1.936583000 |
| P | 1.489989000 | 2.636659000 | -0.432071000 |
| C | 2.849283000 | 3.903927000 | -0.337526000 |
| C | 3.935524000 | 3.822618000 | -1.227667000 |
| H | 3.966976000 | 3.040754000 | -1.979112000 |
| C | 4.972762000 | 4.753788000 | -1.176857000 |
| H | 5.795858000 | 4.676823000 | -1.882461000 |
| C | 4.951091000 | 5.781288000 | -0.230169000 |
| H | 5.756707000 | 6.509769000 | -0.193780000 |
| C | 3.884130000 | 5.866230000 | 0.664377000 |
| H | 3.852819000 | 6.662539000 | 1.403393000 |
| C | 2.841967000 | 4.936828000 | 0.612349000 |
| H | 2.018503000 | 5.028338000 | 1.312144000 |
| C | 0.125043000 | 3.486397000 | 0.483102000 |
| C | 0.006528000 | 3.237227000 | 1.859983000 |
| H | 0.678403000 | 2.531501000 | 2.339185000 |
| C | -0.973456000 | 3.879511000 | 2.618627000 |
| H | -1.049544000 | 3.676718000 | 3.683493000 |
| C | -1.862447000 | 4.767178000 | 2.006851000 |
| H | -2.632796000 | 5.259952000 | 2.593589000 |
| C | -1.761535000 | 5.010357000 | 0.635589000 |
| H | -2.455348000 | 5.691264000 | 0.150113000 |
| C | -0.772136000 | 4.378241000 | -0.121465000 |

| | | | |
|---|---|---|---|
| H | -0.711604000 | 4.576747000 | -1.186398000 |
| C | 1.022362000 | 2.749928000 | -2.221951000 |
| C | 0.541423000 | 1.607058000 | -2.880592000 |
| H | 0.425060000 | 0.681241000 | -2.327720000 |
| C | 0.189708000 | 1.659231000 | -4.231644000 |
| H | -0.179927000 | 0.764023000 | -4.724447000 |
| C | 0.312516000 | 2.852714000 | -4.944729000 |
| H | 0.039780000 | 2.892204000 | -5.995901000 |
| C | 0.790242000 | 3.996747000 | -4.300796000 |
| H | 0.889764000 | 4.930457000 | -4.847842000 |
| C | 1.145780000 | 3.945845000 | -2.952106000 |
| H | 1.530218000 | 4.839319000 | -2.470686000 |

# TS-RE1

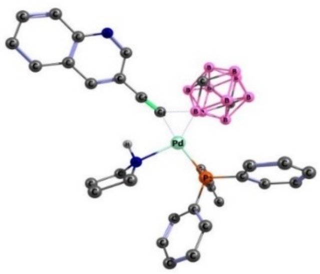


| Atom | x | y | z |
|---|---|---|---|
| Pd | 0.424975000 | 0.214512000 | 0.170273000 |
| P | -0.579228000 | -2.143250000 | 0.639082000 |
| P | 2.734757000 | -0.261674000 | -0.624493000 |
| C | 0.331059000 | -3.743490000 | 0.429974000 |
| C | 1.568040000 | -3.900670000 | 1.080570000 |
| H | 1.956462000 | -3.103644000 | 1.707277000 |

| | | | |
|---|---|---|---|
| C | 2.307342000 | -5.074499000 | 0.940300000 |
| H | 3.256325000 | -5.176692000 | 1.460053000 |
| C | 1.833724000 | -6.109535000 | 0.129318000 |
| H | 2.411093000 | -7.022951000 | 0.014831000 |
| C | 0.615536000 | -5.959742000 | -0.534134000 |
| H | 0.237819000 | -6.757672000 | -1.168145000 |
| C | -0.132864000 | -4.788856000 | -0.383191000 |
| H | -1.083392000 | -4.697249000 | -0.897604000 |
| C | -1.078595000 | -2.186893000 | 2.425770000 |
| C | -0.954179000 | -3.325407000 | 3.238102000 |
| H | -0.541375000 | -4.242956000 | 2.832228000 |
| C | -1.356882000 | -3.291805000 | 4.576085000 |
| H | -1.250840000 | -4.182447000 | 5.189998000 |
| C | -1.895673000 | -2.125003000 | 5.120322000 |
| H | -2.207814000 | -2.100443000 | 6.160917000 |
| C | -2.031105000 | -0.988642000 | 4.318724000 |
| H | -2.450702000 | -0.075410000 | 4.732148000 |
| C | -1.621908000 | -1.016338000 | 2.984948000 |
| H | -1.728394000 | -0.127677000 | 2.370324000 |
| C | -2.174802000 | -2.462163000 | -0.238661000 |
| C | -2.336176000 | -1.959227000 | -1.538482000 |
| H | -1.545019000 | -1.365446000 | -1.984858000 |
| C | -3.510575000 | -2.199890000 | -2.254476000 |
| H | -3.621395000 | -1.801113000 | -3.259212000 |
| C | -4.544945000 | -2.936763000 | -1.674221000 |
| H | -5.463094000 | -3.116571000 | -2.226901000 |

| | | | |
|---|---|---|---|
| C | -4.399101000 | -3.434391000 | -0.376818000 |
| H | -5.202571000 | -4.003970000 | 0.082532000 |
| C | -3.222194000 | -3.199752000 | 0.336385000 |
| H | -3.120708000 | -3.590226000 | 1.344481000 |
| C | 2.654992000 | -1.647488000 | -1.856069000 |
| C | 1.540520000 | -1.670525000 | -2.713131000 |
| H | 0.764734000 | -0.916619000 | -2.604007000 |
| C | 1.410182000 | -2.653306000 | -3.695522000 |
| H | 0.543797000 | -2.647955000 | -4.351371000 |
| C | 2.383024000 | -3.646747000 | -3.822437000 |
| H | 2.280843000 | -4.418804000 | -4.580092000 |
| C | 3.481400000 | -3.649111000 | -2.961003000 |
| H | 4.236721000 | -4.426117000 | -3.043131000 |
| C | 3.619453000 | -2.656780000 | -1.987491000 |
| H | 4.476809000 | -2.686010000 | -1.324735000 |
| C | 3.586195000 | 1.064572000 | -1.616163000 |
| C | 3.189327000 | 1.311685000 | -2.941421000 |
| H | 2.441218000 | 0.683779000 | -3.414013000 |
| C | 3.755700000 | 2.353501000 | -3.676705000 |
| H | 3.436724000 | 2.519344000 | -4.702268000 |
| C | 4.728174000 | 3.174975000 | -3.102116000 |
| H | 5.169477000 | 3.985681000 | -3.675213000 |
| C | 5.128220000 | 2.943473000 | -1.785807000 |
| H | 5.885005000 | 3.573043000 | -1.325455000 |
| C | 4.562090000 | 1.900340000 | -1.048656000 |
| H | 4.893870000 | 1.738859000 | -0.029192000 |

| | | | |
|---|---|---|---|
| C | 4.015257000 | -0.751478000 | 0.611365000 |
| C | 5.372596000 | -0.943618000 | 0.295821000 |
| H | 5.724438000 | -0.781950000 | -0.718582000 |
| C | 6.284872000 | -1.323362000 | 1.281339000 |
| H | 7.329327000 | -1.469729000 | 1.019854000 |
| C | 5.859267000 | -1.507632000 | 2.599839000 |
| H | 6.571974000 | -1.799741000 | 3.366204000 |
| C | 4.518671000 | -1.303501000 | 2.930353000 |
| H | 4.182042000 | -1.430799000 | 3.955506000 |
| C | 3.605245000 | -0.924819000 | 1.942944000 |
| H | 2.565105000 | -0.751523000 | 2.206761000 |
| C | -6.436943000 | 2.330090000 | -1.233088000 |
| C | -5.461294000 | 2.440905000 | -2.275256000 |
| C | -5.881483000 | 2.843775000 | -3.572505000 |
| C | -7.207397000 | 3.122378000 | -3.822854000 |
| C | -8.171761000 | 3.011215000 | -2.789691000 |
| C | -7.793993000 | 2.623115000 | -1.521332000 |
| N | -6.100889000 | 1.946876000 | 0.038139000 |
| C | -4.842356000 | 1.675236000 | 0.291670000 |
| C | -3.773686000 | 1.751486000 | -0.667511000 |
| C | -4.116147000 | 2.141593000 | -1.957538000 |
| C | -2.445504000 | 1.432352000 | -0.283836000 |
| C | -1.299659000 | 1.157309000 | 0.080357000 |
| H | -5.138734000 | 2.928315000 | -4.362305000 |
| H | -7.519763000 | 3.429745000 | -4.817149000 |
| H | -9.213428000 | 3.234434000 | -3.003183000 |

| | | | |
|---|---|---|---|
| H | -8.514733000 | 2.531154000 | -0.714376000 |
| H | -4.599573000 | 1.369431000 | 1.308684000 |
| H | -3.351033000 | 2.216415000 | -2.726018000 |
| H | -1.540390000 | 5.666828000 | 1.914093000 |
| H | 0.433959000 | 6.089431000 | -0.263357000 |
| H | -1.266709000 | 3.867388000 | -0.205344000 |
| H | 1.326012000 | 3.454692000 | -1.216545000 |
| B | -0.593573000 | 4.959916000 | 1.824614000 |
| B | 0.587114000 | 5.208627000 | 0.514746000 |
| C | -0.460208000 | 3.863863000 | 0.517056000 |
| B | 1.114066000 | 3.599868000 | -0.061062000 |
| B | 0.218582000 | 4.135029000 | 3.151690000 |
| H | 0.013666000 | 4.303428000 | 4.306990000 |
| B | 1.074816000 | 5.384452000 | 2.205262000 |
| H | 1.441308000 | 6.380221000 | 2.733247000 |
| C | 1.795278000 | 3.886165000 | 2.575282000 |
| H | 2.597820000 | 3.879683000 | 3.304533000 |
| B | -0.796031000 | 3.201086000 | 2.049892000 |
| H | -1.869376000 | 2.797048000 | 2.333828000 |
| B | 0.738816000 | 2.526683000 | 2.567591000 |
| H | 0.902501000 | 1.659805000 | 3.358958000 |
| B | 2.132214000 | 4.546587000 | 1.042435000 |
| H | 3.206191000 | 4.981555000 | 0.793636000 |
| B | 1.928971000 | 2.791560000 | 1.278479000 |
| H | 2.889846000 | 2.109129000 | 1.229759000 |
| B | 0.247188000 | 2.302173000 | 0.850402000 |

Int2-RE

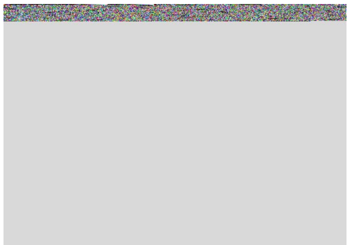

| Atom | x | y | z |
|---|---|---|---|
| Pd | 2.700852000 | -1.306725000 | 0.017222000 |
| P | 0.823429000 | -2.646671000 | -0.373900000 |
| P | 4.728294000 | -0.238066000 | 0.410274000 |
| C | 0.799656000 | -4.162445000 | 0.686744000 |
| C | 1.291689000 | -4.047556000 | 1.997810000 |
| H | 1.689532000 | -3.093064000 | 2.334136000 |
| C | 1.287243000 | -5.145988000 | 2.858059000 |
| H | 1.670277000 | -5.040426000 | 3.869558000 |
| C | 0.805188000 | -6.380369000 | 2.414840000 |
| H | 0.810146000 | -7.239075000 | 3.080845000 |
| C | 0.325510000 | -6.508859000 | 1.109841000 |
| H | -0.044696000 | -7.467838000 | 0.756856000 |
| C | 0.320085000 | -5.407121000 | 0.250464000 |
| H | -0.053711000 | -5.521219000 | -0.762535000 |
| C | -0.850853000 | -1.906537000 | -0.102778000 |
| C | -1.919674000 | -2.599007000 | 0.486253000 |
| H | -1.784649000 | -3.622095000 | 0.823155000 |
| C | -3.162387000 | -1.979947000 | 0.647182000 |

| | | | |
|---|---|---|---|
| H | -3.979708000 | -2.528025000 | 1.108936000 |
| C | -3.356003000 | -0.665782000 | 0.218170000 |
| H | -4.324402000 | -0.187855000 | 0.340070000 |
| C | -2.296218000 | 0.033775000 | -0.366459000 |
| H | -2.438178000 | 1.060081000 | -0.693942000 |
| C | -1.052375000 | -0.579150000 | -0.516933000 |
| H | -0.224450000 | -0.028115000 | -0.956767000 |
| C | 0.711412000 | -3.317781000 | -2.094895000 |
| C | 1.913970000 | -3.564144000 | -2.778377000 |
| H | 2.857891000 | -3.332464000 | -2.290378000 |
| C | 1.902723000 | -4.086581000 | -4.072131000 |
| H | 2.841558000 | -4.272843000 | -4.586906000 |
| C | 0.687885000 | -4.356273000 | -4.707793000 |
| H | 0.677681000 | -4.754458000 | -5.718888000 |
| C | -0.513401000 | -4.103716000 | -4.042333000 |
| H | -1.461705000 | -4.305273000 | -4.533640000 |
| C | -0.503401000 | -3.589275000 | -2.743251000 |
| H | -1.444501000 | -3.394438000 | -2.237816000 |
| C | 5.959597000 | -1.262140000 | 1.336214000 |
| C | 5.971581000 | -2.644255000 | 1.083001000 |
| H | 5.250847000 | -3.063098000 | 0.384897000 |
| C | 6.885424000 | -3.476995000 | 1.729006000 |
| H | 6.882898000 | -4.543658000 | 1.521410000 |
| C | 7.790140000 | -2.942880000 | 2.650456000 |
| H | 8.495770000 | -3.592332000 | 3.161473000 |
| C | 7.777212000 | -1.573172000 | 2.919953000 |

| | | | |
|---|---|---|---|
| H | 8.472566000 | -1.152481000 | 3.641506000 |
| C | 6.869160000 | -0.735902000 | 2.266332000 |
| H | 6.867830000 | 0.326532000 | 2.490223000 |
| C | 5.653627000 | 0.269415000 | -1.110983000 |
| C | 7.052268000 | 0.373054000 | -1.172343000 |
| H | 7.653856000 | 0.129866000 | -0.301419000 |
| C | 7.682338000 | 0.775606000 | -2.352518000 |
| H | 8.766102000 | 0.848393000 | -2.385843000 |
| C | 6.925278000 | 1.077697000 | -3.486590000 |
| H | 7.418106000 | 1.386584000 | -4.404394000 |
| C | 5.533280000 | 0.965911000 | -3.441142000 |
| H | 4.939526000 | 1.179246000 | -4.326104000 |
| C | 4.903363000 | 0.557320000 | -2.263708000 |
| H | 3.822929000 | 0.433167000 | -2.234492000 |
| C | 4.646042000 | 1.325108000 | 1.404881000 |
| C | 5.505820000 | 2.423136000 | 1.221810000 |
| H | 6.276287000 | 2.388824000 | 0.457420000 |
| C | 5.372743000 | 3.570019000 | 2.012596000 |
| H | 6.034576000 | 4.416054000 | 1.850368000 |
| C | 4.383238000 | 3.636433000 | 2.996731000 |
| H | 4.266743000 | 4.536814000 | 3.591253000 |
| C | 3.526601000 | 2.548868000 | 3.190369000 |
| H | 2.747995000 | 2.598107000 | 3.946249000 |
| C | 3.651579000 | 1.406921000 | 2.396845000 |
| H | 2.967282000 | 0.572232000 | 2.529964000 |
| C | -3.570728000 | 4.756309000 | -0.876625000 |

| | | | |
|---|---|---|---|
| C | -3.537190000 | 4.139336000 | 0.416452000 |
| C | -2.512575000 | 4.501990000 | 1.333584000 |
| C | -1.559573000 | 5.432505000 | 0.981715000 |
| C | -1.595162000 | 6.042919000 | -0.299297000 |
| C | -2.579003000 | 5.715518000 | -1.208307000 |
| N | -4.526684000 | 4.448446000 | -1.807040000 |
| C | -5.434343000 | 3.553669000 | -1.493114000 |
| C | -5.494996000 | 2.868109000 | -0.235831000 |
| C | -4.532877000 | 3.180514000 | 0.713985000 |
| C | -6.509924000 | 1.896676000 | 0.000437000 |
| C | -7.379059000 | 1.061936000 | 0.182121000 |
| H | -2.494745000 | 4.030923000 | 2.313589000 |
| H | -0.761556000 | 5.707195000 | 1.664978000 |
| H | -0.828484000 | 6.770779000 | -0.547332000 |
| H | -2.626713000 | 6.174352000 | -2.191241000 |
| H | -6.184879000 | 3.323864000 | -2.248092000 |
| H | -4.539099000 | 2.689517000 | 1.683265000 |
| H | -8.662559000 | -3.871207000 | 0.017382000 |
| H | -10.417118000 | -2.457480000 | -1.913231000 |
| H | -7.833940000 | -1.536699000 | -1.307430000 |
| H | -9.775192000 | 0.431313000 | -1.782922000 |
| B | -9.108075000 | -2.808772000 | 0.291110000 |
| B | -10.163034000 | -1.958176000 | -0.870114000 |
| C | -8.582858000 | -1.409751000 | -0.533647000 |
| B | -9.779402000 | -0.218346000 | -0.793813000 |
| B | -9.493773000 | -2.228077000 | 1.914806000 |

| | | | |
|---|---|---|---|
| H | -9.492391000 | -2.874382000 | 2.906913000 |
| B | -10.788396000 | -2.454235000 | 0.706741000 |
| H | -11.643259000 | -3.249747000 | 0.901533000 |
| C | -10.687101000 | -1.035679000 | 1.653232000 |
| H | -11.432952000 | -0.902655000 | 2.428898000 |
| B | -8.071597000 | -1.594656000 | 1.085145000 |
| H | -6.942742000 | -1.850609000 | 1.334047000 |
| B | -9.108639000 | -0.487471000 | 1.993669000 |
| H | -8.852302000 | 0.016418000 | 3.033464000 |
| B | -11.201959000 | -0.852680000 | 0.035875000 |
| H | -12.329356000 | -0.590625000 | -0.213267000 |
| B | -10.166975000 | 0.364329000 | 0.830483000 |
| H | -10.606024000 | 1.428297000 | 1.105558000 |
| B | -8.472212000 | 0.025960000 | 0.416416000 |
| Cl | 2.058891000 | 6.432257000 | 1.089320000 |
| H | 3.328899000 | 3.293567000 | 0.295618000 |
| C | 1.313270000 | 3.006779000 | -0.318966000 |
| C | 3.043711000 | 4.186227000 | -1.596602000 |
| C | 0.941946000 | 3.043438000 | -1.813541000 |
| H | 1.486646000 | 1.998334000 | 0.060880000 |
| H | 0.575801000 | 3.510983000 | 0.308176000 |
| C | 1.729631000 | 4.241602000 | -2.374051000 |
| H | 3.576434000 | 5.136558000 | -1.533278000 |
| H | 3.711939000 | 3.406782000 | -1.973122000 |
| H | -0.136669000 | 3.148961000 | -1.954737000 |
| H | 1.259279000 | 2.116257000 | -2.304037000 |

| | | | |
|---|---|---|---|
| H | 1.211286000 | 5.180347000 | -2.152040000 |
| H | 1.890026000 | 4.182618000 | -3.453812000 |
| N | 2.599146000 | 3.803437000 | -0.210093000 |
| H | 2.411142000 | 4.721785000 | 0.335933000 |

## TS1-DA

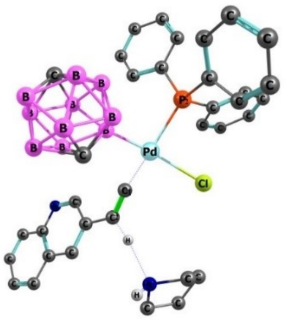

| Atom | x | y | z |
|---|---|---|---|
| Pd | 0.423242000 | -0.153848000 | 0.052033000 |
| P | 2.896611000 | -0.365403000 | -0.067921000 |
| C | 3.440646000 | -2.097285000 | -0.416175000 |
| C | 4.515677000 | -2.702305000 | 0.247803000 |
| H | 5.017482000 | -2.187768000 | 1.060849000 |
| C | 4.945229000 | -3.979119000 | -0.124873000 |
| H | 5.776583000 | -4.439750000 | 0.402039000 |

| | | | |
|---|---|---|---|
| C | 4.311278000 | -4.658260000 | -1.165812000 |
| H | 4.646636000 | -5.650830000 | -1.453981000 |
| C | 3.239184000 | -4.058964000 | -1.832894000 |
| H | 2.734032000 | -4.584588000 | -2.638534000 |
| C | 2.802974000 | -2.789578000 | -1.457320000 |
| H | 2.054879000 | 1.030177000 | -2.474315000 |
| C | 3.841650000 | 0.515734000 | -1.385668000 |
| C | 3.139512000 | 1.059050000 | -2.470009000 |
| H | 1.954422000 | -2.341835000 | -1.966487000 |
| C | 3.823632000 | 1.633790000 | -3.544221000 |
| H | 3.264029000 | 2.051061000 | -4.376762000 |
| C | 5.218206000 | 1.672302000 | -3.544834000 |
| H | 5.751562000 | 2.122385000 | -4.377612000 |
| C | 5.928647000 | 1.118777000 | -2.475486000 |
| H | 7.015102000 | 1.132704000 | -2.476587000 |
| C | 5.247271000 | 0.534101000 | -1.408292000 |
| H | 5.813756000 | 0.083075000 | -0.599293000 |
| C | 3.657426000 | 0.051986000 | 1.555633000 |
| C | 4.537879000 | 1.125303000 | 1.754432000 |
| H | 4.859561000 | 1.734373000 | 0.917171000 |
| C | 5.008603000 | 1.425380000 | 3.035937000 |
| H | 5.690731000 | 2.259990000 | 3.172919000 |
| C | 4.606716000 | 0.660043000 | 4.130772000 |
| H | 4.975634000 | 0.894282000 | 5.125560000 |
| C | 3.722151000 | -0.405906000 | 3.942567000 |
| H | 3.398318000 | -1.003446000 | 4.790287000 |

| | | | |
|---|---|---|---|
| C | 3.243429000 | -0.706011000 | 2.667718000 |
| H | 2.541328000 | -1.524647000 | 2.532330000 |
| H | -0.787529000 | 4.212098000 | 3.101203000 |
| H | -2.615790000 | 4.533800000 | 0.780572000 |
| H | -1.612047000 | 2.035718000 | 1.543767000 |
| H | -1.948418000 | 2.496691000 | -1.211540000 |
| B | -0.385051000 | 4.047906000 | 1.998922000 |
| B | -1.480137000 | 4.242417000 | 0.609213000 |
| C | -0.900573000 | 2.706833000 | 1.078445000 |
| B | -1.068101000 | 2.979461000 | -0.588080000 |
| B | 1.264103000 | 4.330821000 | 1.443042000 |
| H | 2.132904000 | 4.853344000 | 2.056168000 |
| B | -0.083164000 | 5.303998000 | 0.794436000 |
| H | -0.111334000 | 6.474237000 | 0.978167000 |
| C | 1.090918000 | 4.613659000 | -0.229592000 |
| H | 1.802184000 | 5.284377000 | -0.698504000 |
| B | 0.696963000 | 2.665003000 | 1.651536000 |
| H | 1.013386000 | 1.954807000 | 2.544613000 |
| B | 1.668045000 | 3.081150000 | 0.238583000 |
| H | 2.805593000 | 2.821695000 | 0.063831000 |
| B | -0.508991000 | 4.641802000 | -0.807705000 |
| H | -0.822077000 | 5.372046000 | -1.686550000 |
| B | 0.572695000 | 3.262589000 | -1.150572000 |
| H | 0.972015000 | 3.126636000 | -2.256136000 |
| B | 0.292448000 | 1.963583000 | 0.039202000 |
| C | -5.361319000 | 0.948368000 | -3.420641000 |

| | | | |
|---|---|---|---|
| C | -5.929827000 | 0.568238000 | -2.163445000 |
| C | -7.337496000 | 0.644612000 | -1.988736000 |
| C | -8.144232000 | 1.080614000 | -3.017130000 |
| C | -7.579772000 | 1.457635000 | -4.261650000 |
| C | -6.217082000 | 1.392768000 | -4.460642000 |
| N | -4.013847000 | 0.892319000 | -3.654341000 |
| C | -3.230308000 | 0.483975000 | -2.684467000 |
| C | -3.685031000 | 0.091807000 | -1.385076000 |
| C | -5.045426000 | 0.132829000 | -1.146045000 |
| C | -2.733094000 | -0.354637000 | -0.358148000 |
| C | -1.464180000 | -0.143599000 | -0.197154000 |
| H | -7.766286000 | 0.354912000 | -1.032451000 |
| H | -9.220016000 | 1.137535000 | -2.877276000 |
| H | -8.230164000 | 1.799591000 | -5.061794000 |
| H | -5.763493000 | 1.675572000 | -5.405554000 |
| H | -2.163792000 | 0.432350000 | -2.901441000 |
| H | -5.445215000 | -0.154551000 | -0.177646000 |
| Cl | 0.330264000 | -2.501363000 | 0.989206000 |
| H | -4.437452000 | -1.227106000 | 2.374073000 |
| C | -5.231902000 | -2.855093000 | 1.343885000 |
| C | -3.135950000 | -2.845707000 | 2.482991000 |
| C | -4.623420000 | -4.277995000 | 1.224650000 |
| H | -6.051171000 | -2.842983000 | 2.076463000 |
| H | -5.636304000 | -2.491948000 | 0.393292000 |
| C | -3.140097000 | -4.114456000 | 1.628112000 |
| H | -2.153465000 | -2.366222000 | 2.517672000 |

| | | | |
|---|---|---|---|
| H | -3.461511000 | -3.065205000 | 3.513322000 |
| H | -4.730899000 | -4.684829000 | 0.214972000 |
| H | -5.138556000 | -4.962992000 | 1.906063000 |
| H | -2.507252000 | -3.955557000 | 0.749566000 |
| H | -2.748436000 | -4.980890000 | 2.168661000 |
| N | -4.109316000 | -1.984397000 | 1.777962000 |
| H | -3.163140000 | -1.026825000 | 0.465688000 |

Int1-DA

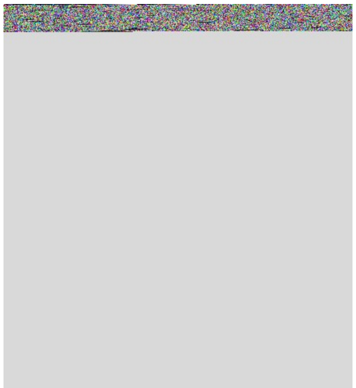

| Atom | x | y | z |
|---|---|---|---|
| Pd | 0.462195000 | -0.845118000 | 0.541678000 |
| P | 2.888732000 | -1.015069000 | 0.903859000 |
| C | 3.528093000 | -2.734948000 | 1.179002000 |
| C | 4.312312000 | -3.097276000 | 2.280622000 |
| C | 4.833927000 | -4.390970000 | 2.383184000 |
| C | 4.585131000 | -5.331420000 | 1.384303000 |
| C | 3.806346000 | -4.975244000 | 0.278810000 |
| C | 3.278251000 | -3.689767000 | 0.180195000 |
| C | 4.107869000 | -0.499884000 | -0.385883000 |
| C | 3.653413000 | -0.309421000 | -1.697241000 |
| C | 4.553373000 | -0.035876000 | -2.730801000 |

| | | | |
|---|---|---|---|
| C | 5.919716000 | 0.051090000 | -2.462970000 |
| C | 6.386115000 | -0.154663000 | -1.160945000 |
| C | 5.489456000 | -0.440047000 | -0.131627000 |
| C | 3.299123000 | -0.125158000 | 2.465929000 |
| C | 4.182427000 | 0.959789000 | 2.553021000 |
| C | 4.371559000 | 1.623131000 | 3.769102000 |
| C | 3.684446000 | 1.211018000 | 4.910802000 |
| C | 2.795981000 | 0.134491000 | 4.832246000 |
| C | 2.596518000 | -0.523737000 | 3.619302000 |
| B | -0.560793000 | 3.775931000 | 0.952620000 |
| B | -1.363558000 | 3.482255000 | -0.607586000 |
| C | -0.947344000 | 2.199367000 | 0.435537000 |
| B | -0.784385000 | 1.907538000 | -1.230004000 |
| B | 1.172662000 | 3.902045000 | 0.663980000 |
| B | 0.013921000 | 4.580890000 | -0.509571000 |
| C | 1.332057000 | 3.623635000 | -1.008762000 |
| B | 0.508886000 | 2.379556000 | 1.285544000 |
| B | 1.744418000 | 2.337249000 | 0.030602000 |
| B | -0.126368000 | 3.423191000 | -1.860656000 |
| B | 0.940803000 | 2.033209000 | -1.523159000 |
| B | 0.387786000 | 1.170112000 | -0.053506000 |
| C | -6.920097000 | -0.660076000 | -0.362942000 |
| C | -6.414404000 | -0.416481000 | 0.954330000 |
| C | -7.331940000 | -0.151393000 | 2.007456000 |
| C | -8.687574000 | -0.129579000 | 1.763257000 |
| C | -9.186345000 | -0.371707000 | 0.458213000 |

| | | | |
|---|---|---|---|
| C | -8.321092000 | -0.631535000 | -0.583174000 |
| N | -6.091873000 | -0.919365000 | -1.421511000 |
| C | -4.796872000 | -0.939323000 | -1.203815000 |
| C | -4.171491000 | -0.719336000 | 0.070069000 |
| C | -5.012742000 | -0.458133000 | 1.144025000 |
| C | -2.749664000 | -0.773838000 | 0.193523000 |
| C | -1.520696000 | -0.780152000 | 0.298014000 |
| Cl | 0.250462000 | -3.170028000 | 1.814658000 |
| H | -1.232394000 | -3.814260000 | 0.623204000 |
| C | -3.191500000 | -4.664499000 | 0.553783000 |
| C | -1.635728000 | -4.521347000 | -1.348477000 |
| C | -3.164186000 | -5.994744000 | -0.203991000 |
| H | -3.035241000 | -4.746510000 | 1.630333000 |
| H | -4.113475000 | -4.109758000 | 0.366777000 |
| C | -2.709778000 | -5.583802000 | -1.614856000 |
| H | -1.558346000 | -3.752321000 | -2.118013000 |
| H | -0.650063000 | -4.964087000 | -1.189939000 |
| H | -4.141221000 | -6.484575000 | -0.192876000 |
| H | -2.436938000 | -6.676687000 | 0.251215000 |
| H | -3.546415000 | -5.148414000 | -2.172871000 |
| H | -2.312953000 | -6.418480000 | -2.198414000 |
| N | -2.049175000 | -3.874152000 | -0.043109000 |
| H | -2.291438000 | -2.873922000 | -0.160678000 |

## TS2-DA

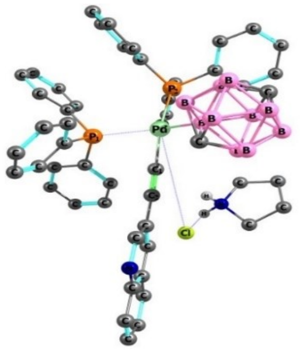


| Atom | x | y | z |
|---|---|---|---|
| Pd | 1.558339000 | 0.125539000 | 0.216736000 |
| P | 3.998774000 | 0.098971000 | 0.405039000 |
| C | 4.733702000 | 0.527243000 | -1.240108000 |
| C | 6.124943000 | 0.518977000 | -1.450106000 |
| H | 6.798366000 | 0.273613000 | -0.634529000 |
| C | 6.654280000 | 0.800201000 | -2.709460000 |
| H | 7.731403000 | 0.797784000 | -2.852036000 |
| C | 5.804171000 | 1.067899000 | -3.786777000 |
| H | 6.218867000 | 1.278706000 | -4.768561000 |
| C | 4.421826000 | 1.045535000 | -3.599766000 |
| H | 3.749858000 | 1.234037000 | -4.432203000 |
| C | 3.891994000 | 0.776386000 | -2.334297000 |
| H | 3.175528000 | 0.641944000 | 3.096073000 |
| C | 4.637406000 | 1.307306000 | 1.647232000 |
| C | 4.014636000 | 1.304812000 | 2.908182000 |
| H | 2.815129000 | 0.754415000 | -2.202132000 |
| C | 4.467086000 | 2.143505000 | 3.927627000 |
| H | 3.979367000 | 2.121505000 | 4.898306000 |
| C | 5.536439000 | 3.011599000 | 3.696267000 |
| H | 5.886480000 | 3.669412000 | 4.486967000 |

| | | | |
|---|---|---|---|
| C | 6.145944000 | 3.037518000 | 2.441015000 |
| H | 6.967756000 | 3.721170000 | 2.247613000 |
| C | 5.703207000 | 2.190010000 | 1.422768000 |
| H | 6.182685000 | 2.235681000 | 0.452154000 |
| C | 4.995522000 | -1.426967000 | 0.760658000 |
| C | 5.862664000 | -1.535697000 | 1.856374000 |
| H | 5.951824000 | -0.719695000 | 2.565358000 |
| C | 6.622953000 | -2.693640000 | 2.045696000 |
| H | 7.291635000 | -2.761060000 | 2.899575000 |
| C | 6.529495000 | -3.752906000 | 1.142610000 |
| H | 7.124598000 | -4.650141000 | 1.288627000 |
| C | 5.668113000 | -3.652496000 | 0.046371000 |
| H | 5.589525000 | -4.471389000 | -0.663494000 |
| C | 4.905350000 | -2.500414000 | -0.140536000 |
| H | 4.241662000 | -2.433488000 | -0.998179000 |
| H | -2.134085000 | -3.684038000 | 2.242702000 |
| H | -1.394421000 | -1.851114000 | 4.466969000 |
| H | -1.242156000 | -1.070306000 | 1.775334000 |
| H | 0.726231000 | -0.062648000 | 3.516329000 |
| B | -1.003505000 | -3.371807000 | 2.411531000 |
| B | -0.560976000 | -2.275768000 | 3.739422000 |
| C | -0.491594000 | -1.772106000 | 2.113516000 |
| B | 0.722578000 | -1.184733000 | 3.133962000 |
| B | 0.410364000 | -4.345282000 | 2.005865000 |
| H | 0.401705000 | -5.478799000 | 1.658612000 |
| B | 0.057865000 | -3.930385000 | 3.705578000 |

| | | | |
|---|---|---|---|
| H | -0.180490000 | -4.787032000 | 4.489024000 |
| C | 1.621824000 | -3.766283000 | 3.049223000 |
| H | 2.378403000 | -4.470502000 | 3.376920000 |
| B | 0.011068000 | -2.944144000 | 1.002305000 |
| H | -0.478347000 | -3.022862000 | -0.070293000 |
| B | 1.692284000 | -3.251830000 | 1.413148000 |
| H | 2.524160000 | -3.698943000 | 0.700950000 |
| B | 1.123970000 | -2.575247000 | 4.159824000 |
| H | 1.596682000 | -2.527348000 | 5.245696000 |
| B | 2.131083000 | -2.166087000 | 2.747826000 |
| H | 3.259446000 | -1.878745000 | 2.944329000 |
| B | 1.106895000 | -1.566961000 | 1.405970000 |
| C | -5.511547000 | -0.021387000 | -2.077637000 |
| C | -4.497377000 | 0.003598000 | -3.086647000 |
| C | -4.879268000 | 0.026800000 | -4.456009000 |
| C | -6.212401000 | 0.028310000 | -4.804215000 |
| C | -7.217086000 | 0.006884000 | -3.803563000 |
| C | -6.875141000 | -0.018051000 | -2.467913000 |
| N | -5.205979000 | -0.048987000 | -0.743062000 |
| C | -3.938923000 | -0.056300000 | -0.398574000 |
| C | -2.836022000 | -0.033640000 | -1.317474000 |
| C | -3.143829000 | 0.002249000 | -2.671781000 |
| C | -1.494938000 | -0.045963000 | -0.823325000 |
| C | -0.350236000 | -0.018049000 | -0.369543000 |
| H | -4.101019000 | 0.037255000 | -5.214955000 |
| H | -6.500033000 | 0.045150000 | -5.851831000 |

| | | | |
|---|---|---|---|
| H | -8.263546000 | 0.009236000 | -4.096406000 |
| H | -7.628040000 | -0.036015000 | -1.685616000 |
| H | -3.722266000 | -0.077453000 | 0.669281000 |
| H | -2.360695000 | 0.029137000 | -3.426313000 |
| P | 1.335311000 | 2.742148000 | -0.396956000 |
| C | 2.642916000 | 4.025068000 | -0.073650000 |
| C | 3.698446000 | 4.158746000 | -0.994256000 |
| H | 3.738516000 | 3.523229000 | -1.873220000 |
| C | 4.689840000 | 5.121374000 | -0.808162000 |
| H | 5.489014000 | 5.215724000 | -1.538703000 |
| C | 4.653253000 | 5.963626000 | 0.306415000 |
| H | 5.423482000 | 6.716841000 | 0.448934000 |
| C | 3.617438000 | 5.832949000 | 1.231374000 |
| H | 3.574396000 | 6.485417000 | 2.099479000 |
| C | 2.618642000 | 4.873090000 | 1.043350000 |
| H | 1.812295000 | 4.801692000 | 1.765188000 |
| C | -0.028131000 | 3.251155000 | 0.744064000 |
| C | 0.049651000 | 2.845870000 | 2.087843000 |
| H | 0.893088000 | 2.250234000 | 2.425611000 |
| C | -0.950911000 | 3.192661000 | 2.996324000 |
| H | -0.871283000 | 2.874533000 | 4.032371000 |
| C | -2.056693000 | 3.933720000 | 2.570929000 |
| H | -2.842163000 | 4.195307000 | 3.274594000 |
| C | -2.149726000 | 4.329271000 | 1.235868000 |
| H | -3.009370000 | 4.899513000 | 0.894427000 |
| C | -1.141087000 | 3.995108000 | 0.327978000 |

| | | | |
|---|---|---|---|
| H | -1.231202000 | 4.310876000 | -0.705743000 |
| C | 0.772095000 | 3.250530000 | -2.082151000 |
| C | 0.373322000 | 2.273556000 | -3.006059000 |
| H | 0.368417000 | 1.228085000 | -2.716962000 |
| C | -0.040835000 | 2.631080000 | -4.292494000 |
| H | -0.337443000 | 1.848556000 | -4.986078000 |
| C | -0.063169000 | 3.975395000 | -4.666582000 |
| H | -0.382877000 | 4.256070000 | -5.666659000 |
| C | 0.328576000 | 4.960283000 | -3.755142000 |
| H | 0.312939000 | 6.008645000 | -4.041576000 |
| C | 0.747624000 | 4.602151000 | -2.473878000 |
| H | 1.061981000 | 5.375972000 | -1.780455000 |
| Cl | -0.686683000 | -0.759637000 | -5.627917000 |
| H | -0.445015000 | -1.773228000 | -2.176834000 |
| C | -1.208716000 | -3.440066000 | -3.250094000 |
| C | 1.113211000 | -2.809189000 | -3.157353000 |
| C | -0.477741000 | -4.628742000 | -2.612191000 |
| H | -2.178036000 | -3.205952000 | -2.808101000 |
| H | -1.331170000 | -3.554594000 | -4.330066000 |
| C | 1.034333000 | -4.271451000 | -2.669852000 |
| H | 1.398048000 | -2.734754000 | -4.209224000 |
| H | 1.771047000 | -2.171345000 | -2.564872000 |
| H | -0.706515000 | -5.553122000 | -3.148794000 |
| H | -0.798797000 | -4.762107000 | -1.575992000 |
| H | 1.578902000 | -4.923859000 | -3.357462000 |
| H | 1.492584000 | -4.377882000 | -1.683471000 |

| | | | |
|---|---|---|---|
| N | -0.286219000 | -2.268362000 | -3.068554000 |
| H | -0.447444000 | -1.581908000 | -3.876888000 |

## TS1-I

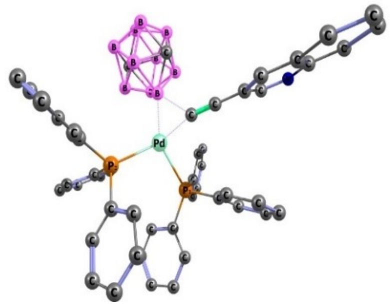

| Atom | x | y | z |
|---|---|---|---|
| Cl | -0.168915000 | -3.548686000 | -2.268068000 |
| Pd | 0.353982000 | -0.042268000 | 0.237773000 |
| P | 2.075986000 | 0.553326000 | 1.893343000 |
| P | -1.058671000 | 0.820188000 | -1.573293000 |
| C | 3.462100000 | 1.225240000 | 0.857343000 |
| C | 3.157174000 | 2.180754000 | -0.129665000 |
| H | 2.125976000 | 2.465817000 | -0.314133000 |
| C | 4.163050000 | 2.750739000 | -0.910565000 |
| H | 3.903734000 | 3.492147000 | -1.661150000 |
| C | 5.491226000 | 2.352248000 | -0.744508000 |
| H | 6.274421000 | 2.789068000 | -1.357555000 |

| | | | |
|---|---|---|---|
| C | 5.801339000 | 1.376340000 | 0.202812000 |
| H | 6.828082000 | 1.044182000 | 0.329070000 |
| C | 4.797572000 | 0.817988000 | 0.999132000 |
| H | 5.066572000 | 0.060995000 | 1.726190000 |
| C | 1.753987000 | 1.957956000 | 3.075398000 |
| C | 1.604456000 | 1.736130000 | 4.454317000 |
| H | 1.715686000 | 0.739415000 | 4.862986000 |
| C | 1.320838000 | 2.791954000 | 5.323691000 |
| H | 1.215570000 | 2.593099000 | 6.386724000 |
| C | 1.178376000 | 4.090311000 | 4.834668000 |
| H | 0.960279000 | 4.911362000 | 5.511759000 |
| C | 1.325774000 | 4.325286000 | 3.466162000 |
| H | 1.229720000 | 5.332859000 | 3.070675000 |
| C | 1.607499000 | 3.271095000 | 2.596373000 |
| H | 1.726733000 | 3.489957000 | 1.542413000 |
| C | 2.837197000 | -0.732688000 | 2.973225000 |
| C | 2.342369000 | -2.043685000 | 2.917077000 |
| H | 1.522112000 | -2.280070000 | 2.248283000 |
| C | 2.889493000 | -3.047162000 | 3.720068000 |
| H | 2.492312000 | -4.056436000 | 3.662864000 |
| C | 3.937317000 | -2.750711000 | 4.592150000 |
| H | 4.363834000 | -3.530357000 | 5.217069000 |
| C | 4.432691000 | -1.445574000 | 4.666217000 |
| H | 5.243191000 | -1.206716000 | 5.349094000 |
| C | 3.884939000 | -0.441780000 | 3.867524000 |
| H | 4.269057000 | 0.570472000 | 3.949538000 |

| | | | |
|---|---|---|---|
| C | -0.178174000 | 0.800194000 | -3.209343000 |
| C | -0.299534000 | -0.281503000 | -4.096224000 |
| H | -0.910695000 | -1.140172000 | -3.847847000 |
| C | 0.397471000 | -0.283750000 | -5.306761000 |
| H | 0.289417000 | -1.130894000 | -5.978020000 |
| C | 1.228331000 | 0.784518000 | -5.649036000 |
| H | 1.765731000 | 0.779585000 | -6.593362000 |
| C | 1.368489000 | 1.856847000 | -4.765388000 |
| H | 2.016325000 | 2.692446000 | -5.016554000 |
| C | 0.676123000 | 1.862721000 | -3.553967000 |
| H | 0.795981000 | 2.707971000 | -2.884956000 |
| C | -2.730334000 | 0.124295000 | -1.965259000 |
| C | -2.839803000 | -1.262810000 | -2.177289000 |
| H | -1.970199000 | -1.917535000 | -2.101346000 |
| C | -4.077966000 | -1.831868000 | -2.484528000 |
| H | -4.137612000 | -2.903469000 | -2.652891000 |
| C | -5.222816000 | -1.036853000 | -2.563488000 |
| H | -6.185503000 | -1.483691000 | -2.796420000 |
| C | -5.124663000 | 0.335902000 | -2.329570000 |
| H | -6.010702000 | 0.963361000 | -2.374991000 |
| C | -3.889287000 | 0.915095000 | -2.031068000 |
| H | -3.842060000 | 1.981311000 | -1.844151000 |
| C | -1.328235000 | 2.635760000 | -1.339009000 |
| C | -1.846043000 | 3.431406000 | -2.378662000 |
| H | -2.097855000 | 2.982131000 | -3.334267000 |
| C | -2.030868000 | 4.802160000 | -2.200021000 |

| | | | |
|---|---|---|---|
| H | -2.434863000 | 5.399795000 | -3.012402000 |
| C | -1.693581000 | 5.404260000 | -0.984241000 |
| H | -1.836048000 | 6.472700000 | -0.847563000 |
| C | -1.170386000 | 4.627783000 | 0.049572000 |
| H | -0.903640000 | 5.084901000 | 0.998366000 |
| C | -0.989479000 | 3.253123000 | -0.127282000 |
| H | -0.587233000 | 2.653533000 | 0.682397000 |
| H | -3.751076000 | -3.026002000 | 2.180135000 |
| H | -5.138458000 | -0.472623000 | 1.541417000 |
| H | -2.794219000 | -1.260239000 | 0.251734000 |
| H | -3.127896000 | 1.495460000 | 0.754362000 |
| B | -3.260824000 | -1.990071000 | 2.477828000 |
| B | -4.091861000 | -0.462319000 | 2.095631000 |
| C | -2.670238000 | -0.931968000 | 1.275811000 |
| B | -2.849437000 | 0.722741000 | 1.602183000 |
| B | -2.192996000 | -1.703844000 | 3.850346000 |
| H | -1.945396000 | -2.466545000 | 4.722636000 |
| B | -3.788184000 | -0.910628000 | 3.774137000 |
| H | -4.608753000 | -1.148947000 | 4.595060000 |
| C | -2.379924000 | -0.041361000 | 4.181141000 |
| H | -2.267396000 | 0.278877000 | 5.210907000 |
| B | -1.509945000 | -1.731995000 | 2.214007000 |
| H | -0.869688000 | -2.623703000 | 1.768078000 |
| B | -0.968152000 | -0.511521000 | 3.358438000 |
| H | 0.058292000 | -0.518054000 | 3.937450000 |
| B | -3.533707000 | 0.769241000 | 3.229809000 |

| | | | |
|---|---|---|---|
| H | -4.186668000 | 1.645903000 | 3.687393000 |
| B | -1.783428000 | 1.018357000 | 2.969383000 |
| H | -1.306863000 | 2.055731000 | 3.280933000 |
| B | -1.215777000 | -0.038637000 | 1.650306000 |
| H | 1.144873000 | -1.750747000 | -1.700905000 |
| C | 2.653286000 | -2.254496000 | -0.483465000 |
| C | 2.818924000 | -0.673147000 | -2.223092000 |
| C | 3.487181000 | -2.946113000 | -1.569703000 |
| H | 3.300520000 | -1.770919000 | 0.255486000 |
| H | 1.966877000 | -2.932661000 | 0.026847000 |
| C | 3.860317000 | -1.790035000 | -2.524209000 |
| H | 2.258759000 | -0.373872000 | -3.106754000 |
| H | 3.310015000 | 0.211963000 | -1.816153000 |
| H | 2.856485000 | -3.679453000 | -2.081060000 |
| H | 4.361851000 | -3.464127000 | -1.161627000 |
| H | 3.823943000 | -2.104104000 | -3.571835000 |
| H | 4.874352000 | -1.424651000 | -2.327244000 |
| N | 1.871836000 | -1.212309000 | -1.201490000 |

## Int1-I

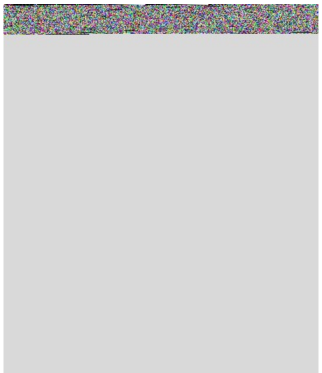

| Atom | x | y | z |
|---|---|---|---|
| Pd | 0.318816000 | -0.201273000 | 0.396722000 |
| P | 2.031376000 | 0.435276000 | 2.020047000 |
| P | -1.110934000 | 0.514428000 | -1.471979000 |
| C | 3.316133000 | 1.269968000 | 0.973551000 |
| C | 2.887399000 | 2.165173000 | -0.022844000 |
| H | 1.825906000 | 2.333505000 | -0.181463000 |
| C | 3.810902000 | 2.843285000 | -0.820305000 |
| H | 3.459837000 | 3.539976000 | -1.576608000 |
| C | 5.179529000 | 2.624161000 | -0.647922000 |
| H | 5.899518000 | 3.147063000 | -1.270705000 |
| C | 5.615594000 | 1.727281000 | 0.328744000 |
| H | 6.677585000 | 1.545972000 | 0.467815000 |
| C | 4.693261000 | 1.056263000 | 1.135283000 |
| H | 5.057586000 | 0.357322000 | 1.878780000 |
| C | 1.694514000 | 1.725945000 | 3.309623000 |
| C | 1.621058000 | 1.414312000 | 4.676603000 |
| H | 1.799612000 | 0.402468000 | 5.020055000 |
| C | 1.325425000 | 2.403200000 | 5.617897000 |
| H | 1.280971000 | 2.140405000 | 6.671048000 |
| C | 1.093819000 | 3.717354000 | 5.211642000 |
| H | 0.865886000 | 4.485384000 | 5.945016000 |
| C | 1.163836000 | 4.038927000 | 3.854295000 |
| H | 0.994584000 | 5.060331000 | 3.525156000 |
| C | 1.460016000 | 3.053801000 | 2.912443000 |
| H | 1.518605000 | 3.335548000 | 1.867129000 |

| | | | |
|---|---|---|---|
| C | 2.895300000 | -0.920795000 | 2.911837000 |
| C | 2.523377000 | -2.245558000 | 2.633714000 |
| H | 1.721360000 | -2.440671000 | 1.928318000 |
| C | 3.162773000 | -3.313556000 | 3.266882000 |
| H | 2.859450000 | -4.332287000 | 3.043355000 |
| C | 4.180485000 | -3.070059000 | 4.190275000 |
| H | 4.676818000 | -3.899440000 | 4.685720000 |
| C | 4.553664000 | -1.755578000 | 4.483560000 |
| H | 5.339590000 | -1.559735000 | 5.207169000 |
| C | 3.915688000 | -0.686996000 | 3.852885000 |
| H | 4.207698000 | 0.327715000 | 4.104991000 |
| C | -0.223387000 | 0.368726000 | -3.099227000 |
| C | -0.676876000 | -0.430065000 | -4.159763000 |
| H | -1.603516000 | -0.985350000 | -4.073424000 |
| C | 0.047760000 | -0.503860000 | -5.353510000 |
| H | -0.324792000 | -1.122730000 | -6.164786000 |
| C | 1.232434000 | 0.215611000 | -5.508086000 |
| H | 1.791052000 | 0.156992000 | -6.437562000 |
| C | 1.691333000 | 1.020152000 | -4.461206000 |
| H | 2.607166000 | 1.593630000 | -4.572261000 |
| C | 0.971059000 | 1.096233000 | -3.268941000 |
| H | 1.338240000 | 1.735179000 | -2.471350000 |
| C | -2.761539000 | -0.230922000 | -1.820612000 |
| C | -2.850539000 | -1.629011000 | -1.967693000 |
| H | -1.961721000 | -2.247619000 | -1.871497000 |
| C | -4.076736000 | -2.240667000 | -2.229647000 |

| | | | |
|---|---|---|---|
| H | -4.125844000 | -3.319324000 | -2.346486000 |
| C | -5.236641000 | -1.467337000 | -2.329930000 |
| H | -6.192947000 | -1.943036000 | -2.526080000 |
| C | -5.161450000 | -0.083563000 | -2.168416000 |
| H | -6.059746000 | 0.523061000 | -2.236519000 |
| C | -3.933274000 | 0.533950000 | -1.916610000 |
| H | -3.896059000 | 1.610425000 | -1.793568000 |
| C | -1.377152000 | 2.338569000 | -1.385558000 |
| C | -1.778948000 | 3.054264000 | -2.528547000 |
| H | -1.942195000 | 2.535987000 | -3.468679000 |
| C | -1.965922000 | 4.434809000 | -2.467234000 |
| H | -2.279899000 | 4.974494000 | -3.356013000 |
| C | -1.744790000 | 5.121217000 | -1.269543000 |
| H | -1.887068000 | 6.197177000 | -1.225574000 |
| C | -1.336837000 | 4.421402000 | -0.133523000 |
| H | -1.160299000 | 4.947969000 | 0.800003000 |
| C | -1.153946000 | 3.037246000 | -0.190007000 |
| H | -0.837562000 | 2.498679000 | 0.696706000 |
| H | -2.844691000 | -1.441955000 | 0.435639000 |
| B | -3.264057000 | -2.208069000 | 2.655755000 |
| B | -4.096955000 | -0.670657000 | 2.315787000 |
| C | -2.693827000 | -1.131607000 | 1.460476000 |
| B | -2.859653000 | 0.518190000 | 1.814062000 |
| B | -2.165952000 | -1.950067000 | 4.010261000 |
| B | -3.759184000 | -1.148390000 | 3.980045000 |
| C | -2.338192000 | -0.292439000 | 4.371113000 |

| | | | |
|---|---|---|---|
| H | -2.203734000 | 0.010305000 | 5.403552000 |
| B | -1.517591000 | -1.952320000 | 2.360173000 |
| B | -0.944243000 | -0.755745000 | 3.512926000 |
| B | -3.510044000 | 0.540166000 | 3.457584000 |
| B | -1.764128000 | 0.786742000 | 3.167550000 |
| B | -1.232036000 | -0.249287000 | 1.823783000 |
| C | 1.295681000 | -2.630027000 | -1.387900000 |
| C | 3.318035000 | -1.489240000 | -0.982503000 |
| C | 2.421387000 | -3.469745000 | -2.056683000 |
| H | 0.993151000 | -3.069350000 | -0.432540000 |
| H | 0.402852000 | -2.535974000 | -2.010396000 |
| C | 3.659899000 | -2.541362000 | -2.047127000 |
| H | 3.852186000 | -0.545844000 | -1.100427000 |
| H | 3.514345000 | -1.874292000 | 0.022980000 |
| H | 2.150721000 | -3.773977000 | -3.071719000 |
| H | 2.606577000 | -4.382345000 | -1.481854000 |
| H | 3.783435000 | -2.053883000 | -3.021471000 |
| H | 4.588879000 | -3.074796000 | -1.824590000 |
| N | 1.853044000 | -1.267263000 | -1.124219000 |

# In1-RE2

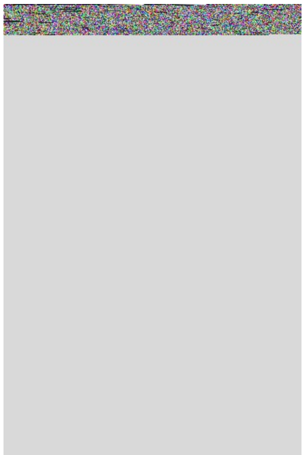

| Atom | x | y | z |
|---|---|---|---|
| Pd | 0.702087000 | 1.104661000 | -1.214817000 |
| P | 2.634564000 | 0.351679000 | 0.103898000 |
| C | 3.203773000 | -1.391616000 | -0.226690000 |
| C | 2.864920000 | -2.475676000 | 0.595995000 |
| H | 2.271376000 | -2.319855000 | 1.490147000 |
| C | 3.295329000 | -3.768925000 | 0.283752000 |
| H | 3.026644000 | -4.593627000 | 0.938418000 |
| C | 4.069983000 | -3.999940000 | -0.853282000 |
| H | 4.405706000 | -5.005058000 | -1.092751000 |
| C | 4.419162000 | -2.926264000 | -1.676958000 |
| H | 5.031980000 | -3.090526000 | -2.559151000 |
| C | 3.991417000 | -1.635185000 | -1.365682000 |
| H | 3.414702000 | 2.555616000 | -1.608776000 |
| C | 4.238945000 | 1.232731000 | -0.124763000 |
| C | 4.310275000 | 2.262922000 | -1.071868000 |
| H | 4.291234000 | -0.809884000 | -2.004985000 |

| | | | |
|---|---|---|---|
| C | 5.522540000 | 2.908313000 | -1.332981000 |
| H | 5.559015000 | 3.705472000 | -2.070158000 |
| C | 6.677868000 | 2.527518000 | -0.650800000 |
| H | 7.620463000 | 3.029689000 | -0.850553000 |
| C | 6.623767000 | 1.485217000 | 0.280249000 |
| H | 7.524285000 | 1.171388000 | 0.800939000 |
| C | 5.417376000 | 0.833704000 | 0.533186000 |
| H | 5.399882000 | 0.000574000 | 1.229250000 |
| C | 2.253140000 | 0.304042000 | 1.911709000 |
| C | 3.115321000 | 0.752967000 | 2.922246000 |
| H | 4.073184000 | 1.193649000 | 2.671054000 |
| C | 2.742919000 | 0.658776000 | 4.265312000 |
| H | 3.420372000 | 1.019546000 | 5.034398000 |
| C | 1.509892000 | 0.108678000 | 4.619205000 |
| H | 1.224422000 | 0.034695000 | 5.664818000 |
| C | 0.638976000 | -0.333089000 | 3.620847000 |
| H | -0.330095000 | -0.747919000 | 3.883747000 |
| C | 1.002206000 | -0.223634000 | 2.277761000 |
| H | 0.302962000 | -0.540431000 | 1.507720000 |
| H | -2.254510000 | 4.611589000 | 1.997478000 |
| H | -2.117916000 | 5.977057000 | -0.637951000 |
| H | -1.908004000 | 3.196795000 | -0.403128000 |
| H | -0.351513000 | 4.427038000 | -2.393433000 |
| B | -1.234698000 | 4.644158000 | 1.393770000 |
| B | -1.152110000 | 5.461604000 | -0.183471000 |
| C | -1.053760000 | 3.763420000 | -0.055832000 |

| | | | |
|---|---|---|---|
| B | -0.087415000 | 4.491096000 | -1.243657000 |
| B | 0.383249000 | 4.639228000 | 2.095786000 |
| H | 0.649991000 | 4.748920000 | 3.245800000 |
| B | -0.194221000 | 6.053671000 | 1.175686000 |
| H | -0.309114000 | 7.104810000 | 1.711734000 |
| C | 1.348051000 | 5.381947000 | 0.907098000 |
| H | 2.207755000 | 5.950016000 | 1.244736000 |
| B | -0.219134000 | 3.175146000 | 1.297749000 |
| H | -0.587702000 | 2.209717000 | 1.874287000 |
| B | 1.446388000 | 3.684198000 | 1.030182000 |
| H | 2.420890000 | 3.203707000 | 1.496042000 |
| B | 0.513689000 | 5.958049000 | -0.459911000 |
| H | 0.869727000 | 6.946032000 | -1.010146000 |
| B | 1.524532000 | 4.490552000 | -0.548574000 |
| H | 2.547137000 | 4.565729000 | -1.139297000 |
| B | 0.510162000 | 3.021504000 | -0.344899000 |
| C | -5.327681000 | 2.455797000 | -5.310645000 |
| C | -5.473514000 | 1.523038000 | -4.234687000 |
| C | -6.731052000 | 0.890644000 | -4.035922000 |
| C | -7.793927000 | 1.171660000 | -4.866419000 |
| C | -7.647578000 | 2.095986000 | -5.931397000 |
| C | -6.439921000 | 2.724718000 | -6.148922000 |
| N | -4.145399000 | 3.099920000 | -5.558083000 |
| C | -3.124250000 | 2.843069000 | -4.773406000 |
| C | -3.146920000 | 1.929825000 | -3.665030000 |
| C | -4.345809000 | 1.276286000 | -3.415908000 |

| | | | |
|---|---|---|---|
| C | -1.973565000 | 1.730548000 | -2.879661000 |
| C | -0.945670000 | 1.606812000 | -2.215463000 |
| H | -6.839439000 | 0.183186000 | -3.217148000 |
| H | -8.751974000 | 0.684447000 | -4.707076000 |
| H | -8.495347000 | 2.307251000 | -6.577286000 |
| H | -6.304381000 | 3.436312000 | -6.957860000 |
| H | -2.194091000 | 3.368180000 | -4.987717000 |
| H | -4.423391000 | 0.574568000 | -2.589313000 |
| H | -0.487525000 | -0.525031000 | -2.843362000 |
| C | 1.278295000 | -1.102130000 | -3.677439000 |
| C | 0.139268000 | -2.231837000 | -1.942511000 |
| C | 0.977491000 | -2.538014000 | -4.199563000 |
| H | 1.084376000 | -0.317703000 | -4.413461000 |
| H | 2.319805000 | -1.018418000 | -3.361444000 |
| C | -0.028403000 | -3.129499000 | -3.180064000 |
| H | 1.000624000 | -2.543939000 | -1.347449000 |
| H | -0.738809000 | -2.200897000 | -1.290875000 |
| H | 1.898419000 | -3.129420000 | -4.229743000 |
| H | 0.564925000 | -2.525602000 | -5.212776000 |
| H | 0.160007000 | -4.185478000 | -2.961887000 |
| H | -1.053590000 | -3.048026000 | -3.560730000 |
| N | 0.402753000 | -0.880211000 | -2.492865000 |

## TS1-RE2

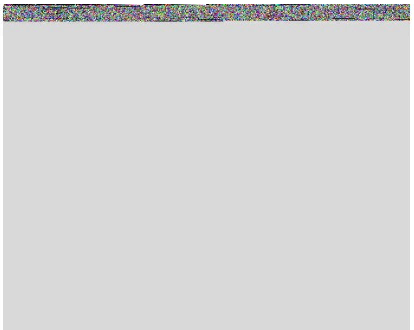

| Atom | x | y | z |
|---|---|---|---|
| Pd | 0.458548000 | 1.017737000 | -1.187826000 |
| P | 2.465797000 | 0.315004000 | 0.050021000 |
| C | 2.971237000 | -1.463538000 | -0.152471000 |
| C | 2.634616000 | -2.455838000 | 0.779794000 |
| H | 2.115434000 | -2.190381000 | 1.694951000 |
| C | 2.969720000 | -3.793637000 | 0.549425000 |
| H | 2.703882000 | -4.546070000 | 1.287195000 |
| C | 3.648222000 | -4.161104000 | -0.613301000 |
| H | 3.911328000 | -5.200539000 | -0.788949000 |
| C | 3.992644000 | -3.180209000 | -1.547379000 |
| H | 4.528120000 | -3.452206000 | -2.453134000 |
| C | 3.655373000 | -1.845610000 | -1.320077000 |
| H | 3.045693000 | 2.635687000 | -1.579387000 |
| C | 4.050343000 | 1.170652000 | -0.363514000 |
| C | 4.004009000 | 2.293160000 | -1.202453000 |
| H | 3.942044000 | -1.094418000 | -2.050795000 |
| C | 5.176256000 | 2.964042000 | -1.561694000 |
| H | 5.121811000 | 3.831124000 | -2.214216000 |

| | | | |
|---|---|---|---|
| C | 6.410386000 | 2.516641000 | -1.088722000 |
| H | 7.323080000 | 3.035897000 | -1.368256000 |
| C | 6.471202000 | 1.390011000 | -0.262785000 |
| H | 7.430627000 | 1.030016000 | 0.098820000 |
| C | 5.301707000 | 0.717718000 | 0.092605000 |
| H | 5.365351000 | -0.170720000 | 0.714059000 |
| C | 2.263049000 | 0.468151000 | 1.883919000 |
| C | 3.267837000 | 0.911002000 | 2.756552000 |
| H | 4.237282000 | 1.205451000 | 2.370037000 |
| C | 3.028353000 | 0.994564000 | 4.130261000 |
| H | 3.815944000 | 1.347211000 | 4.790807000 |
| C | 1.786597000 | 0.629331000 | 4.653384000 |
| H | 1.603597000 | 0.693948000 | 5.722421000 |
| C | 0.776513000 | 0.192028000 | 3.793526000 |
| H | -0.197759000 | -0.082140000 | 4.188749000 |
| C | 1.009275000 | 0.124117000 | 2.418915000 |
| H | 0.207588000 | -0.186270000 | 1.753324000 |
| H | -2.631041000 | 4.758255000 | 1.993579000 |
| H | -2.741336000 | 6.137882000 | -0.636468000 |
| H | -2.640206000 | 3.356888000 | -0.419549000 |
| H | -1.273143000 | 4.550249000 | -2.589754000 |
| B | -1.687126000 | 4.752139000 | 1.276752000 |
| B | -1.751896000 | 5.581849000 | -0.294978000 |
| C | -1.720663000 | 3.877405000 | -0.185496000 |
| B | -0.866663000 | 4.579859000 | -1.480691000 |
| B | -0.001176000 | 4.671776000 | 1.780611000 |

| | | | |
|---|---|---|---|
| H | 0.404668000 | 4.752876000 | 2.891230000 |
| B | -0.617850000 | 6.118872000 | 0.942910000 |
| H | -0.625900000 | 7.169639000 | 1.491078000 |
| C | 0.848995000 | 5.381696000 | 0.490720000 |
| H | 1.769411000 | 5.905255000 | 0.724585000 |
| B | -0.757499000 | 3.240021000 | 1.051911000 |
| H | -1.098338000 | 2.282945000 | 1.659794000 |
| B | 0.885451000 | 3.679769000 | 0.593407000 |
| H | 1.886569000 | 3.169138000 | 0.955107000 |
| B | -0.107876000 | 6.005021000 | -0.764536000 |
| H | 0.220030000 | 6.981419000 | -1.351185000 |
| B | 0.812475000 | 4.487993000 | -0.983345000 |
| H | 1.759276000 | 4.510660000 | -1.695097000 |
| B | -0.242421000 | 3.059184000 | -0.682315000 |
| C | -5.761609000 | 2.175667000 | -4.856423000 |
| C | -4.915388000 | 1.061444000 | -5.159151000 |
| C | -5.318763000 | 0.142057000 | -6.165727000 |
| C | -6.507517000 | 0.320865000 | -6.838695000 |
| C | -7.344493000 | 1.424816000 | -6.536727000 |
| C | -6.979294000 | 2.333457000 | -5.566274000 |
| N | -5.432396000 | 3.097638000 | -3.900207000 |
| C | -4.304515000 | 2.938954000 | -3.247829000 |
| C | -3.376670000 | 1.861364000 | -3.455122000 |
| C | -3.710876000 | 0.929258000 | -4.429176000 |
| C | -2.184066000 | 1.778067000 | -2.680136000 |
| C | -1.167006000 | 1.784808000 | -1.979991000 |

| | | | |
|---|---|---|---|
| H | -4.674226000 | -0.703544000 | -6.393884000 |
| H | -6.809301000 | -0.386352000 | -7.606310000 |
| H | -8.278763000 | 1.551314000 | -7.076638000 |
| H | -7.603811000 | 3.186388000 | -5.318521000 |
| H | -4.066536000 | 3.690990000 | -2.496994000 |
| H | -3.048556000 | 0.091903000 | -4.633816000 |
| H | -0.865910000 | -0.605736000 | -2.779349000 |
| C | 0.828039000 | -1.248431000 | -3.710370000 |
| C | -0.214593000 | -2.350212000 | -1.930358000 |
| C | 0.230782000 | -2.523037000 | -4.346179000 |
| H | 0.801544000 | -0.370446000 | -4.361495000 |
| H | 1.865403000 | -1.420340000 | -3.406890000 |
| C | -0.354832000 | -3.313820000 | -3.141599000 |
| H | 0.647107000 | -2.625183000 | -1.316902000 |
| H | -1.096285000 | -2.326957000 | -1.283738000 |
| H | 0.980389000 | -3.094841000 | -4.902569000 |
| H | -0.563283000 | -2.254036000 | -5.052091000 |
| H | 0.187073000 | -4.247739000 | -2.961915000 |
| H | -1.402720000 | -3.576581000 | -3.318880000 |
| N | 0.030402000 | -0.998624000 | -2.488842000 |

## Int2-RE2

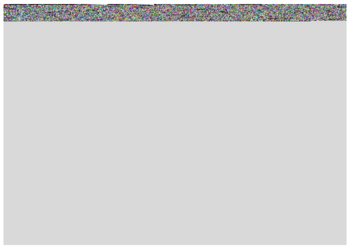

| Atom | x | y | z |
|---|---|---|---|
| Pd | 2.700852000 | -1.306725000 | 0.017222000 |
| P | 0.823429000 | -2.646671000 | -0.373900000 |
| P | 4.728294000 | -0.238066000 | 0.410274000 |
| C | 0.799656000 | -4.162445000 | 0.686744000 |
| C | 1.291689000 | -4.047556000 | 1.997810000 |
| H | 1.689532000 | -3.093064000 | 2.334136000 |
| C | 1.287243000 | -5.145988000 | 2.858059000 |
| H | 1.670277000 | -5.040426000 | 3.869558000 |
| C | 0.805188000 | -6.380369000 | 2.414840000 |
| H | 0.810146000 | -7.239075000 | 3.080845000 |
| C | 0.325510000 | -6.508859000 | 1.109841000 |
| H | -0.044696000 | -7.467838000 | 0.756856000 |
| C | 0.320085000 | -5.407121000 | 0.250464000 |
| H | -0.053711000 | -5.521219000 | -0.762535000 |
| C | -0.850853000 | -1.906537000 | -0.102778000 |
| C | -1.919674000 | -2.599007000 | 0.486253000 |
| H | -1.784649000 | -3.622095000 | 0.823155000 |
| C | -3.162387000 | -1.979947000 | 0.647182000 |
| H | -3.979708000 | -2.528025000 | 1.108936000 |
| C | -3.356003000 | -0.665782000 | 0.218170000 |
| H | -4.324402000 | -0.187855000 | 0.340070000 |
| C | -2.296218000 | 0.033775000 | -0.366459000 |
| H | -2.438178000 | 1.060081000 | -0.693942000 |
| C | -1.052375000 | -0.579150000 | -0.516933000 |
| H | -0.224450000 | -0.028115000 | -0.956767000 |

| | | | |
|---|---|---|---|
| C | 0.711412000 | -3.317781000 | -2.094895000 |
| C | 1.913970000 | -3.564144000 | -2.778377000 |
| H | 2.857891000 | -3.332464000 | -2.290378000 |
| C | 1.902723000 | -4.086581000 | -4.072131000 |
| H | 2.841558000 | -4.272843000 | -4.586906000 |
| C | 0.687885000 | -4.356273000 | -4.707793000 |
| H | 0.677681000 | -4.754458000 | -5.718888000 |
| C | -0.513401000 | -4.103716000 | -4.042333000 |
| H | -1.461705000 | -4.305273000 | -4.533640000 |
| C | -0.503401000 | -3.589275000 | -2.743251000 |
| H | -1.444501000 | -3.394438000 | -2.237816000 |
| C | 5.959597000 | -1.262140000 | 1.336214000 |
| C | 5.971581000 | -2.644255000 | 1.083001000 |
| H | 5.250847000 | -3.063098000 | 0.384897000 |
| C | 6.885424000 | -3.476995000 | 1.729006000 |
| H | 6.882898000 | -4.543658000 | 1.521410000 |
| C | 7.790140000 | -2.942880000 | 2.650456000 |
| H | 8.495770000 | -3.592332000 | 3.161473000 |
| C | 7.777212000 | -1.573172000 | 2.919953000 |
| H | 8.472566000 | -1.152481000 | 3.641506000 |
| C | 6.869160000 | -0.735902000 | 2.266332000 |
| H | 6.867830000 | 0.326532000 | 2.490223000 |
| C | 5.653627000 | 0.269415000 | -1.110983000 |
| C | 7.052268000 | 0.373054000 | -1.172343000 |
| H | 7.653856000 | 0.129866000 | -0.301419000 |
| C | 7.682338000 | 0.775606000 | -2.352518000 |

| | | | |
|---|---|---|---|
| H | 8.766102000 | 0.848393000 | -2.385843000 |
| C | 6.925278000 | 1.077697000 | -3.486590000 |
| H | 7.418106000 | 1.386584000 | -4.404394000 |
| C | 5.533280000 | 0.965911000 | -3.441142000 |
| H | 4.939526000 | 1.179246000 | -4.326104000 |
| C | 4.903363000 | 0.557320000 | -2.263708000 |
| H | 3.822929000 | 0.433167000 | -2.234492000 |
| C | 4.646042000 | 1.325108000 | 1.404881000 |
| C | 5.505820000 | 2.423136000 | 1.221810000 |
| H | 6.276287000 | 2.388824000 | 0.457420000 |
| C | 5.372743000 | 3.570019000 | 2.012596000 |
| H | 6.034576000 | 4.416054000 | 1.850368000 |
| C | 4.383238000 | 3.636433000 | 2.996731000 |
| H | 4.266743000 | 4.536814000 | 3.591253000 |
| C | 3.526601000 | 2.548868000 | 3.190369000 |
| H | 2.747995000 | 2.598107000 | 3.946249000 |
| C | 3.651579000 | 1.406921000 | 2.396845000 |
| H | 2.967282000 | 0.572232000 | 2.529964000 |
| C | -3.570728000 | 4.756309000 | -0.876625000 |
| C | -3.537190000 | 4.139336000 | 0.416452000 |
| C | -2.512575000 | 4.501990000 | 1.333584000 |
| C | -1.559573000 | 5.432505000 | 0.981715000 |
| C | -1.595162000 | 6.042919000 | -0.299297000 |
| C | -2.579003000 | 5.715518000 | -1.208307000 |
| N | -4.526684000 | 4.448446000 | -1.807040000 |
| C | -5.434343000 | 3.553669000 | -1.493114000 |

| | | | |
|---|---|---|---|
| C | -5.494996000 | 2.868109000 | -0.235831000 |
| C | -4.532877000 | 3.180514000 | 0.713985000 |
| C | -6.509924000 | 1.896676000 | 0.000437000 |
| C | -7.379059000 | 1.061936000 | 0.182121000 |
| H | -2.494745000 | 4.030923000 | 2.313589000 |
| H | -0.761556000 | 5.707195000 | 1.664978000 |
| H | -0.828484000 | 6.770779000 | -0.547332000 |
| H | -2.626713000 | 6.174352000 | -2.191241000 |
| H | -6.184879000 | 3.323864000 | -2.248092000 |
| H | -4.539099000 | 2.689517000 | 1.683265000 |
| H | -8.662559000 | -3.871207000 | 0.017382000 |
| H | -10.417118000 | -2.457480000 | -1.913231000 |
| H | -7.833940000 | -1.536699000 | -1.307430000 |
| H | -9.775192000 | 0.431313000 | -1.782922000 |
| B | -9.108075000 | -2.808772000 | 0.291110000 |
| B | -10.163034000 | -1.958176000 | -0.870114000 |
| C | -8.582858000 | -1.409751000 | -0.533647000 |
| B | -9.779402000 | -0.218346000 | -0.793813000 |
| B | -9.493773000 | -2.228077000 | 1.914806000 |
| H | -9.492391000 | -2.874382000 | 2.906913000 |
| B | -10.788396000 | -2.454235000 | 0.706741000 |
| H | -11.643259000 | -3.249747000 | 0.901533000 |
| C | -10.687101000 | -1.035679000 | 1.653232000 |
| H | -11.432952000 | -0.902655000 | 2.428898000 |
| B | -8.071597000 | -1.594656000 | 1.085145000 |
| H | -6.942742000 | -1.850609000 | 1.334047000 |

| | | | |
|---|---|---|---|
| B | -9.108639000 | -0.487471000 | 1.993669000 |
| H | -8.852302000 | 0.016418000 | 3.033464000 |
| B | -11.201959000 | -0.852680000 | 0.035875000 |
| H | -12.329356000 | -0.590625000 | -0.213267000 |
| B | -10.166975000 | 0.364329000 | 0.830483000 |
| H | -10.606024000 | 1.428297000 | 1.105558000 |
| B | -8.472212000 | 0.025960000 | 0.416416000 |
| Cl | 2.058891000 | 6.432257000 | 1.089320000 |
| H | 3.328899000 | 3.293567000 | 0.295618000 |
| C | 1.313270000 | 3.006779000 | -0.318966000 |
| C | 3.043711000 | 4.186227000 | -1.596602000 |
| C | 0.941946000 | 3.043438000 | -1.813541000 |
| H | 1.486646000 | 1.998334000 | 0.060880000 |
| H | 0.575801000 | 3.510983000 | 0.308176000 |
| C | 1.729631000 | 4.241602000 | -2.374051000 |
| H | 3.576434000 | 5.136558000 | -1.533278000 |
| H | 3.711939000 | 3.406782000 | -1.973122000 |
| H | -0.136669000 | 3.148961000 | -1.954737000 |
| H | 1.259279000 | 2.116257000 | -2.304037000 |
| H | 1.211286000 | 5.180347000 | -2.152040000 |
| H | 1.890026000 | 4.182618000 | -3.453812000 |
| N | 2.599146000 | 3.803437000 | -0.210093000 |
| H | 2.411142000 | 4.721785000 | 0.335933000 |

3-quinolylethynyl magnesium bromide

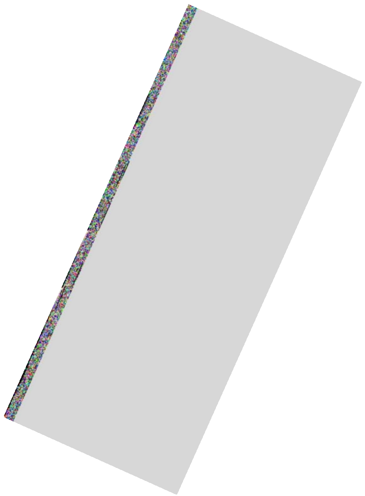

| **Atom** | **x** | **y** | **z** |
| --- | --- | --- | --- |
| C | -2.947818000 | 0.749374000 | -0.000015000 |
| C | -2.518262000 | -0.616641000 | 0.000137000 |
| C | -3.495331000 | -1.648778000 | 0.000062000 |
| C | -4.837341000 | -1.338336000 | -0.000204000 |
| C | -5.260752000 | 0.014723000 | -0.000403000 |
| C | -4.336460000 | 1.037341000 | -0.000301000 |

| | | | |
|---|---|---|---|
| N | -2.061170000 | 1.792113000 | 0.000084000 |
| C | -0.778864000 | 1.513839000 | 0.000236000 |
| C | -0.230956000 | 0.187691000 | 0.000327000 |
| C | -1.126424000 | -0.871307000 | 0.000299000 |
| C | 1.182871000 | 0.008976000 | 0.000328000 |
| C | 2.405913000 | -0.105406000 | 0.000309000 |
| H | -3.165729000 | -2.684929000 | 0.000226000 |
| H | -5.579158000 | -2.131986000 | -0.000263000 |
| H | -6.323060000 | 0.242482000 | -0.000646000 |
| H | -4.637402000 | 2.080387000 | -0.000434000 |
| H | -0.090271000 | 2.357795000 | 0.000343000 |
| H | -0.763881000 | -1.895676000 | 0.000405000 |
| Mg | 4.434598000 | -0.135932000 | -0.000024000 |
| Cl | 6.672533000 | -0.145326000 | -0.000270000 |

## Monomeric $MgCl_2$ (Int3-K)

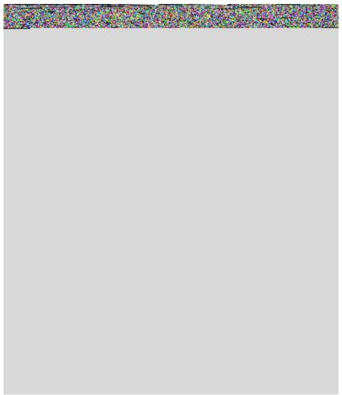

| Atom | x | y | z |
|---|---|---|---|
| Pd | -0.740052000 | 0.759982000 | -0.036383000 |
| P | 0.250188000 | -1.865737000 | 0.409769000 |

| | | | |
|---|---|---|---|
| P | -3.144366000 | 0.332603000 | 0.130460000 |
| C | -0.632201000 | -3.202942000 | 1.353983000 |
| C | -1.615888000 | -3.948073000 | 0.678865000 |
| H | -1.823993000 | -3.752456000 | -0.368372000 |
| C | -2.327581000 | -4.949898000 | 1.338269000 |
| H | -3.080204000 | -5.515179000 | 0.795896000 |
| C | -2.080362000 | -5.220092000 | 2.686637000 |
| H | -2.637282000 | -5.999392000 | 3.199631000 |
| C | -1.111075000 | -4.483227000 | 3.368463000 |
| H | -0.903012000 | -4.689701000 | 4.415015000 |
| C | -0.389967000 | -3.483917000 | 2.708264000 |
| H | 0.369324000 | -2.934090000 | 3.253373000 |
| C | 1.003873000 | -2.843919000 | -0.963909000 |
| C | 1.553852000 | -4.121980000 | -0.756119000 |
| H | 1.540205000 | -4.565046000 | 0.235324000 |
| C | 2.113350000 | -4.832996000 | -1.817852000 |
| H | 2.536276000 | -5.818524000 | -1.642392000 |
| C | 2.123168000 | -4.283364000 | -3.103682000 |
| H | 2.553565000 | -4.841664000 | -3.930677000 |
| C | 1.566604000 | -3.022550000 | -3.322993000 |
| H | 1.551171000 | -2.593124000 | -4.320873000 |
| C | 1.008836000 | -2.306462000 | -2.259046000 |
| H | 0.563779000 | -1.336598000 | -2.452531000 |
| C | 1.662223000 | -1.443048000 | 1.528163000 |
| C | 1.390470000 | -0.628433000 | 2.642610000 |
| H | 0.375687000 | -0.284036000 | 2.826704000 |

| | | | |
|---|---|---|---|
| C | 2.409525000 | -0.251872000 | 3.518513000 |
| H | 2.177641000 | 0.368036000 | 4.380507000 |
| C | 3.724447000 | -0.663262000 | 3.280997000 |
| H | 4.520943000 | -0.362449000 | 3.955769000 |
| C | 4.007263000 | -1.457871000 | 2.169788000 |
| H | 5.026994000 | -1.778010000 | 1.973815000 |
| C | 2.983861000 | -1.849818000 | 1.301351000 |
| H | 3.223917000 | -2.469853000 | 0.444502000 |
| C | -3.855646000 | 0.170253000 | 1.823352000 |
| C | -4.819953000 | 1.046919000 | 2.340933000 |
| H | -5.224833000 | 1.841211000 | 1.724094000 |
| C | -5.270833000 | 0.907652000 | 3.656644000 |
| H | -6.017475000 | 1.596900000 | 4.041114000 |
| C | -4.768747000 | -0.108279000 | 4.470063000 |
| H | -5.122432000 | -0.215596000 | 5.491675000 |
| C | -3.807811000 | -0.987545000 | 3.963045000 |
| H | -3.411070000 | -1.785377000 | 4.584672000 |
| C | -3.349227000 | -0.845803000 | 2.653839000 |
| H | -2.602997000 | -1.539040000 | 2.278153000 |
| C | -4.253875000 | 1.476376000 | -0.804602000 |
| C | -5.644002000 | 1.267105000 | -0.841475000 |
| H | -6.090723000 | 0.456738000 | -0.273300000 |
| C | -6.461488000 | 2.090270000 | -1.614814000 |
| H | -7.534602000 | 1.920962000 | -1.629563000 |
| C | -5.902103000 | 3.121213000 | -2.376041000 |
| H | -6.540393000 | 3.757267000 | -2.982940000 |

| | | | |
|---|---|---|---|
| C | -4.522046000 | 3.323594000 | -2.361665000 |
| H | -4.076469000 | 4.114878000 | -2.957601000 |
| C | -3.703426000 | 2.505067000 | -1.579363000 |
| H | -2.631840000 | 2.668437000 | -1.570642000 |
| C | -3.573909000 | -1.263802000 | -0.702034000 |
| C | -4.554930000 | -2.139775000 | -0.214851000 |
| H | -5.041880000 | -1.943982000 | 0.734800000 |
| C | -4.910431000 | -3.275714000 | -0.946647000 |
| H | -5.673306000 | -3.945589000 | -0.559041000 |
| C | -4.291276000 | -3.548851000 | -2.168106000 |
| H | -4.571021000 | -4.431965000 | -2.736027000 |
| C | -3.313785000 | -2.678915000 | -2.659404000 |
| H | -2.828961000 | -2.878679000 | -3.610805000 |
| C | -2.955940000 | -1.542367000 | -1.932198000 |
| H | -2.205291000 | -0.869168000 | -2.337299000 |
| C | 6.280822000 | 1.650233000 | -2.338549000 |
| C | 5.597445000 | 0.439881000 | -2.684318000 |
| C | 6.283518000 | -0.557261000 | -3.429179000 |
| C | 7.591038000 | -0.360477000 | -3.814384000 |
| C | 8.266700000 | 0.838238000 | -3.471677000 |
| C | 7.627161000 | 1.822960000 | -2.749343000 |
| N | 5.678955000 | 2.651104000 | -1.624873000 |
| C | 4.431062000 | 2.485234000 | -1.253847000 |
| C | 3.651595000 | 1.315759000 | -1.538395000 |
| C | 4.256413000 | 0.296184000 | -2.259147000 |
| C | 2.300512000 | 1.221395000 | -1.077455000 |

| | | | |
|---|---|---|---|
| C | 1.147057000 | 1.133632000 | -0.645429000 |
| H | 5.758749000 | -1.472944000 | -3.689776000 |
| H | 8.111138000 | -1.124377000 | -4.385220000 |
| H | 9.297623000 | 0.977917000 | -3.784591000 |
| H | 8.125782000 | 2.748243000 | -2.478170000 |
| H | 3.968842000 | 3.295827000 | -0.693180000 |
| H | 3.704620000 | -0.609397000 | -2.497163000 |
| H | 2.061207000 | 4.891431000 | 2.530801000 |
| H | 0.394798000 | 3.466085000 | 4.537188000 |
| H | 1.238249000 | 2.239752000 | 2.163248000 |
| H | -1.318878000 | 1.554793000 | 3.119492000 |
| B | 0.934286000 | 4.621228000 | 2.285339000 |
| B | -0.062711000 | 3.766657000 | 3.486095000 |
| C | 0.466766000 | 2.993119000 | 2.055463000 |
| B | -1.092881000 | 2.599494000 | 2.609684000 |
| B | -0.140350000 | 5.520586000 | 1.213443000 |
| H | 0.080852000 | 6.578351000 | 0.729599000 |
| B | -0.501981000 | 5.394227000 | 2.957473000 |
| H | -0.520558000 | 6.368769000 | 3.630828000 |
| C | -1.698870000 | 5.130714000 | 1.775083000 |
| H | -2.471195000 | 5.883945000 | 1.666694000 |
| B | 0.519147000 | 3.975892000 | 0.672799000 |
| H | 1.380834000 | 3.880203000 | -0.124251000 |
| B | -1.167984000 | 4.349663000 | 0.340208000 |
| H | -1.630793000 | 4.669320000 | -0.700227000 |
| B | -1.755630000 | 4.145117000 | 3.164003000 |

| | | | |
|---|---|---|---|
| H | -2.610706000 | 4.283177000 | 3.972305000 |
| B | -2.166888000 | 3.511293000 | 1.550122000 |
| H | -3.296923000 | 3.270853000 | 1.311427000 |
| B | -0.748694000 | 2.690591000 | 0.844911000 |
| Mg | 0.384317000 | 2.011617000 | -2.630741000 |
| Cl | -0.633214000 | 0.515398000 | -4.066235000 |
| Cl | 0.841539000 | 4.138132000 | -3.345187000 |

## Dimeric $MgCl_2$ (Int3-K)

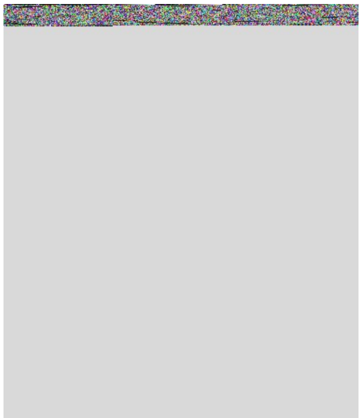

| **Atom** | **x** | **y** | **z** |
|---|---|---|---|
| Pd | -0.822045000 | 0.737503000 | -0.156585000 |
| P | 0.202661000 | -1.900737000 | 0.254848000 |
| P | -3.213718000 | 0.298326000 | 0.167830000 |
| C | -0.647728000 | -3.147889000 | 1.335711000 |
| C | -1.721108000 | -3.887306000 | 0.806534000 |
| H | -2.011617000 | -3.759691000 | -0.231101000 |
| C | -2.416403000 | -4.801163000 | 1.598688000 |

| | | | |
|---|---|---|---|
| H | -3.237932000 | -5.364807000 | 1.165801000 |
| C | -2.065086000 | -4.984411000 | 2.938269000 |
| H | -2.609834000 | -5.693809000 | 3.555114000 |
| C | -1.006829000 | -4.251002000 | 3.476934000 |
| H | -0.717966000 | -4.389923000 | 4.515369000 |
| C | -0.300927000 | -3.342852000 | 2.682934000 |
| H | 0.526277000 | -2.793413000 | 3.118593000 |
| C | 0.788868000 | -2.934672000 | -1.163780000 |
| C | 0.959771000 | -4.326954000 | -1.083585000 |
| H | 0.719714000 | -4.853199000 | -0.165581000 |
| C | 1.434507000 | -5.048083000 | -2.181600000 |
| H | 1.556444000 | -6.125076000 | -2.103588000 |
| C | 1.755548000 | -4.390679000 | -3.371050000 |
| H | 2.126294000 | -4.953004000 | -4.223438000 |
| C | 1.597810000 | -3.005575000 | -3.459610000 |
| H | 1.856829000 | -2.481508000 | -4.375365000 |
| C | 1.109675000 | -2.285472000 | -2.367058000 |
| H | 0.991949000 | -1.210062000 | -2.445495000 |
| C | 1.755384000 | -1.557397000 | 1.204241000 |
| C | 1.706190000 | -0.599336000 | 2.231547000 |
| H | 0.775464000 | -0.078492000 | 2.439233000 |
| C | 2.839413000 | -0.308893000 | 2.992857000 |
| H | 2.779766000 | 0.428336000 | 3.789054000 |
| C | 4.047419000 | -0.958887000 | 2.725478000 |
| H | 4.933293000 | -0.726455000 | 3.309807000 |
| C | 4.109489000 | -1.905769000 | 1.702260000 |

| | | | |
|---|---|---|---|
| H | 5.044907000 | -2.415314000 | 1.487376000 |
| C | 2.971122000 | -2.207913000 | 0.949313000 |
| H | 3.035910000 | -2.952659000 | 0.162804000 |
| C | -3.855408000 | 0.162511000 | 1.893568000 |
| C | -4.857012000 | 0.993891000 | 2.415713000 |
| H | -5.321916000 | 1.748890000 | 1.791952000 |
| C | -5.265818000 | 0.862223000 | 3.745586000 |
| H | -6.041458000 | 1.516812000 | 4.133229000 |
| C | -4.684204000 | -0.101603000 | 4.569579000 |
| H | -5.005180000 | -0.203177000 | 5.602542000 |
| C | -3.685006000 | -0.934525000 | 4.058976000 |
| H | -3.224769000 | -1.690604000 | 4.688840000 |
| C | -3.268544000 | -0.799273000 | 2.734862000 |
| H | -2.490730000 | -1.454728000 | 2.356691000 |
| C | -4.363472000 | 1.427397000 | -0.736500000 |
| C | -5.753402000 | 1.209917000 | -0.730067000 |
| H | -6.177940000 | 0.394744000 | -0.151960000 |
| C | -6.599954000 | 2.030774000 | -1.473628000 |
| H | -7.671998000 | 1.855135000 | -1.454784000 |
| C | -6.070940000 | 3.067895000 | -2.248464000 |
| H | -6.732156000 | 3.702890000 | -2.831546000 |
| C | -4.692296000 | 3.275395000 | -2.280504000 |
| H | -4.270050000 | 4.068717000 | -2.890528000 |
| C | -3.843440000 | 2.458114000 | -1.529068000 |
| H | -2.772225000 | 2.616846000 | -1.566563000 |
| C | -3.694743000 | -1.314674000 | -0.606353000 |

| | | | |
|---|---|---|---|
| C | -4.665826000 | -2.164156000 | -0.056117000 |
| H | -5.111690000 | -1.934906000 | 0.906084000 |
| C | -5.064066000 | -3.316686000 | -0.738379000 |
| H | -5.819583000 | -3.963836000 | -0.301001000 |
| C | -4.496973000 | -3.636124000 | -1.973653000 |
| H | -4.809101000 | -4.532350000 | -2.502552000 |
| C | -3.528861000 | -2.794728000 | -2.528991000 |
| H | -3.084958000 | -3.032364000 | -3.491782000 |
| C | -3.130464000 | -1.641707000 | -1.850518000 |
| H | -2.389290000 | -0.988582000 | -2.301219000 |
| C | 6.287459000 | 1.919813000 | -1.957658000 |
| C | 5.760446000 | 0.601603000 | -2.148669000 |
| C | 6.627913000 | -0.438243000 | -2.580456000 |
| C | 7.960677000 | -0.180110000 | -2.811885000 |
| C | 8.481532000 | 1.125122000 | -2.621660000 |
| C | 7.664397000 | 2.153737000 | -2.203783000 |
| N | 5.507622000 | 2.966469000 | -1.546196000 |
| C | 4.233823000 | 2.741554000 | -1.323288000 |
| C | 3.600088000 | 1.464505000 | -1.476643000 |
| C | 4.384742000 | 0.399406000 | -1.894183000 |
| C | 2.208305000 | 1.311203000 | -1.181731000 |
| C | 1.021775000 | 1.167958000 | -0.863541000 |
| H | 6.221121000 | -1.435744000 | -2.726466000 |
| H | 8.619973000 | -0.977087000 | -3.143568000 |
| H | 9.535101000 | 1.311691000 | -2.809746000 |
| H | 8.043020000 | 3.159978000 | -2.054276000 |

| | | | |
|---|---|---|---|
| H | 3.630562000 | 3.588259000 | -1.000242000 |
| H | 3.946539000 | -0.585294000 | -2.027953000 |
| H | 2.090896000 | 4.996792000 | 2.092677000 |
| H | 0.788567000 | 3.430710000 | 4.259168000 |
| H | 1.375489000 | 2.312277000 | 1.756345000 |
| H | -0.992245000 | 1.452487000 | 3.019696000 |
| B | 0.958182000 | 4.665687000 | 1.990089000 |
| B | 0.179670000 | 3.727028000 | 3.286602000 |
| C | 0.555078000 | 3.019343000 | 1.777627000 |
| B | -0.891981000 | 2.521022000 | 2.517871000 |
| B | -0.299642000 | 5.522915000 | 1.098578000 |
| H | -0.204153000 | 6.602499000 | 0.621637000 |
| B | -0.416826000 | 5.336515000 | 2.869499000 |
| H | -0.400790000 | 6.293518000 | 3.567650000 |
| C | -1.744821000 | 5.029219000 | 1.847299000 |
| H | -2.565038000 | 5.738193000 | 1.864709000 |
| B | 0.365936000 | 4.032217000 | 0.427311000 |
| H | 1.117075000 | 4.006464000 | -0.479217000 |
| B | -1.366169000 | 4.313208000 | 0.331673000 |
| H | -1.977844000 | 4.633094000 | -0.628093000 |
| B | -1.559841000 | 4.009677000 | 3.201421000 |
| H | -2.305852000 | 4.078996000 | 4.119141000 |
| B | -2.147665000 | 3.391974000 | 1.635736000 |
| H | -3.283434000 | 3.090595000 | 1.538473000 |
| B | -0.792585000 | 2.668811000 | 0.728312000 |
| Mg | -0.117171000 | 0.094711000 | -5.787003000 |

| | | | |
|---|---|---|---|
| Mg | 0.547013000 | 2.000697000 | -3.007180000 |
| Cl | 1.903649000 | 0.738053000 | -4.734492000 |
| Cl | -1.495193000 | 0.996334000 | -4.076897000 |
| Cl | -0.511438000 | -1.295533000 | -7.527689000 |
| Cl | 0.554533000 | 4.222547000 | -3.589954000 |

$Mg_2Cl_4$



| **Atom** | **x** | **y** | **z** |
|---|---|---|---|
| Mg | -0.151146000 | -0.176038000 | -5.804098000 |
| Mg | 0.444625000 | 2.428312000 | -3.770335000 |
| Cl | 1.842079000 | 0.973996000 | -5.083918000 |
| Cl | -1.548389000 | 1.278109000 | -4.489965000 |
| Cl | -0.477522000 | -1.911695000 | -7.192356000 |
| Cl | 0.771746000 | 4.164125000 | -2.382543000 |

Int4-K

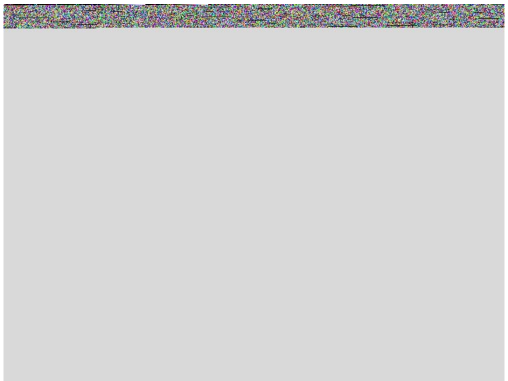

| Atom | x | y | z |
|---|---|---|---|
| Pd | 0.029267000 | 0.172819000 | 1.167525000 |
| Cl | -0.669462000 | -1.937893000 | 2.373443000 |
| P | 1.583572000 | -1.042719000 | -0.155131000 |
| C | -5.710539000 | 0.246672000 | -0.232437000 |
| C | -4.956385000 | -0.885449000 | 0.219821000 |
| C | -5.411919000 | -2.196281000 | -0.090091000 |
| C | -6.566477000 | -2.374700000 | -0.818597000 |
| C | -7.311020000 | -1.253782000 | -1.266482000 |
| C | -6.894508000 | 0.029316000 | -0.981362000 |
| N | -5.329406000 | 1.534791000 | 0.032769000 |
| C | -4.229714000 | 1.726230000 | 0.722516000 |
| C | -3.402015000 | 0.668420000 | 1.221548000 |
| C | -3.781820000 | -0.643219000 | 0.965357000 |
| C | -2.246638000 | 0.964916000 | 1.998700000 |
| C | -1.273566000 | 1.201651000 | 2.711152000 |
| H | -0.707283000 | 1.484732000 | 3.573954000 |
| H | -4.834537000 | -3.048083000 | 0.259497000 |
| H | -6.913320000 | -3.376912000 | -1.053057000 |

| | | | |
|---|---|---|---|
| H | -8.220192000 | -1.412710000 | -1.839717000 |
| H | -7.452206000 | 0.898781000 | -1.314739000 |
| H | -3.944982000 | 2.756805000 | 0.926098000 |
| H | -3.165991000 | -1.461505000 | 1.330628000 |
| H | -0.469254000 | 4.406432000 | -2.592798000 |
| H | 2.435731000 | 3.780922000 | -2.622627000 |
| H | 0.507114000 | 1.867894000 | -1.987296000 |
| H | 2.958016000 | 1.740162000 | -0.607579000 |
| B | 0.102435000 | 4.188607000 | -1.578037000 |
| B | 1.844136000 | 3.815348000 | -1.596744000 |
| C | 0.678406000 | 2.619642000 | -1.227766000 |
| B | 2.136927000 | 2.553146000 | -0.368686000 |
| B | -0.298336000 | 4.854377000 | 0.001940000 |
| H | -1.091539000 | 5.705773000 | 0.225635000 |
| B | 1.255353000 | 5.258663000 | -0.775065000 |
| H | 1.496934000 | 6.380473000 | -1.070685000 |
| C | 1.172166000 | 4.789638000 | 0.860963000 |
| H | 1.343836000 | 5.553107000 | 1.611649000 |
| B | -0.661139000 | 3.150727000 | -0.336735000 |
| H | -1.746203000 | 2.729363000 | -0.555204000 |
| B | 0.011020000 | 3.599544000 | 1.227959000 |
| H | -0.568297000 | 3.684348000 | 2.254615000 |
| B | 2.516523000 | 4.243227000 | -0.023267000 |
| H | 3.596406000 | 4.688369000 | 0.176730000 |
| B | 1.740414000 | 3.209115000 | 1.213137000 |
| H | 2.316251000 | 3.010735000 | 2.230615000 |

| | | | |
|---|---|---|---|
| B | 0.582648000 | 2.088517000 | 0.427308000 |
| C | 1.369203000 | -2.883810000 | -0.187468000 |
| C | 0.870232000 | -3.570829000 | -1.302420000 |
| C | 1.748955000 | -3.618663000 | 0.948900000 |
| C | 0.756841000 | -4.963983000 | -1.283291000 |
| C | 1.642978000 | -5.007391000 | 0.960368000 |
| C | 1.144127000 | -5.685391000 | -0.155016000 |
| H | 0.578756000 | -3.034148000 | -2.197628000 |
| H | 2.124528000 | -3.107208000 | 1.827634000 |
| H | 0.371684000 | -5.480161000 | -2.158571000 |
| H | 1.942127000 | -5.559041000 | 1.847121000 |
| H | 1.058867000 | -6.768636000 | -0.142646000 |
| C | 3.324312000 | -0.909455000 | 0.428645000 |
| C | 3.631725000 | -0.088784000 | 1.522367000 |
| C | 4.345752000 | -1.681322000 | -0.153818000 |
| C | 4.938279000 | -0.019396000 | 2.011082000 |
| C | 5.651655000 | -1.601718000 | 0.328618000 |
| C | 5.950408000 | -0.769057000 | 1.410935000 |
| H | 2.845493000 | 0.492385000 | 1.993694000 |
| H | 4.119723000 | -2.356810000 | -0.973446000 |
| H | 5.160734000 | 0.619267000 | 2.861110000 |
| H | 6.433034000 | -2.198716000 | -0.133284000 |
| H | 6.967153000 | -0.714332000 | 1.790014000 |
| C | 1.536746000 | -0.600409000 | -1.945545000 |
| C | 2.672174000 | -0.303649000 | -2.711369000 |
| C | 0.272044000 | -0.559560000 | -2.562043000 |

| | | | |
|---|---|---|---|
| C | 2.546597000 | 0.016697000 | -4.066244000 |
| C | 0.151179000 | -0.254986000 | -3.918560000 |
| C | 1.290728000 | 0.036225000 | -4.673983000 |
| H | 3.656121000 | -0.308397000 | -2.255961000 |
| H | -0.622256000 | -0.757708000 | -1.975999000 |
| H | 3.435645000 | 0.253479000 | -4.643916000 |
| H | -0.832243000 | -0.233136000 | -4.379477000 |
| H | 1.197639000 | 0.284434000 | -5.727395000 |

## MgCl2



| **Atom** | **x** | **y** | **z** |
|---|---|---|---|
| Mg | 0.000000000 | 0.000000000 | -0.000216000 |
| Cl | 0.000000000 | 0.000000000 | 2.240768000 |
| Cl | 0.000000000 | 0.000000000 | -2.240615000 |

## Int5

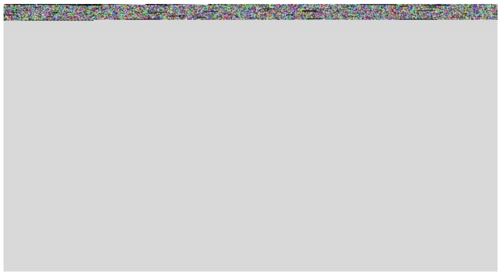

| **Atom** | **x** | **y** | **z** |
|---|---|---|---|
| Pd | 5.033741000 | 0.071932000 | -0.159844000 |

| | | | |
|---|---|---|---|
| P | 4.086605000 | 2.195799000 | -0.029139000 |
| P | 5.970706000 | -2.050704000 | -0.340306000 |
| C | 4.364302000 | 3.265194000 | -1.513979000 |
| C | 4.460638000 | 2.628423000 | -2.762591000 |
| H | 4.395577000 | 1.543947000 | -2.810052000 |
| C | 4.654302000 | 3.371317000 | -3.927735000 |
| H | 4.726146000 | 2.863298000 | -4.885801000 |
| C | 4.770502000 | 4.762039000 | -3.859972000 |
| H | 4.931256000 | 5.341100000 | -4.765487000 |
| C | 4.689780000 | 5.404438000 | -2.622754000 |
| H | 4.787341000 | 6.485289000 | -2.562207000 |
| C | 4.486576000 | 4.662237000 | -1.456570000 |
| H | 4.430878000 | 5.174077000 | -0.500583000 |
| C | 2.247108000 | 2.209320000 | 0.178455000 |
| C | 1.395088000 | 3.129982000 | -0.450253000 |
| H | 1.806624000 | 3.890156000 | -1.107202000 |
| C | 0.013677000 | 3.072301000 | -0.244843000 |
| H | -0.634759000 | 3.787254000 | -0.744823000 |
| C | -0.533172000 | 2.101831000 | 0.596851000 |
| H | -1.608045000 | 2.055674000 | 0.751954000 |
| C | 0.308037000 | 1.180538000 | 1.228623000 |
| H | -0.109881000 | 0.417357000 | 1.879612000 |
| C | 1.685224000 | 1.227315000 | 1.011980000 |
| H | 2.335470000 | 0.492354000 | 1.480431000 |
| C | 4.675022000 | 3.246225000 | 1.376367000 |
| C | 6.001000000 | 3.073441000 | 1.806548000 |

| | | | |
|---|---|---|---|
| H | 6.620458000 | 2.320908000 | 1.324325000 |
| C | 6.516560000 | 3.843347000 | 2.849909000 |
| H | 7.544638000 | 3.697855000 | 3.170859000 |
| C | 5.708562000 | 4.787196000 | 3.488997000 |
| H | 6.106081000 | 5.380678000 | 4.308057000 |
| C | 4.384672000 | 4.957699000 | 3.078667000 |
| H | 3.748608000 | 5.684557000 | 3.577208000 |
| C | 3.870075000 | 4.193374000 | 2.028440000 |
| H | 2.837145000 | 4.331109000 | 1.722633000 |
| C | 7.424561000 | -2.164930000 | -1.480502000 |
| C | 8.269670000 | -1.045786000 | -1.565582000 |
| H | 8.029755000 | -0.155930000 | -0.988384000 |
| C | 9.394177000 | -1.064130000 | -2.391315000 |
| H | 10.038169000 | -0.190357000 | -2.445174000 |
| C | 9.681906000 | -2.196659000 | -3.157506000 |
| H | 10.551966000 | -2.208500000 | -3.808620000 |
| C | 8.841109000 | -3.309448000 | -3.091568000 |
| H | 9.054628000 | -4.190558000 | -3.691165000 |
| C | 7.719990000 | -3.295648000 | -2.257687000 |
| H | 7.071749000 | -4.165988000 | -2.219971000 |
| C | 6.605139000 | -2.775557000 | 1.240180000 |
| C | 7.724606000 | -3.618489000 | 1.318102000 |
| H | 8.279400000 | -3.869963000 | 0.419170000 |
| C | 8.140437000 | -4.132358000 | 2.549165000 |
| H | 9.012069000 | -4.780157000 | 2.594799000 |
| C | 7.442843000 | -3.813556000 | 3.715950000 |

| | | | |
|---|---|---|---|
| H | 7.769521000 | -4.212317000 | 4.672698000 |
| C | 6.329949000 | -2.971179000 | 3.649971000 |
| H | 5.788119000 | -2.710178000 | 4.555171000 |
| C | 5.919565000 | -2.449927000 | 2.422548000 |
| H | 5.069095000 | -1.774042000 | 2.373253000 |
| C | 4.825192000 | -3.357182000 | -0.981349000 |
| C | 4.871229000 | -4.700929000 | -0.579582000 |
| H | 5.599435000 | -5.022672000 | 0.158857000 |
| C | 3.980145000 | -5.633333000 | -1.118134000 |
| H | 4.027365000 | -6.670535000 | -0.796473000 |
| C | 3.034548000 | -5.236108000 | -2.066377000 |
| H | 2.344478000 | -5.962891000 | -2.486783000 |
| C | 2.975782000 | -3.898121000 | -2.468048000 |
| H | 2.237498000 | -3.580039000 | -3.199420000 |
| C | 3.858862000 | -2.965298000 | -1.923126000 |
| H | 3.798944000 | -1.919767000 | -2.216097000 |
| C | -2.502990000 | -3.592551000 | 1.501445000 |
| C | -1.769832000 | -2.678248000 | 0.677846000 |
| C | -0.498675000 | -3.064480000 | 0.172333000 |
| C | 0.021847000 | -4.304204000 | 0.471632000 |
| C | -0.705938000 | -5.208151000 | 1.286481000 |
| C | -1.941324000 | -4.862117000 | 1.790957000 |
| N | -3.730405000 | -3.282917000 | 2.022590000 |
| C | -4.239270000 | -2.103860000 | 1.752244000 |
| C | -3.597022000 | -1.109589000 | 0.943083000 |
| C | -2.355233000 | -1.419019000 | 0.408192000 |

| | | | |
|---|---|---|---|
| C | -4.237745000 | 0.142700000 | 0.716433000 |
| C | -4.812739000 | 1.202713000 | 0.540153000 |
| H | 0.056132000 | -2.367154000 | -0.450367000 |
| H | 0.993260000 | -4.592873000 | 0.080972000 |
| H | -0.281188000 | -6.182118000 | 1.513621000 |
| H | -2.514180000 | -5.539417000 | 2.416724000 |
| H | -5.216045000 | -1.877044000 | 2.177119000 |
| H | -1.827381000 | -0.697935000 | -0.209337000 |
| H | -4.847554000 | 6.260484000 | 1.227761000 |
| H | -7.204347000 | 5.174483000 | 2.667318000 |
| H | -4.861323000 | 3.667972000 | 2.308639000 |
| H | -7.278140000 | 2.247347000 | 2.216333000 |
| B | -5.485779000 | 5.388031000 | 0.744524000 |
| B | -6.902955000 | 4.734638000 | 1.610156000 |
| C | -5.471935000 | 3.823604000 | 1.426184000 |
| B | -6.950349000 | 2.972429000 | 1.340448000 |
| B | -5.713730000 | 5.099373000 | -0.984042000 |
| H | -5.381082000 | 5.819897000 | -1.862807000 |
| B | -7.102943000 | 5.536601000 | 0.047953000 |
| H | -7.688950000 | 6.546050000 | -0.150102000 |
| C | -7.189850000 | 4.246318000 | -1.067949000 |
| H | -7.799004000 | 4.395821000 | -1.952423000 |
| B | -4.657486000 | 4.029768000 | -0.060547000 |
| H | -3.474839000 | 4.002296000 | -0.106661000 |
| B | -5.759937000 | 3.336513000 | -1.256473000 |
| H | -5.458288000 | 2.891405000 | -2.310809000 |

| | | | |
|---|---|---|---|
| B | -8.007060000 | 4.042281000 | 0.416965000 |
| H | -9.189954000 | 4.064656000 | 0.463271000 |
| B | -7.180285000 | 2.682187000 | -0.389088000 |
| H | -7.812413000 | 1.806727000 | -0.873392000 |
| B | -5.552944000 | 2.514363000 | 0.305291000 |
| Mg | -12.153062927 | -0.357922860 | -0.932796397 |
| Cl | -10.239115927 | -0.972688860 | 0.042477603 |
| Cl | -14.066899927 | 0.257421140 | -1.908530397 |
